# Quantifying the Light-Absorption Properties and Molecular Composition of Brown Carbon Aerosol from Sub-Saharan African Biomass Combustion

**DOI:** 10.1021/acs.est.3c09378

**Published:** 2024-02-23

**Authors:** Vaios Moschos, Cade Christensen, Megan Mouton, Marc N. Fiddler, Tommaso Isolabella, Federico Mazzei, Dario Massabò, Barbara J. Turpin, Solomon Bililign, Jason D. Surratt

**Affiliations:** † Department of Physics, College of Science and Technology, North Carolina A&T State University, Greensboro, North Carolina 27411, United States; ‡ Department of Environmental Sciences and Engineering, Gillings School of Global Public Health, The University of North Carolina at Chapel Hill, Chapel Hill, North Carolina 27516, United States; § Department of Chemistry, College of Arts and Sciences, The University of North Carolina at Chapel Hill, Chapel Hill, North Carolina 27599, United States; ∥ Department of Applied Sciences and Technology, College of Science and Technology, North Carolina A&T State University, Greensboro, North Carolina 27411, United States; ⊥ Department of Chemistry, College of Science and Technology, North Carolina A&T State University, Greensboro, North Carolina 27411, United States; # Department of Physics, University of Genoa, 16146 Genoa, Italy; ⬡ National Institute of Nuclear Physics (INFN), 16146 Genoa, Italy

**Keywords:** smoldering combustion, aethalometer, mass-absorption cross-section, emission factors, hierarchical clustering

## Abstract

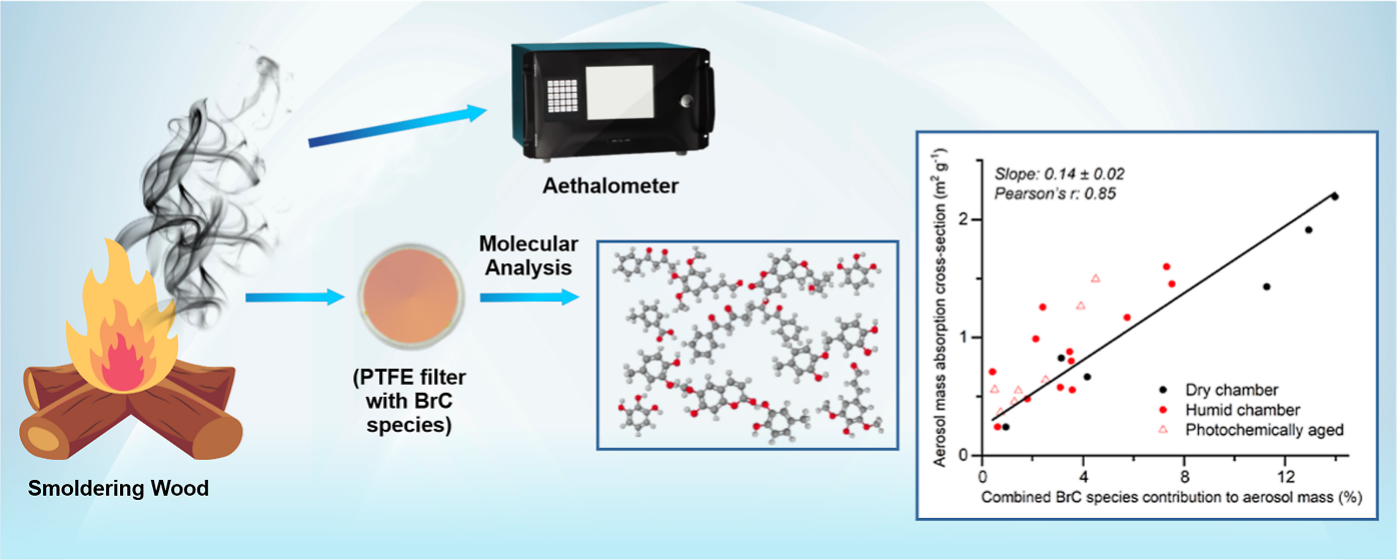

Sub-Saharan Africa is a hotspot for biomass burning (BB)-derived carbonaceous aerosols, including light-absorbing organic (brown) carbon (BrC). However, the chemically complex nature of BrC in BB aerosols from this region is not fully understood. We generated smoke in a chamber through smoldering combustion of common sub-Saharan African biomass fuels (hardwoods, cow dung, savanna grass, and leaves). We quantified aethalometer-based, real-time light-absorption properties of BrC-containing organic-rich BB aerosols, accounting for variations in wavelength, fuel type, relative humidity, and photochemical aging conditions. In filter samples collected from the chamber and Botswana in the winter, we identified 182 BrC species, classified into lignin pyrolysis products, nitroaromatics, coumarins, stilbenes, and flavonoids. Using an extensive set of standards, we determined species-specific mass and emission factors. Our analysis revealed a linear relationship between the combined BrC species contribution to chamber-measured BB aerosol mass (0.4–14%) and the mass-absorption cross-section at 370 nm (0.2–2.2 m^2^ g^–1^). Hierarchical clustering resolved key molecular-level components from the BrC matrix, with photochemically aged emissions from leaf and cow-dung burning showing BrC fingerprints similar to those found in Botswana aerosols. These quantitative findings could potentially help refine climate model predictions, aid in source apportionment, and inform effective air quality management policies for human health and the global climate.

## Abstract 2

We quantitatively link real-time light absorption and offline molecular composition of brown carbon aerosols from sub-Saharan African biomass combustion, informing climate models and policy strategies for air quality and health.

## 1 Introduction

Africa contributes a majority of the global annual atmospheric carbonaceous aerosol (CA) mass from biomass burning (BB) emissions, where CA is the sum of particulate organic and elemental carbon.^1^ BB aerosols persist throughout the Southern Hemisphere’s wintertime,^2^ influencing the climate via aerosol-radiation and aerosol-cloud interactions.^3,4^ In sub-Saharan Africa, the main BB-derived CA sources are residential solid fuel combustion, prescribed fires, and wildfires, particularly in the Congo Basin, west Africa, and southern African savanna grasslands.^5,6^ Solid fuels are used for over two-thirds of household energy consumption in most sub-Saharan African countries,^7^ leading to indoor and outdoor air pollution levels that often exceed the World Health Organization health-based guidelines by a factor of up to 30.^8^ African BB-derived CA is likely to increase with the projected population growth and urbanization, due to widespread poverty and limited clean energy access.^9,10^

Brown carbon (BrC), a subset of light-absorbing organic carbon in CA, is a critically important yet poorly understood component of BB aerosols emitted from solid fuels during smoldering combustion in both natural and residential sectors.^11−13^ The positive direct radiative forcing of BB-derived BrC aerosols in subtropical Africa varies considerably, yet it potentially matches the cooling effect of nonabsorbing organic aerosols (OAs).^14^ Common reasons for negative model bias^15^ in the CA warming effect in southern/sub-Saharan Africa are the neglect of light absorption by BrC aerosols^16−18^ and the scarcity of observational BrC data.^19−22^ In addition, BrC aerosol degrades visibility and CA poses human health risks upon inhalation,^23,24^ including increase in noncommunicable diseases.^25^

Despite its well-recognized impacts, a comprehensive analysis of the complex molecular-level composition and optical properties of (sub-Saharan Africa) BB-derived BrC-containing aerosol remains elusive.^26,27^ Accurate quantification of individual BrC species by mass and determination of their emission factors (EFs) pose substantial challenges.^28,29^ While numerous BrC species and their contributions to absorbance have been identified upon solvent extraction, the use of an extensive array of standard compounds for mass quantification was previously deemed impractical.^28,30^ Additionally, the absorptivity of OAs^31^ and the relative contributions of individual species to BrC (or total aerosol) mass are expected to vary with the specific emission source, combustion efficiency, and atmospheric conditions/processing (i.e., phase partitioning, oxidation, and aging).^13,32−34^ Crucially, a systematic correlation between the molecular-level composition of primary/aged BrC and broad-band optical absorption properties of OAs in the particle phase is currently lacking.^35^

To fill these knowledge gaps, we conducted smog chamber experiments using a range of African biomass fuels that simulate smoldering combustion^36^ and atmospherically relevant relative humidity (∼65% RH). We quantified the real-time optical absorption properties of the generated organic-rich aerosols and examined their dependence on the fuel type, RH, and (photochemical) aging conditions. Using an optimized chromatographic separation protocol and a comprehensive suite of BrC authentic standards, we analyzed filter-collected aerosols for molecular-level absorbance and chemical composition. Furthermore, we correlated the light-absorption properties with the molecular-level composition of the chamber-generated BrC aerosol species and assessed their atmospheric relevance by analyzing airborne particulate matter (PM) collected at two locations in Botswana in the winter.

## 2 Materials and Methods

### 2.1 Sub-Saharan African Biomass Fuel Combustion Chamber Experiments

We used 11 African biomass fuels, including hardwood species (acacia, eucalyptus, mopane, mosetlha, mukusi, wanza, and wild olive), tree branches (from mokala) and leaves (from mopane), cow dung, and savanna grass [Supporting Information (SI) Text S1]. Most fuels are native to Botswana and neighboring countries mainly in eastern and southern Africa (Figure S1) and are frequently used for firewood in this region (Text S1).

We sampled BB primary and aged emissions using an indoor combustion-chamber system at North Carolina A&T State University (NC A&T)^37^ (Text S2). The 9 m^3^ Teflon chamber was either dry (<10% RH) or conditioned to ∼65% RH before each experiment. We combusted ∼0.4 g of each fuel (dried and debarked) in a tube furnace that is uniformly heated at the center over the burning region (Text S2). The ignition temperature was set to 450 °C, yielding smoldering-dominated combustion (fire-average modified combustion efficiency, MCE < 0.9; Table S1) and organic-rich^38^ emissions of PM_2.5_ (particulate matter with an aerodynamic diameter < 2.5 μm or fine aerosol; using a cyclone, Text S2) into the dark chamber.

We defined three aerosol aging conditions within each chamber experiment: (1) primary aerosol sampled within 30 min to 1 h of combustion, (2) dark-aged aerosol sampled 4 h after combustion in the dark chamber, and (3) photochemically aged aerosol (following dark aging) sampled after smoke was irradiated for 2 h with UV lamps (Text S2). No additional oxidants, seed aerosol, or volatile organic compounds were injected into the chamber, apart from those generated during combustion or upon irradiation.

### 2.2 Aethalometer Measurements and Calibration

We quantified the light-absorption properties of BrC-containing OAs generated from the combustion of African biomass fuels using a dual-spot aethalometer (AE33, Magee Scientific). The AE33 system measures light transmission through aerosol-loaded spots on a filter tape (*I*), compared to transmission through a reference obtained by passing clean air through a filter tape spot (*I*_o_). The AE33 simultaneously analyzes at seven optical wavelengths (λ) from the near-infrared (950 nm) to near-UV (370 nm). The optical attenuation (ATN_λ_) is defined as ATN_λ_ = −100 * ln(*I*_λ_/*I*_0,λ_).

In this study, we determined the attenuation coefficient, *b*_atn,λ_ (Mm^–1^), for optically absorbing aerosols by evaluating (see Text S2) the light attenuation rate of change passing through a particle-laden filter. Dynamic blanks were performed by placing a HEPA filter at the inlet, allowing the aethalometer to sample particle-free chamber air; we found that gases made no contribution to ATN_λ_. We conducted three 10–15 min aethalometer measurements within each experiment with one measurement for each aging condition. We employed the formula
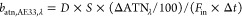
1where *S* represents the spot area, *F*_in_ is the aerosol flow rate, and *t* is the time (1 min resolution; therefore, *b*_atn,AE33,λ_ is the average of 10–15 values at each measurement/aging condition). High chamber-aerosol mass concentrations (*M* ≈ 800 μg m^–3^ for primary emissions) required dilution with zero air, where the dilution ratio (*D*) was 10, using a dilution jar to acquire multiple ATN values for accurate *b*_atn,AE33,λ_ calculations (Text S2).

During aethalometer measurements, we simultaneously collected 47 mm quartz fiber filters (QFFs; Whatman, QMC fired, 1855-047), with a scrubber (Ozone Solutions, Inc.) attached to the inlet to reduce gas-phase adsorption on the QFFs.^39^ For 20 selected QFFs, we determined the aerosol absorption coefficient (*b*_abs_) with a multiwavelength absorbance analyzer (MWAA)^40^ to determine AE33 calibration coefficients: *C*_λ_ = *b*_abs,MWAA,λ_/*b*_abs,AE33,λ_; interpolation of *b*_abs,MWAA_ to AE33 wavelengths was based on adjacent-wavelength absorption Ångström exponents (AAE, ) that assumed a power-law relationship. *C*_λ_ values (dimensionless) were applied to rectify *b*_atn,AE33_ (eq 1) for apparent absorption due to scattering artifacts, such as multiple scattering effects, where we determine the approximate *b*_abs_ for all samples (*b*_abs,AE33,λ_ = *b*_atn,AE33,λ_/*C*_λ_, in Mm^–1^) and assume that MWAA measurements are closer to the true absorption value.^41^ Moschos et al. reported a largely wavelength-independent *C* of ∼2.3 for year-long aerosols in Switzerland that consisted of both elemental and organic carbon.^41^ However, for the chamber African BB-OAs, we found the *C* values to be wavelength-dependent: *C*_λ=370 nm_ = 4.1, *C*_λ=470 nm_ = 3.2, *C*_λ=520 nm_ = 2.9 [relative errors (1σ/mean) ∼ 25%], and *C*_λ=590 nm_ = 2.0, with a decreasing correction factor with increasing wavelength but no systematic variation between fuels or aging conditions. To the best of our knowledge, this is the first study that presents calibration coefficients for the aethalometer *b*_atn_ that vary substantially with the wavelength. This variability significantly affects *b*_abs,λ_ and AAEs, compared to studies that use or assume constant coefficients independent of wavelength and might be related to the distinct spectral scattering properties of African BB-OAs.

We divided *b*_abs,λ_ by the chamber-aerosol *M* (based on scanning mobility particle sizer, SMPS, measurements; Text S2) to estimate the fine aerosol absorption cross section per unit mass or mass-absorption cross-section (MAC, m^2^ g^–1^) of the BrC-containing organic-rich aerosols
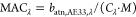
2

### 2.3 Aerosol Filter Collection for Chemical Composition Measurements

For molecular-level BrC analysis, Teflon filter samples (Tisch Scientific, SF18040; 47 mm diameter, 2 μm pore size, 38 mm aerosol collection diameter) were collected from each chamber experiment listed in Table S1. The sampling flow rate and duration were 30 L min^–1^ and 10–20 min, respectively.

We also collected 47 mm Teflon filter samples at the onset of the 2022 wintertime fire/heating season in southern Africa (Botswana). Total suspended PM was sampled from June 24 to July 21 in Gaborone, the capital city, at a time when the PM concentration was high (∼150 μg m^–3^), and at the Botswana International University of Science and Technology (BIUST) weather station near Palapye, which had a lower PM concentration (campaign-average: 10 μg m^–3^). Ten Teflon filter aerosol samples were collected in total, with some day/night filters sampled over two different dates (Table S1). Details of filter sampling (including chamber/field blanks) and sample handling are provided in Text S2.

### 2.4 Molecular-Level Analysis of Chamber and Ambient BrC Aerosols

We employed a multistage analytical platform that combines reversed-phase liquid chromatography (RPLC), diode array detection (DAD), and high-resolution quadrupole time-of-flight tandem mass spectrometry (HR-QTOFMS/MS) with electrospray ionization (ESI) operated in both positive (+) and negative (−) modes. We analyzed methanol extracts of 25 selected chamber and 10 ambient Teflon filter samples (Table S1). The sample preparation protocol and details of the RPLC/DAD-ESI-HR-QTOFMS/MS method, including gradient elution scheme, operating conditions, and data analysis procedures, are described in Text S3.

### 2.5 Quantification of BrC Species with Standards

We made use of 100 authentic standards (100 ppm stock solution in methanol; 25 species/group) for quantification of BrC species. Standard selection was based on our preliminary molecular analysis of chromophoric species in filter samples from test chamber burns, combined with C_6–20_H_H_O_1–10_N_0–2_ BrC formulas (tentative structures) in the literature (see the Laskin-group papers in “References” section). ChemSpider was used to determine if a potential (aromatic) structure would reasonably exhibit absorption in UV. Details of the standard preparation and characterization are provided in Text S4. A sample-average extracted wavelength DAD chromatogram and the mass absorption coefficient spectra of target BrC species in methanol solution (Text S4) are provided in Figures S2 and S3.

Calibration curves were generated by measuring a mixture of the 100 standards, ranging from 0.002 to 10 ppm, at the beginning and end of sample analysis (the respective vials were stored in a freezer during the aerosol-sample analysis). Each standard’s response factor (RF) was determined (Text S4) using the linear-range extracted ion chromatogram (EIC) peak area (*A*) per μg mL^–1^. Positional isomers exhibited distinct retention times (RTs) due to polarity differences, with their RFs differing by a factor of up to 2 (Text S4).

Table S2 displays those standards identified with our analytical platform and used to quantify (Text S4) the mass of the BrC species found in the aerosol samples including the vendor, purity, ESI RF, and fragmentation information (Table S3). The IDs refer to the complete list of 182 BrC species (Table S4). In Text S4, we delineate our systematic approach to quantifying BrC species in aerosol filter samples. Briefly, for BrC species without a matching authentic standard, we assigned surrogate standards based on structural similarity and adjacent RTs (Table S4). We note that during the sample preparation drying step (Text S3), about one-third of the mass evaporated; thus, reported results have been adjusted for recoveries (Text S4). We discuss the strengths and limitations of the methodology for quantification in Text S5.

### 2.6 Hierarchical Cluster Analysis

We applied hierarchical cluster analysis to visualize the complex BrC molecular-level composition matrix in heatmaps (Text S6). Dimensionality reduction through species/sample clustering assisted in identifying: (a) similarities in BrC composition between chamber- and ambient-aerosol filter sample extracts and (b) potential tracer species for particular African fuel types or aging processes.

## 3 Results and Discussion

### 3.1 Light-Absorption Properties of Sub-Saharan African Biomass Fuel-Derived OAs

#### 3.1.1 MAC of Primary OAs

**Figure 1:**
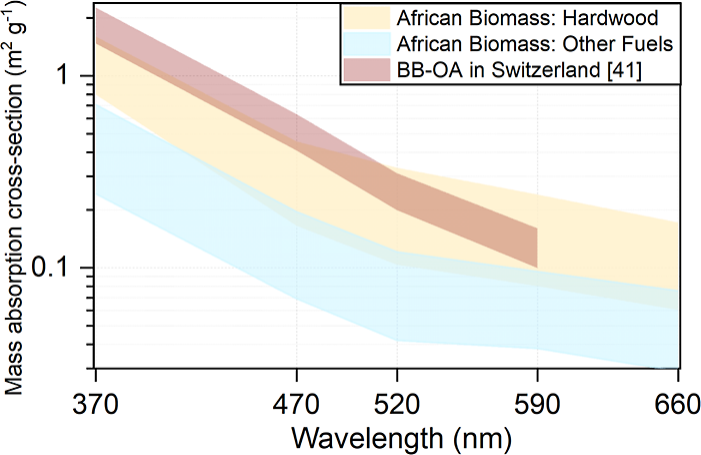
MAC spectral range (shaded areas: min–max) for primary organic-rich aerosols injected into the humid chamber. This was derived from the smoldering combustion of African hardwood species (yellow; *N* = 7) and other biomass fuels (blue; *N* = 4) and quantified (eq 2) by using calibrated aethalometer measurements (Section 2.2). “Other” includes savanna grass, cow dung, and tree leaves/branches. Literature-reported ambient primary BB-OA from Switzerland^41^ (brown) is also shown (see Section 3.1.1).

The light-absorption potential, expressed as the broad-band MAC (MAC_λ_; Section 2.2 and eq 2), of primary BB fine aerosols from African solid fuel smoldering combustion in the humid (∼65% RH) chamber experiments, exhibits a strong fuel-type dependency (Figure 1). BB aerosol emissions derived from hardwoods (acacia, eucalyptus, mopane, mosetlha, mukusi, wanza, and wild olive) are more absorptive (0.8–1.6 m^2^ g^–1^ at 370 nm; 0.2–0.5 m^2^ g^–1^ at 470 nm) than BB aerosol emissions from mokala branches, mopane leaves, cow dung, and savanna grass (0.2–0.7 m^2^ g^–1^ at 370 nm; 0.1–0.2 m^2^ g^–1^ at 470 nm), as shown in Table S1. The MAC_OA,λ_ range of the African hardwood-derived BB aerosol emissions is comparable to, or lower than, the methanol-extractable primary particulate BB-OA MAC_λ_ reported for ambient samples from Switzerland (1.5–2.3 m^2^ g^–1^ at 370 nm; 0.4–0.6 m^2^ g^–1^ at 470 nm; Figure 1). The latter represents hardwoods burning in residential stoves at European rural sites (e.g., beech, oak, and birch) and is comparable to previously reported conventional primary BB-OAs from lab experiments and observations in different atmospheric environments.^41^ These values^41^ were constrained within a narrow range by applying source apportionment on UV–vis absorbance measurements^31^ combined with Mie calculations at AE33 wavelengths,^41^ which is a fundamentally different approach than that applied here (Section 2.2.).

#### 3.1.2 Absorption Emission Factors

The humid-chamber OA absorption emission factor spectra (AEF_OA,λ_) were estimated by multiplying MAC_OA,λ_ (in m^2^ g^–1^) by the PM mass-based EF (EF_PM_; Text S2), since the smoldering combustion particles are organic-rich. AEF_OA_ values at 370 nm range from 5 (leaves) to 37 (mukusi) m^2^ kg^–1^ of fuel burned (Table S1), with an average of 19 m^2^ kg^–1^. These values are similar to the 25–35 m^2^ kg^–1^ range reported by Selimovic et al. for lab-simulated Western US wildfire-derived BrC measured at 401 nm with an MCE < 0.9.^42^ Furthermore, Martinsson et al. burned logs of birch and reported AEF values for BrC in the range of 19–24 m^2^ kg^–1^ at 370 nm.^43^ Tian et al. reported BrC AEF values measured at 370 nm relevant to China, ranging from 15–47 m^2^ kg^–1^ for crop residues.^44^ Notably, the AEF_370 nm_ value for both the African savanna grass (Table S1) and South Asian grass^45^ is ∼9–10 m^2^ kg^–1^. Our AEFs decreased by a factor of 5 at 470 nm and further at 660 nm to an average of 1.7 m^2^ kg^–1^, with a range from 0.7 (leaves) to 3.2 (wanza) m^2^ kg^–1^ (Table S1).

#### 3.1.3 Impact of Humidity and UV Exposure on MAC

**Figure 2:**
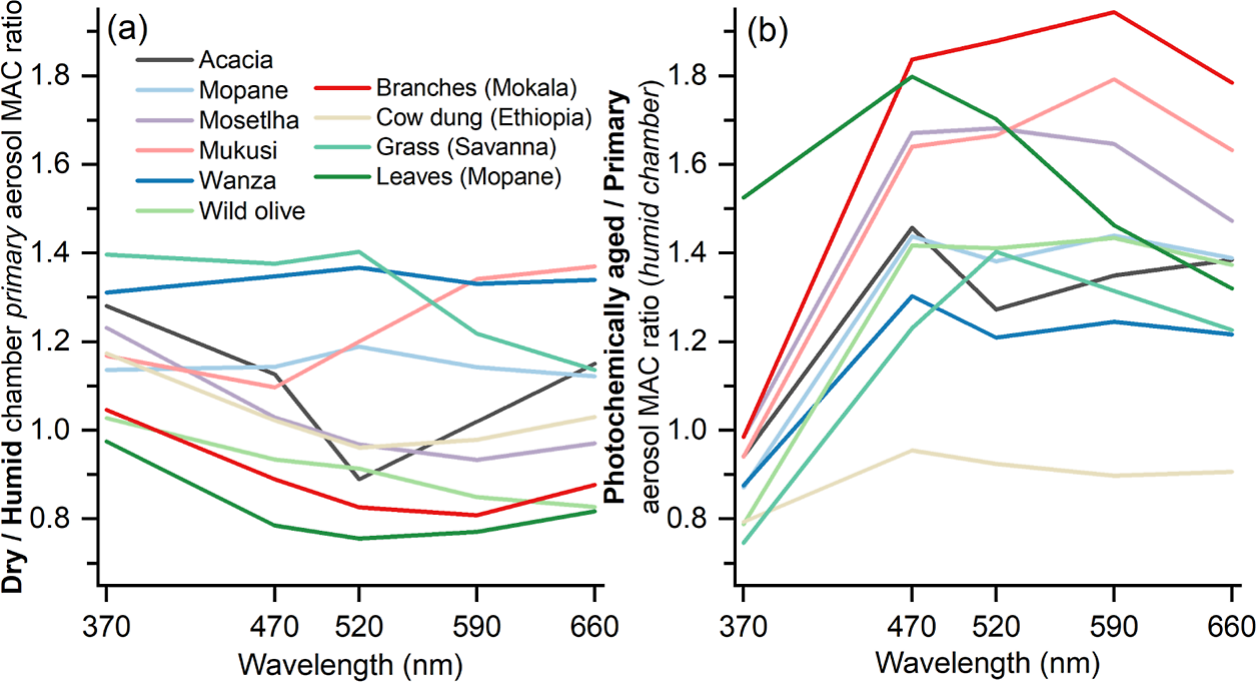
Impact of environmental conditions, simulated during smog chamber experiments, on fuel-specific MAC_λ_. The two conditions evaluated are (a) primary emissions in the dry versus prehumidified chamber and (b) photochemical aging of primary emissions in the humid chamber. Eucalyptus is not featured here, because only humid-chamber primary emissions were sampled for this fuel.

In our chamber experiments, the MAC spectra were influenced not only by the biomass fuel type (Figure 1) but also by humidity and UV exposure (Figure 2). In primary emissions, MAC_370 nm_ values were up to 1.4 times higher under dry chamber conditions (<10% RH) compared to those in a prehumidified (∼65% RH) chamber (Figure 2a), with no evident wavelength dependence. Interestingly, this trend was not observed for leaves, grass, and mokala branches, which had similar MAC_370 nm_ values at ∼65% RH and <10% RH. Perhaps this could be due to differences in hygroscopicity or phase state. Section 4 discusses the uncertain role of chamber wall losses (at varying RH conditions) of gases or particles that might contribute to BrC. Conversely, Figure 2b highlights that when primary emissions are aged via UV exposure in the ∼65% RH chamber, there is a reduction in aerosol MAC by up to 25% at 370 nm (again, leaves are an exception). Yet, MAC showed an average increase of 1.5 times in the 470–660 nm range, with cow dung as an exception. UV light affects MAC in two main ways: (1) By altering aerosol composition which influences absorption, either through UV photobleaching or UV-driven gas-phase oxidation or multiphase chemistry; 2) By changing the aerosol mass concentration.^33,46−48^ The change in MAC may occur due to secondary organic aerosol (SOA) formation via gas-phase chemistry, in the presence of emissions like organics, NO_*x*_, and HONO. Both photobleaching and formation of purely scattering SOA can decrease the MAC (see also Sections 3.2.2 and 4).

#### 3.1.4 Classification into BrC Optical Classes

**Figure 3:**
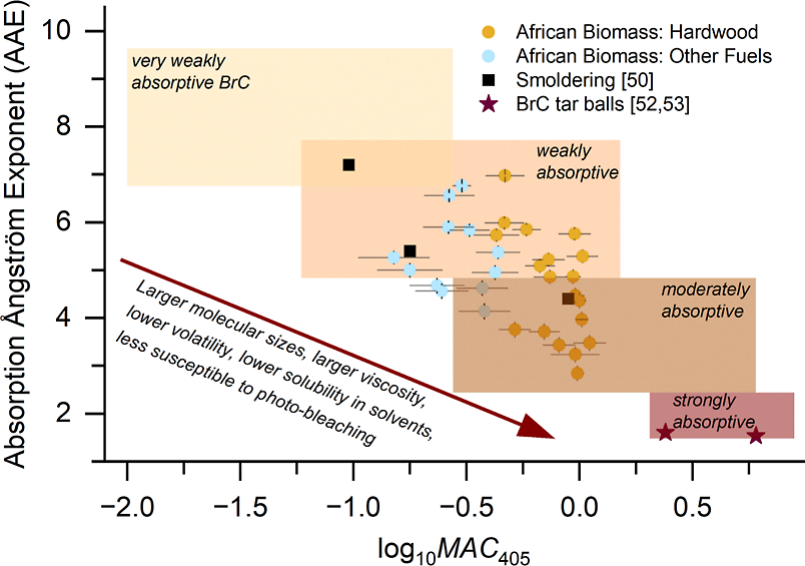
Optical absorption properties of primary and aged OAs (in the humid chamber) derived from African biomass smoldering combustion, plotted in the AAE – log_10_MAC_405_ space, building upon Saleh^49^ and extended to shorter wavelengths by Hettiyadura et al.^35^ The AAE values were calculated over the wavelength range of 370–470 nm for this study and from 350 nm up to 1000 nm in the literature. Different BrC classes varying in absorptivity from very weak (top left) to strong (bottom right) are represented by shaded areas. Circles display the optical absorption properties of samples from this study, derived from calibrated aethalometer measurements. Error bars represent the uncertainty based on four repeated chamber experiments (including different fuel types and all aging conditions) and the aethalometer calibration uncertainty combined in quadrature. Literature values from BB smoldering burns^50^ are represented by squares, and the star symbols correspond to individual “tar-ball particles” as inferred from electron energy loss spectro-microscopy.^52,53^

Figure 3 shows that the primary and aged African BB (organic-rich) aerosols in the humid chamber are weakly to moderately absorptive, which is typical of smoldering-dominated combustion, and they are largely methanol-soluble (methanol extracts accounted for 85–90% of total organic carbon mass; Text S2). Classification into BrC optical classes^49^ is based on the short-wavelength absorptivity (MAC_405 nm_) and its wavelength dependence as expressed by the AAE.^35^ The AAE values of 3–7 at 370–470 nm fall within the range defined by previous smoldering biomass combustion experiments (black rectangles; AAEs at 405–532 nm), which included pine needles and Siberian/Indonesian peat as well as featured in situ photoacoustic measurements with optical closure (see Siemens et al.^50^ and references therein).

### 3.2 Quantification of BrC Aerosol Mass

The RPLC/DAD-ESI-HR-QTOFMS/MS method (Section 2.4) effectively identified the majority of RPLC-separated BrC species in ambient samples and the chamber-aerosol samples derived from smoldering-dominated combustion, including BrC standards (Figures S2 and S3 and Table S2). Of the 60 BrC standards used for quantification (Table S2), 24 (40%) were ionized in both (−)ESI and (+)ESI, 18 (30%) were exclusive to (−)ESI (including nitroaromatic compounds, NACs), and 18 (30%) were ionized solely in (+)ESI. ESI ionization efficiency varies substantially between compounds due to their distinct sizes and functional groups.^51^

We divided the RF of each BrC standard by the population geometric mean per ion mode to obtain relative response factors (RRFs; Figure S4). The 2 orders-of-magnitude variability in RRFs underlines the necessity of using multiple standards spanning a wide range of chemical classes, structures/polarities, molecular weights, and retention times to effectively quantify BrC species.

**Figure 4:**
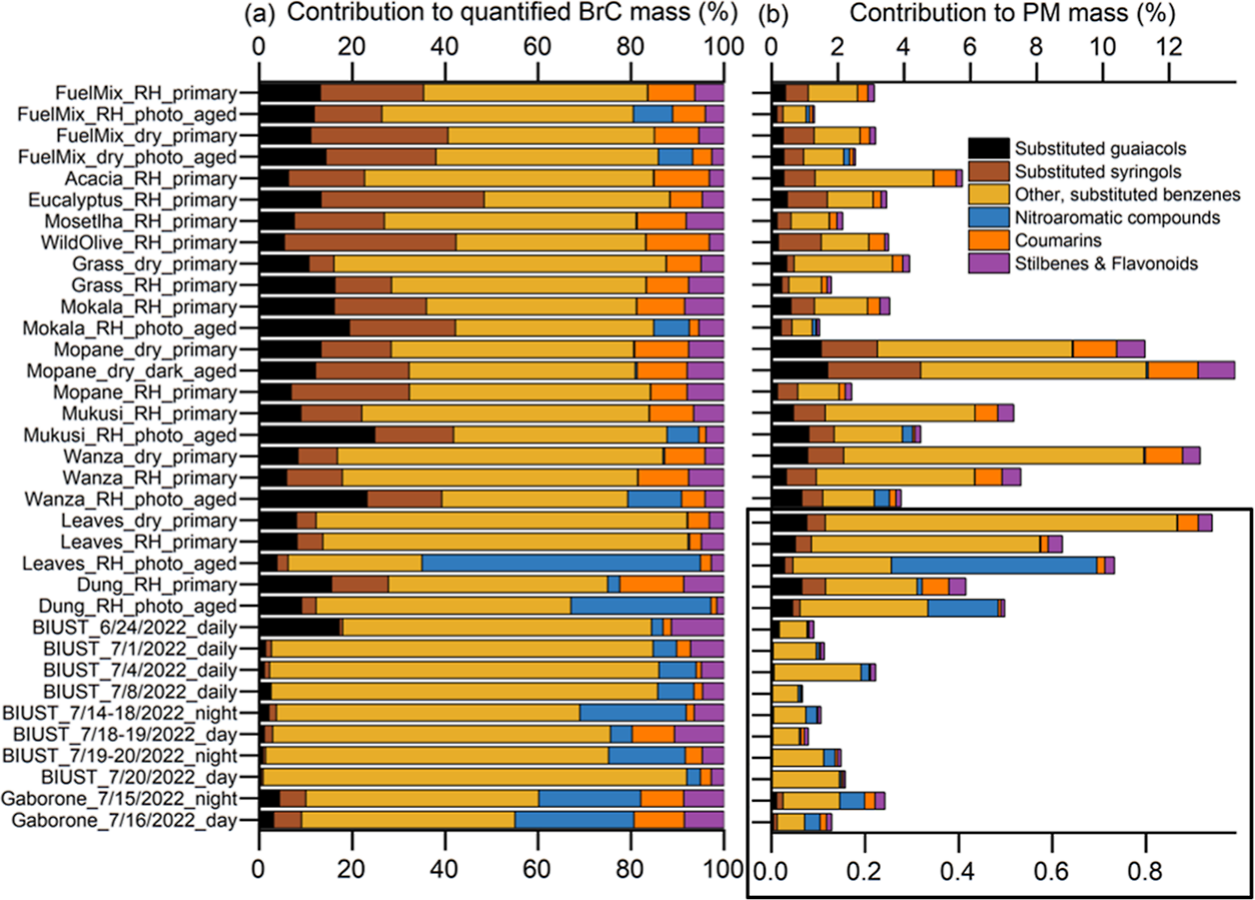
Mass closure of all 182 BrC species identified through RPLC/DAD-ESI-HR-QTOFMS/MS using the RFs from Table S2 (RRFs shown in Figure S4): (a) percentage contribution of different BrC chemical classes to the total quantified RPLC/ESI-HR-QTOFMS/MS-based BrC mass in chamber- and ambient-aerosol filter samples, displayed as cumulative contributions, and (b) percentage contributions (cumulative) of each chemical class to PM mass across samples—the highlighted lower subpanel corresponds to a distinct scale. The fuel mixture contained equal masses of all fuels, excluding cow dung, to mimic a wildfire scenario with numerous biomass types. Refer to Figure S5 for the relationship between Figure 4b and OA absorptivity (Figure 1) and Figure S7 for the clustering of samples/compounds based on individual BrC species’ fractional contributions to BrC mass.

Figure 4 shows recovery-corrected mass contributions of 182 identified BrC species (Text S3, S4 and Table S4), determined using the RFs of the 60 standard compounds (Table S2), grouped into chemical classes. Shown are results from the ambient-aerosol samples from Botswana, primary aerosol emissions from all fuel types in the humid chamber, selected dry chamber dark, and selected photochemically aged chamber samples; chamber-aerosol samples were selected based on the aethalometer data and trends (Figure 2 and Text S3). While only 25% of the total identified BrC species had a matching authentic standard, these species accounted for the majority of the total identified BrC aerosol mass (mass fraction *Q*_1_–*Q*_3_ among samples: 0.52–0.64; *Q*: quartile).

#### 3.2.1 Linking BrC Aerosol Mass to OA Absorption

A strong linear relationship (Pearson’s *r*: 0.85) exists between the fraction of the (OA-rich) PM that is BrC (i.e., the sum of identified BrC species mass) and the MAC at the near-UV wavelength. The linear-fit (Figure S5a) slope of 0.14 ± 0.02 m^2^ per g of total quantified BrC directly links BrC aerosol mass to aerosol light-absorption cross section and, thus, can be used to parametrize BrC in models. The correlation remains robust (Pearson’s *r*: 0.89) even when only the mass of the BrC species with matching authentic standards (∼60% of total quantified BrC mass; Section 3.2) is considered for the *x*-axis values, suggesting that the quantification approach with surrogate standards does not influence the results. However, a perfect linear relationship is generally not expected, as individual chromophoric species with distinct MAC spectra contribute variably to BrC/aerosol mass across samples. The effect of sample-to-sample variability in chemical composition on this relationship is highlighted by the higher linear-fit slope at 470 nm (Figure S5b) for photochemically aged BB emissions (0.10 ± 0.03; Pearson’s *r*: 0.78) as compared to primary (dark-chamber) and dark-aged emissions (0.03 ± 0.01; Pearson’s *r*: 0.81). This difference is attributed to the formation of visible-light-absorbing NACs (Figure S3) upon irradiation of the primary/dark-chamber emissions (Section 3.2.2 and Figure 2b).^54^

#### 3.2.2 Mass Closure of Identified BrC Species Grouped into Chemical Classes

While lignin pyrolysis products are considered major contributors to BrC aerosols due to the prevalence of lignin in biomass, their contribution to BrC mass and variations due to photochemical aging have not previously been characterized, to our knowledge.^55^Figure 4a shows that in the humid-chamber BB aerosol samples, the *Q*_1_–*Q*_3_ fractional contributions (first and third quartiles) to total BrC mass (identified through RPLC/DAD-ESI-HR-QTOFMS/MS; Section 2.4) by combined substituted guaiacols, substituted syringols, and other substituted benzenes (i.e., lignin pyrolysis products) as well as coumarins and (stilbenes + flavonoids) are 0.08–0.15, 0.10–0.23, 0.45–0.62, 0.04–0.11, and 0.04–0.08, respectively. While lignin pyrolysis products dominate the chamber BrC aerosol mass, NACs contribute significantly to photochemically aged BrC aerosols, accounting for up to 12% in hardwood-derived BB emissions and 30–60% for dung and leaves. In the ambient Botswana PM samples, “other substituted benzenes” are the most abundant among potential lignin pyrolysis products, whereas higher molecular-weight BrC species (mainly coumarins and flavonoids) collectively contribute up to 20% of BrC mass, similar to chamber aerosols. NACs contribute up to 8 and 23% of BrC mass in daytime and nighttime BIUST samples, respectively, and 22–26% in Gaborone samples. Daytime–nighttime sample comparisons suggest that ambient NACs might form in the dark, prompting further research into the simulated nighttime processing of African-BB primary BrC emissions (e.g., with nitrate radicals,^56^ or in clouds/wet aerosols^57^) and their daytime evolution.

Figure 4b shows that primary/dark-generated BrC aerosols from the smoldering combustion of African hardwoods (acacia, eucalyptus, mopane, mosetlha, mukusi, wanza, and wild olive) in the humid chamber typically exhibit a higher relative abundance in fine PM mass (OA-rich; 2.0–14%) as compared to emissions from the combustion of cow dung, mokala branches, mopane leaves, and savanna grass (0.4–3.6%). Similar to MAC (Figure 2 and Section 3.1.3), this abundance is influenced by chamber RH and UV exposure. Primary BB emissions in the dry chamber contain 1.5 to 4.5 times more BrC (in % of PM mass) compared to emissions from replicated experiments in the humid chamber. On the other hand, photochemical aging of primary emissions in the humid chamber generally reduces the relative abundance of BrC by up to 60%, except for leaves and dung where the formation of NACs compensates for other BrC species losses (Figure 4b). Section 4 further discusses these trends.

The relatively lower abundance of combined BrC species in ambient PM (up to 0.2%) might be linked to a range of factors, including a lower relative abundance of OA in PM compared to chamber samples (which affects gas-to-particle partitioning of semivolatile organics); BrC whitening due to complex aging mechanisms in the atmosphere (e.g., photobleaching and dilution-driven evaporation; Text S2); influence of other fuel types or sources with lower BrC content (Text S2), such as hydrocarbon-like (nonaromatic) emissions; high combustion efficiency; lower solubility of ambient BrC in methanol compared to chamber emissions; and the presence of coarse-mode aerosol species (e.g., dust).

#### 3.2.3 BrC vs PM EFs

The PM EF (EF_PM_; Text S2) for smoldering combustion from African BB in the humid chamber is 20 ± 5 g per kg of fuel burned, consistent with our previous findings^38^ and largely independent of fuel type (Table S1). This range is similar to reported values for Western US wildfires (26 g PM_1_ kg^–1^)^58^ and smoldering savanna and grassland fire smoke (∼10–25 g PM_1_ kg^–1^),^6^ as well as traditional Tibetan biomass stoves (25 g kg^–1^) and Ugandan 3-stone fires (15 g kg^–1^),^59^ but higher than South Asian biomass stoves (7 g kg^–1^).^59^ In contrast, combined BrC species EFs derived from African fuels in the humid chamber show greater variability and depend on the fuel type, ranging from <0.15 g BrC per kg for cow dung and mopane leaves to ∼1.7 g kg^–1^ for mukusi (Table S1). Sun et al. reported a geometric mean EF_BrC_ of 0.7 g kg^–1^ for Chinese household biomass fuels (straw, stalk, pine, and pellets) under mixed-combustion conditions.^60^

Figure S6 shows that the major humid-chamber primary BrC aerosol species with a matching authentic standard are sinapaldehyde (#86, Table S2), 1-phenyl-1,3-butanedione (#64, Table S2), and nodakenetin (#102, Table S2), with EFs up to ∼200 mg kg^–1^. These are followed, in descending order of the 11-fuel mean EF, by 3-hydroxybenzoic acid (#35), syringic acid (#46), coniferaldehyde (#85), acetosyringone (#78), syringaldehyde (#69), 3,4-dihydroxybenzaldehyde (#18), homovanillic acid (#47), pyrogallol (#3), 4-methylcatechol (#61), scopoletin (#73), vanillic acid (#36), and vanillin (#60). Note that species-specific EFs are given by the mass fraction of each BrC species in the total filter-collected primary aerosol mass (Text S4), multiplied by the EF_PM_. Chemical structures, along with other major species quantified with surrogates, correspond to the IDs shown in Table S2. The wide range of lower-to-upper percentiles emphasizes the substantial fuel-to-fuel variability in BrC species EFs, with the lowest values generally reported for dung and leaves. Studies are warranted to determine additional fuel-specific EFs, particularly for individual BrC species.^61^

### 3.3 Detailed BrC Molecular-Level Composition

**Figure 5:**
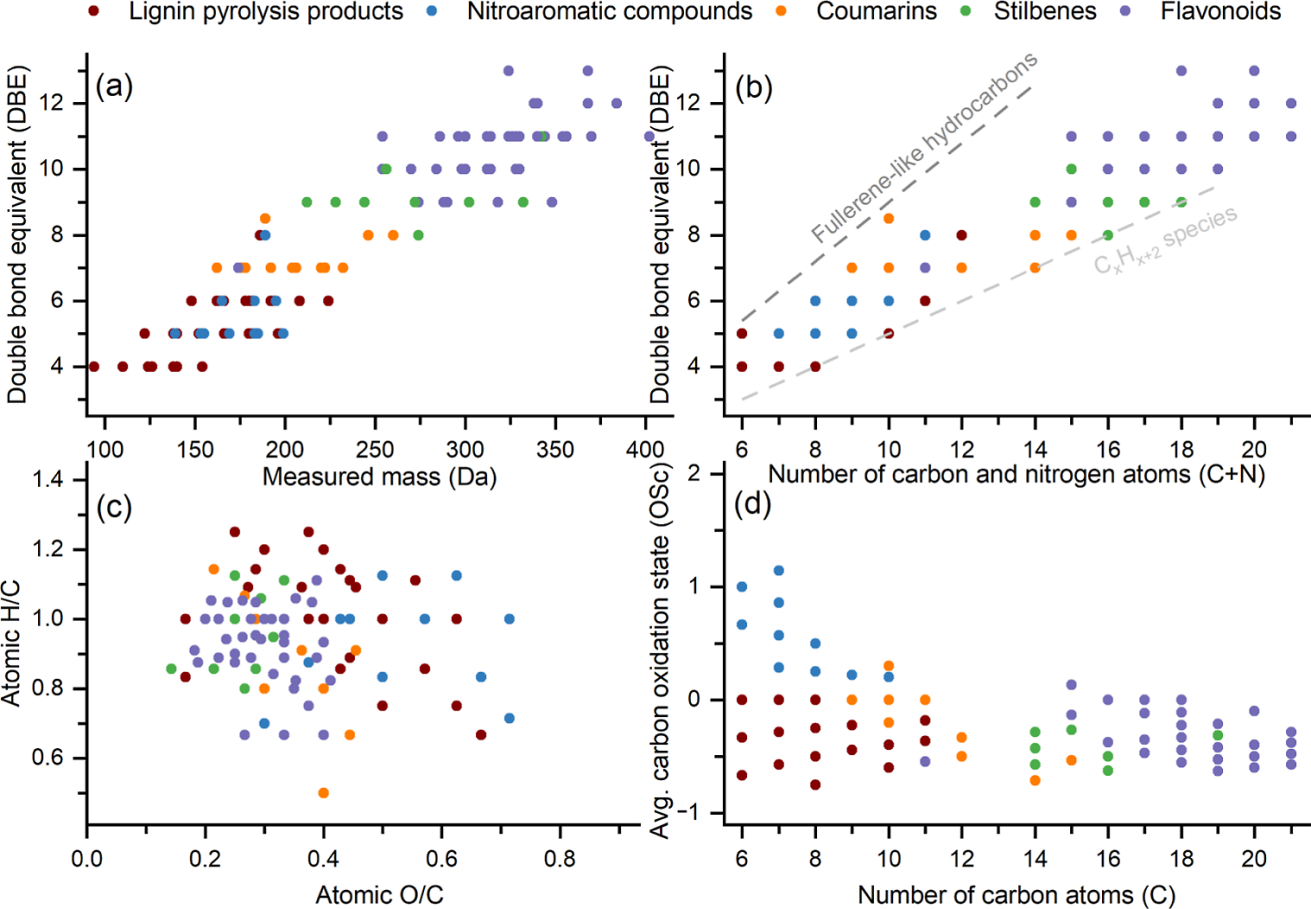
Molecular-level characteristics of all 182 identified BrC species that are shown in Table S4 (i.e., DAD-absorbing formulas identified with ESI), including authentic standards (Table S2). These are categorized by chemical class (lignin pyrolysis products include substituted guaiacols/syringols among other substituted benzenes) and display: DBE [DBE = C – (H/2) + (N/2) + 1] as a function of (a) measured (neutral) mass or (b) carbon plus nitrogen atoms, (c) van Krevelen diagram displaying atomic O/C vs H/C ratios, and (d) average oxidation state of carbon [OS_C_ = (2 × O–H + 3 × N)/C] as a function of carbon-atom count. Note that some data points from the same or different chemical classes overlap. Panel (b) demonstrates that all RPLC/DAD-ESI-HR-QTOFMS/MS-based absorbing formulas fall within the BrC-relevant space,^30,50^ with DBE/(C + N) ratios ranging from 0.5 to 0.9, as indicated by the dashed lines.

Figure 5 illustrates molecular-level characteristics of all 182 identified BrC species (Table S4).^29^ The double bond equivalent (DBE) represents the total number of rings and π bonds and increases from 4 to 6 for lignin pyrolysis products to ∼7–8, 9, and 10–12 for coumarins, stilbenes, and flavonoids, respectively (Figure 5a). All identified chromophoric species fall within the “BrC-relevant space”,^30,50^ characterized by DBE/(C + N) ratios of 0.5–0.9 (Figure 5b). This indicates some degree of conjugation across their molecular structures,^30,50^ which seems to be a crucial requirement for light absorption. The van Krevelen plot (Figure 5c) and carbon oxidation state (Figure 5d) indicate moderate oxygenation and functionalization of the identified BrC species, consistent with primary and moderately aged BB-OAs.^62,63^ The species-mass-weighted atomic ratios (O/C, H/C) of the BrC aerosol population in (humid/dry) chamber dark/primary, (humid/dry) chamber photochemically aged, and ambient filter samples are (0.35 ± 0.01, 0.99 ± 0.04), (0.44 ± 0.04, 0.94 ± 0.07), and (0.46 ± 0.03, 0.88 ± 0.08), respectively (± indicates the 1σ variability between samples).

We employed hierarchical clustering (Section 2.6 and Text S6) to simplify the complex BrC molecular-level composition matrix. Through this clustering (see dendrogram in Figure S7), we discerned several distinct feature types and compared them with those found in ambient PM samples. BrC species introduced below for the first time will be specified by their formula and ID from Table S4, and tentative structures quantified using surrogate standards will be shown in *italics*. Primary BrC aerosol species from burning savanna grass, leaves, and cow dung are discussed in Text S7 as these samples were less absorptive than hardwood-derived emissions (Figure 1).

#### 3.3.1 Hardwood-Derived Primary BrC Species

In chamber-aerosol samples, coniferaldehyde (C_10_H_10_O_3_, #85) and sinapaldehyde (C_11_H_12_O_4_, #86) are major chromophoric species, contributing ∼0.6% and 0.8–1.2%, respectively, of the primary PM mass from wanza and mopane combustion in the dry chamber. The coniferaldehyde/sinapaldehyde (C/S) mass ratio in primary smoldering combustion emissions has a *Q*_1_–*Q*_3_ range of 0.18–0.43. Photochemical aging in the humid chamber reduces their abundance by 80–90% (in line with Fleming et al.^48^) and 95%, respectively; the C/S mass ratio increases >2.0 in nonhardwood-derived photochemically aged emissions.

Hardwood-derived emissions exhibit up to 0.6, 0.1, and 0.1% contribution to fine PM mass from vanillic acid (C_8_H_8_O_4_, #36), its likely structural/positional isomer (RT = 12.4 min, *#31*), and hydroxybenzoic acids (C_7_H_6_O_3_, *#21* and *#35*), respectively. All chamber aerosols, except for those from cow-dung/leaf burning, contain syringic acid (C_9_H_10_O_5_, #46), which contributes a maximum of 0.4% to PM mass. The most abundant coumarin and substituted benzene detected in our study are nodakenetin (C_14_H_14_O_4_, #102) and 1-phenyl-1,3-butanedione (C_10_H_10_O_2_, #64), respectively. They represent 0.6–1.2 and 0.8–1.8%, respectively, of PM mass in primary BB emissions from mukusi, acacia, wanza, and mopane. Both compounds are highly sensitive, with 70% of nodakenetin lost upon photochemical aging (corroborating Fleming et al.^48^); 1-phenyl-1,3-butanedione vanished upon irradiation, likely due to a photochemical reaction that occurs when the molecule absorbs light energy, causing the bond adjacent to the carbonyl group to break (Norrish type I cleavage^64^).

Furthermore, acetosyringone (C_10_H_12_O_4_, #78) is present in all chamber-aerosol samples, contributing up to 0.3% to PM mass. *Catechol–resorcinol* isomers (C_6_H_6_O_2_, #5 and #11) and 3,4-dihydroxybenzaldehyde (C_7_H_6_O_3_, #18) are more abundant in primary emissions from mopane, mukusi, and wanza combustion, particularly in the dry chamber, making up 0.6–1 and 0.2% of PM mass. Homovanillic acid (C_9_H_10_O_4_, #47) contributes 0.05–0.2% to PM in hardwood-derived primary emissions. However, homovanillic acid and its likely positional isomer (RT = 14.8 min, *#50*) are susceptible to photochemical aging. Primary emissions of *cinnamic acid* (C_9_H_8_O_2_, #55) and *methoxycinnamic acid* (C_10_H_10_O_3_, #70) are strongly correlated with homovanillic acid, but the latter is 3 times less abundant. *Dihydroxy-cinnamic acid* (C_9_H_8_O_4_, #51) is present in primary BB aerosol emissions from mopane, wanza, and grass in the dry chamber; however, its relative abundance decreases by 60–90% in the humid chamber. *Hydroxy-dimethoxychalcone* (C_17_H_16_O_4_, #172) contributes ∼0.05–0.1% to PM mass in mopane-, mukusi-, and wanza-derived primary BrC aerosols.

#### 3.3.2 Tracer BrC Species for African Hardwoods

We identified several primary BrC species that serve as tracers for specific hardwood species (Figure S7) within the range of African biomass fuels included in this study. Pyrogallol (C_6_H_6_O_3_, #3) serves as a tracer for primary emissions from wanza combustion in the dry (1.9% of PM mass) or humid chamber (0.7% of PM mass). Scopoletin (C_10_H_8_O_4_, #73), a methoxy-hydroxycoumarin, is a tracer for primary emissions from wild olive smoldering combustion (0.3% of PM mass), along with *hydroxytyrosol* (C_8_H_10_O_3_, #10; 0.5% of PM mass), tyrosol (C_8_H_10_O_2_, #24), esculetin (C_9_H_6_O_4_, #38), *(hydroxy-)dimethoxycoumarin* isomers (C_11_H_10_O_4–5_, #79 and #96), and *hydroxy-methoxychalcone* (C_16_H_14_O_3_, #118). However, we found that scopoletin is prone to photochemical aging and could not detect it in ambient PM samples from Botswana. On the other hand, *dihydroxy-dimethoxyflavone* (C_17_H_14_O_6_, #154), previously identified as a potential tracer for ceanothus burning,^48^ is only detected in ambient PM samples from BIUST and Gaborone. Another species native to Africa, not among those in the literature study, must be producing dihydroxy-dimethoxyflavone upon combustion since ceanothus has a North American range. *Hydroxy-trimethoxychalcone* isomers (C_18_H_18_O_5_, nos. 170 and #176) act as tracers for wanza, mopane, and mukusi primary emissions. *Coniferyl ferulate or tetrahydroxyflavanone* (C_20_H_20_O_6_, #147) and *hexamethoxyflavone* (C_21_H_22_O_8_, #150) contribute to primary BrC aerosols emitted from burning mosetlha and eucalyptus, respectively.

#### 3.3.3 Evolution upon Simulated Photochemical Aging

The formation of NACs is observed during photochemical aging in the humid chamber, potentially mediated by HONO that has been photolyzed by UV light. Abundant NACs, listed in the order of increasing RT, include 4-nitrocatechol (#66) as well as its positional isomers (*#8*; C_6_H_5_NO_4_), 5-nitrosalicylic acid (C_7_H_5_NO_5_, #80), 4-nitrophenol (#87) as well as its positional isomers (C_6_H_5_NO_3_, *#2* and *#12*), *methoxy-nitrocatechol* (C_7_H_7_NO_5_, #91), *methoxy-nitrophenols* and *methyl-nitrocatechols* (#19, #92, and #100) such as 4-nitroguaiacol (#95) and 2-methyl-4-nitroresorcinol (C_7_H_7_NO_4_, #123), dimethoxy-nitrophenol (C_8_H_9_NO_5_, e.g., nitrosyringol, #99), *dimethyl-nitrobenzoic acids* (C_9_H_9_NO_4_, #106, #116, and #120), *nitroacetophenone* or *methyl-nitrobenzaldehyde* (C_8_H_7_NO_3_, #110), *methyl-nitrophenols* (#42 and #44) including 4-nitro-o-cresol (C_7_H_7_NO_3_, #117), *dimethoxy-nitrobenzene* (C_8_H_9_NO_4_, #128), and 4-nitro-1-naphthol (C_10_H_7_NO_3_, #145). The most abundant NAC is 4-nitrocatechol, which may be a product of the OH-initiated reaction of catechol, accounting for 0.3–0.4% of PM mass in photochemically aged emissions from mukusi and wanza. The detected NACs correspond with previous findings in chamber/ambient aerosols or cloud-water samples associated with BB or mixed-source urban emissions.^28,29,65−69^

Furthermore, syringaldehyde (C_9_H_10_O_4_, #69) contributes up to 0.4% of PM mass in photochemically aged emissions from mopane, mukusi, and wanza. Vanillin (C_8_H_8_O_3_, #60) forms upon photochemical aging of mukusi and wanza primary BB emissions in the humid chamber (∼0.6% of PM mass), suggesting that it may be an oxidation product of lignin formed from the breakdown of larger aromatic compounds present in primary BB emissions.^70^*Methoxy-catechol* (C_7_H_8_O_3_, #57) forms upon photochemical aging of hardwood-derived BB emissions. *Coumarin-carboxylate* (C_10_H_5_O_4_, #67) exhibits similar behavior and also appears in photochemically aged emissions from leaf burning. Finally, acetovanillone (C_9_H_10_O_3_, #72) contributes ∼0.03% to PM mass in photochemically aged emissions from mukusi and wanza.

#### 3.3.4 BrC Species in Ambient Aerosols and Similarities to Chamber Aerosols

While photochemically aged chamber-aerosol samples cluster together (Figure S7), the composition of aged BrC from leaf and cow-dung burning exhibits unique features, placing them in the same subcluster with ambient-aerosol filter samples (Figure S7). For instance, increased levels of compounds like 4-nitrocatechol, 4-nitrophenol, 4-nitro-o-cresol, *methoxy-nitrocatechol*, 4-nitroguaiacol and its positional isomers, the flavonoid kaempferol (C_15_H_10_O_6_, #125), and *coumarin-carboxylate* are found in both the ambient aerosols and photochemically aged emissions from leaf burning. On the other hand, *methylcatechol* (C_7_H_8_O_2_, #37), benzoic (C_7_H_6_O_2_, #81) and phthalic (C_8_H_6_O_4_, #20) acids, (di)hydroxy-benzaldehydes (#18, *#21*, and *#43*), and *methoxy-nitrocatechol* are found in both the ambient aerosols and photochemically aged emissions from cow-dung combustion.

Additional BrC species that are common between ambient- and primary/aged chamber-aerosol samples include the following: 4-nitro-1-naphthol, *nitrosyringol*, *dimethoxy-nitrobenzene* or *methoxy-methyl-nitrophenol*, and *methoxy-nitrophenols* or *hydroxy-methyl-nitrophenols*, which are present in photochemically aged chamber-aerosol samples; terephthalic (C_8_H_6_O_4_, #25) and phthalic acids, which contribute up to 0.03% and 0.1% of PM mass, respectively, collected in BIUST and Gaborone and show levels comparable to those in photochemically aged chamber-aerosol samples from mukusi and wanza; *p*-coumaric acid isomers (*#28* and *#41*) that contribute significantly to BrC from hardwood and grass burning (Text S7); and *methoxy/dihydroxybenzoic acids* (C_7_H_6_O_4_, #9 and #82), *hydroxybenzoic acid* or *dihydroxybenzaldehyde* (#21), *hydroxy-benzaldehyde* (C_7_H_6_O_2_, #26), vanillin, (methyl-)umbelliferone (C_9_H_6_O_3_, #71 and C_10_H_8_O_3_, #88), 3,4-dimethoxybenzoic acid (C_9_H_10_O_4_, #76), *dihydroxyflavone* (C_15_H_10_O_4_, #152), and *dihydroxyflavanone* or a *stilbenoid* (C_15_H_12_O_4_, #155). Other contributing species to BrC mass in the ambient-aerosol samples include *hydroxyphthalic acid* (C_8_H_6_O_5_, #17), *dihydroxy-dimethoxyflavone*, as well as a *phenanthrenoid*, *chalcone*, or *flavanone* (C_15_H_12_O_4_, #173).

## 4 Atmospheric Implications

The impact of African BB aerosols on regional climate and intercontinental air mass transport is significant.^71,72^ Our research offers a comprehensive quantification of optical absorption and molecular characteristics of African BB-derived aerosols and sheds light on the implications of their atmospheric processing. By systematically analyzing biomass fuels from this understudied region,^73^ we have advanced the fundamental understanding of the atmospheric chemistry of BrC aerosols.^74^ We quantified a large number (182) of highly absorptive (in the near-UV region) methanol-extractable organic compounds in chamber smoldering burns of African biomass. This detailed molecular analysis highlights the key role of light-absorbing moieties, present in trace concentrations (Figures 4 and S7), in driving particle-phase OA absorptivity. While individual BrC species contribute <1–2% of fine PM mass, in combination, they account for over 10% of PM mass in some African fuels examined during our laboratory studies. Besides identifying major contributing compounds, their classes, and their EFs, we have pinpointed specific BrC tracer species that can distinguish between different African biomass fuels, aiding in source apportionment studies. Moreover, our source/aging experiments have practical implications for interpreting ambient air measurements. Notably, the photochemically aged BrC emissions from leaf and cow-dung combustion exhibit molecular fingerprints similar to those found in ambient PM collected from Botswana (Figure S7), offering a direct link to real-world conditions.

Our findings suggest a complex role of the phase state and photochemistry in influencing the BrC composition and OA absorptivity. At ∼ 65% chamber RH, we observed a reduction in the relative abundance of primary BrC mass in PM (Figure 4b), which in turn reduces the OA absorptivity at 370 nm (Figure 2a). Yet, changes in the molecular-level BrC composition with varying RH are likely not significant (Figure S7), and the fractional contributions from different BrC chemical classes remained consistent in different states (Figure 4a). At elevated RH, the water absorbed by particles may reduce their viscosity, potentially promoting the diffusion of water-soluble BrC species into the gas phase. Furthermore, the presence of moisture on the chamber walls might cause a higher tendency for species to adhere to the wall compared to the dry chamber condition. The latter could lead to potential selective losses of BrC aerosol species or gases^75,76^ to the chamber walls, affecting the intensive optical absorption characteristics of primary BB aerosols. With photochemical aging, the OA MAC reduction at 370 nm and enhancement at longer wavelengths (Figure 2b) relate to the photochemical degradation of lignin pyrolysis products and concurrent formation of visible-light-absorbing NACs (Figures 4 and S3).

Current air quality and climate models tend to underestimate the impacts of BrC due to limited data. Our findings are crucial for representing BrC aerosols in atmospheric models and parametrizing their optical properties (Section 3.1 and 3.2.1), toward simulating their radiative forcing with greater accuracy. The detailed characterization of BrC species from diverse biomass fuels aids source apportionment efforts in Africa, where BB is a major aerosol source, and existing emission inventories often rely on data from North American and European contexts. We provide necessary data to update these inventories,^77^ enabling more accurate environmental impact assessments of African BB-derived BrC emissions. This research can also guide the development of integrated climate change mitigation and air quality management policies^78,79^ to minimize the impact of BB on human health and climate, particularly in sub-Saharan Africa.

## Data Availability

Raw data that support the findings of this study are available from the corresponding authors upon reasonable request. Data presented in figures^80^ are publicly available in Zenodo (DOI: 10.5281/zenodo.10601481).

## Supporting Information Available

Information on the African biomass fuels, chamber experiments and measurements, filter sample collection, sample preparation for BrC molecular analysis, BrC species quantification approach (including standard preparation and analysis and recovery analysis), justification for and limitations of the molecular-level analysis methodology, hierarchical clustering, and BrC molecular-level composition in nonhardwood-derived emissions; information on the chamber- and ambient-aerosol samples that were used for analyzing the molecular composition of BrC, authentic standards used to quantify BrC species detected in aerosol filter samples, fragmentation information for BrC standards, and the 182 separated BrC species identified in chamber- and ambient-aerosol filter samples; and geographical distributions of African hardwood biomass fuels, extracted wavelength chromatogram of aerosol filter sample extracts, mass absorption coefficient spectra of BrC standards and their RRFs, scatter plots of BrC mass closure result and OA absorptivity, BrC species’ EFs in the humid chamber, and hierarchical clustering of BrC species’ fractional contributions to BrC mass in 35 aerosol filter extracts (PDF)

## Notes

^▽^ Megan M. McRee was formerly known as Megan Mouton.

## Acronyms

AAE: absorption Ångström exponent
AEF: absorption emission factor
AE33: aethalometer model 33
ATN: attenuation
BB: biomass burning
BrC: brown carbon
DAD: diode array detection
EF: emission factor (mass-based)
ESI: electrospray ionization
HR-QTOFMS/MS: high-resolution quadrupole time-of-flight tandem mass spectrometry
MAC: mass-absorption cross-section
MCE: modified combustion efficiency
MWAA: multiwavelength absorption analyzer
NACs: nitroaromatic compounds
OAs: organic aerosols
PM: particulate matter
RH: relative humidity
RPLC: reversed-phase liquid chromatography
(R)RF: (relative) response factor
RT: retention time
