# Supplementary material for: Quantifying the Light-Absorption Properties and Molecular Composition of Brown Carbon Aerosol from Sub-Saharan African Biomass Combustion
Source: Environ Sci Technol. 2024 Feb 23;58(9):4268–80. doi: 10.1021/acs.est.3c09378 (PMC10919089; doi:10.1021/acs.est.3c09378)
Supplement: Supplementary file 1 — es3c09378_si_001.pdf [file es3c09378_si_001.pdf]

Supporting Information for

Quantifying the Light-Absorption Properties and Molecular Composition of Brown Carbon Aerosol from Sub-Saharan African Biomass Combustion

Vaios Moschos[†‡*], Cade Christensen[§], Megan Mouton[⊥], Marc N. Fiddler[ǁ], Tommaso Isolabella[♯¶], Federico Mazzei[♯¶], Dario Massabò[♯¶], Barbara J. Turpin[‡], Solomon Bililign[†⊥*], and Jason D. Surratt[‡§*]

[†] Department of Physics, College of Science and Technology, North Carolina A&T State University, Greensboro, North Carolina 27411, United States

[‡] Department of Environmental Sciences and Engineering, Gillings School of Global Public Health, The University of North Carolina at Chapel Hill, Chapel Hill, North Carolina 27516, United States

[§] Department of Chemistry, College of Arts and Sciences, The University of North Carolina at Chapel Hill, Chapel Hill, North Carolina 27599, United States

[⊥] Department of Applied Sciences and Technology, College of Science and Technology, North Carolina A&T State University, Greensboro, North Carolina 27411, United States

[ǁ] Department of Chemistry, College of Science and Technology, North Carolina A&T State University, Greensboro, North Carolina 27411, United States

[♯] Department of Physics, University of Genoa, 16146 Genoa, Italy

[¶] National Institute for Nuclear Physics (INFN), University of Genoa, 16146 Genoa, Italy

* Corresponding Authors:

Vaios Moschos; moschosv@yahoo.com, vmoschos@ncat.edu

Solomon Bililign; bililign@ncat.edu

Jason D. Surratt; surratt@unc.edu

The SI includes:

Supporting Text

Tables S1 to S4

Figures S1 to S7

SI References

Number of pages: 32

Supporting Text

Text S1. African Biomass Fuels[1-3]

Acacia abyssinica,[4] also known as Ethiopian acacia, flat-top acacia, or red thorn, is a tree species belonging to the *Fabaceae* family. It is native to East-South Africa (Fig. S1), particularly in countries such as Ethiopia, Somalia, Kenya, Tanzania, Uganda, Zambia, Zimbabwe, Malawi, Mozambique, and South Africa. It typically grows in savannas, grasslands, and woodland ecosystems at altitudes ranging from sea level up to 2,400 meters, and is well-adapted to regions with semi-arid to sub-humid climates. *Acacia abyssinica* wood is known for its durability and resistance to termites, making it suitable for various applications, including construction, fence posts, and furniture. The wood is known to produce good quality firewood due to its relatively high calorific value (high amount of energy per volume), which means it generates a good amount of heat when burned. Moreover, it tends to burn slowly, thus providing sustained heat for an extended period. It is a relatively common tree in its native regions, making it readily available as a source of firewood.

Acacia erioloba,[5] commonly known as Mokala, camelthorn, or giraffe thorn, is a tree species belonging to the *Fabaceae* family. It is native to the arid regions of Southern Africa, primarily found in countries such as Botswana, Namibia, South Africa, and parts of Zimbabwe (Fig. S1). Mokala wood is dense, hard, resistant to termites, and has a high calorific value giving it similar properties and uses as *acacia abyssinica*.

Baikiaea plurijuga,[6,7] commonly known as Mukusi, Rhodesian teak, or African teak, is a tree species belonging to the *Fabaceae* family. It is native to the Southern African regions, particularly in countries such as Angola, Botswana, Namibia, Zambia, and Zimbabwe (Fig. S1). The tree is typically found in dry deciduous forests, especially on Kalahari sands, and plays a vital role in the ecology of these areas. Mukusi wood is highly valued for its strength, durability, and resistance to termites and fungal decay. It is used for various purposes, such as construction, flooring, furniture, and even railway sleepers. Although not a primary source of fuel, the wood from Mukusi trees can be used as firewood or for charcoal production. The dense and hard nature of the wood allows it to produce long-lasting, hot fires, making it a suitable fuel source.

Colophospermum mopane,[8,9] commonly known as the Mopane tree, mopani, butterfly or balsam tree, is a species of tree belonging to the *Fabaceae* family. It is native to Southern Africa, mainly found in countries such as Angola, Botswana, Malawi, Mozambique, Namibia, South Africa, Zambia, and Zimbabwe (Fig. S1). The tree typically grows in hot, dry regions, often dominating the woodland vegetation in these areas. Mopane wood is dense, heavy, and durable, making it suitable for various uses, including construction, furniture, and fencing. When it comes to using Mopane as a fuel, its properties allow it to burn slowly and produce intense heat, making it an excellent choice for firewood, particularly for low and slow indirect (traditional) cooking, high-heat direct cooking, and charcoal production. In regions where Mopane trees are abundant, local communities rely on them for cooking and heating purposes. Mopane trees are also host to *Gonimbrasia belina* or Mopane worms, which primarily feed on mopane leaves and are an important source of protein.

Cordia africana,[10,11] also known as the African Cordia, Wanza, or African cherry, is a tree species that belongs to the *Boraginaceae* family. It is native to various regions in Africa, particularly in the eastern and southern parts, including Ethiopia, Kenya, Tanzania, Malawi, Mozambique, Zambia, and Zimbabwe, and can also be found in South Sudan and Uganda (Fig. S1). The tree is often found in montane forests, wooded grasslands, and riverine areas, and it is well-adapted to a range of environmental conditions. It is valued for its many uses, such as timber, fodder, medicine, and ornamental purposes. As a fuel source, *cordia africana* has a moderate calorific value and can be used for firewood/charcoal production. The wood is known for its hard and durable properties, making it a good choice for producing heat/energy. In rural areas of Africa, firewood and charcoal from *cordia africana* are used for cooking/heating.

Eucalyptus camaldulensis,[12,13] commonly known as the River Red Gum or Bahir Zaf in Amharic, is a tree species belonging to the *Myrtaceae* family. It is native to Australia but has been introduced to many other parts of the world, including Africa, Asia, Europe, and the Americas. In its native range, *Eucalyptus camaldulensis* typically grows along watercourses, floodplains, and wetlands, as it has a high tolerance for waterlogged soils. *Eucalyptus camaldulensis* wood is known for its durability and resistance to termites, making it suitable for various applications such as construction, fence posts, and furniture. The wood is relatively dense and has a high calorific value, meaning it generates a good amount of heat when burned. It also burns relatively slowly, providing sustained heat for an extended period. In some regions, *eucalyptus* species are grown and managed as a source of firewood and biomass energy.

Olea europaea,[14,15] commonly known as the European or wild olive, is a tree species belonging to the *Oleaceae* family. The native range of *Olea europaea* mainly includes the Mediterranean Basin, encompassing countries in Southern Europe, Western Asia, and Northern Africa; it can also be found in East-South Africa in forests, woodlands, and scrub areas (Fig. S1). The wood of the wild olive tree is dense, hard, and heavy, making it suitable for various applications, such as woodworking, carving, and furniture production. Olive wood has a high calorific value, meaning it generates a good amount of heat when burned. It also burns relatively slowly and produces a pleasant aroma, which makes it an attractive choice for firewood. However, due to the value of the olive tree for its fruit and oil, it is not typically grown or managed primarily for firewood production. The use of *olea europaea* as firewood is usually limited to prunings or trees that are no longer productive.

Peltophorum africanum,[16,17] commonly known as Mosetlha, African wattle, or Weeping wattle, is a tree species belonging to the *Fabaceae* family. It is native to Southern Africa and can be found in countries such as Angola, Botswana, Eswatini, Mozambique, Namibia, South Africa, Zambia, and Zimbabwe (Fig. S1). The tree typically grows in bushveld, woodland, and savanna areas, as well as along riverbanks and in rocky terrains. The wood of Mosetlha is moderately dense, and its durability makes it suitable for various applications like construction, furniture, and fence posts. When it comes to using Mosetlha as a fuel, the wood can be used for firewood/charcoal production. In regions where Mosetlha trees are abundant, local communities may rely on them for cooking/heating.

Traditional fuel sources in sub-Saharan Africa:[18,19] In addition to wood, other traditional fuel sources include cow-dung and tree branches or leaves. Cow-dung, formed into cakes or patties after drying, is an economical, readily available, and renewable resource, particularly in rural areas where cattle farming is common. It produces a moderate amount of heat suitable for daily household needs. Tree branches and leaves, common fuel sources in many African regions, have been traditionally used for cooking, heating, and boiling water. While leaves burn faster and generate less heat compared to branches or logs, they remain a significant energy source.

Drivers of burnt area in sub-Saharan Africa:[20] The savanna biome is characterized by a mix of grasslands and scattered trees, covering large parts of the continent, particularly in East, West, and Southern Africa. During wildfires, various types of fuels can be burned, depending on the vegetation and materials present in the affected area. Wildfires often consume trees, shrubs, and other woody plants, including their branches, leaves, and duff. In grassland or savanna ecosystems, grasses can serve as a primary fuel source for wildfires. Fires can spread quickly through dry grasses and other ground-level vegetation, contributing to the intensity and speed of the wildfire.

The hardwood species discussed earlier can be burned during wildfires if they are present in the affected area. While these hardwood trees may be more resistant to fire due to their denser wood and higher moisture content, they can still catch fire under certain conditions, especially during intense wildfires. The branches and leaves of these hardwood species can also burn, particularly in forested areas or regions with dense vegetation, contributing to the overall fuel load and intensity of the fire.

Text S2. Chamber Experiments, Measurements, and Filter Sample Collection

Chamber Experiments. The 9-m^3 fixed-volume smog chamber, operated in batch mode and made of fluorinated ethylene propylene film, is housed in an air-conditioned room with controlled lighting at North Carolina A&T State University (NC A&T) located in Greensboro, NC.[21] The room features a heated flask for introducing humidity to simulate sub-tropical African conditions and nighttime relative humidity (RH) levels. The RH was either 0-10% for dry chamber experiments, or elevated to 65-70% before each humid chamber experiment. We ensured all combusted fuels were dried (e.g., by placing them under a fume hood), and the resulting moisture content was typically <5% (Moisture Analyzer PCE-MA 50X). Furthermore, hardwood samples were debarked to maintain a uniform fuel composition[22] and ensure repeatability of the burn experiments. Each fuel was weighed pre-combustion at room conditions (Table S1). Despite our primary focus on examining fuel-to-fuel variations in aerosol chemical emissions, burning a mixture of fuels may better reflect regional wildfire emissions. Combustion emissions were transferred from the tube furnace (directly, without any dilution step) into a cyclone (URG, URG-2000-30ENS-1) with a 2.5-μm cut point and then into the Teflon chamber, where they were thoroughly mixed, with the aid of an electric blower, with particle-free zero-air in approximately 30 min (confirmed by achieving stable SMPS distributions for primary emissions).[21]

The mass-based emission factor (EF_{PM}), which is the mass concentration (*M*; see next paragraph) of primary BB aerosol into the chamber times the chamber volume (9 m^3) divided by the mass of fuel burned, typically ranged between 15-25 g of PM kg^{-1} (Table S1); primary-aerosol *M* was determined after mixing and before aerosol filter sampling/measurements. The chamber houses light fixtures; UV lamps (36 in [length], F30T8/350BL/ECO, Sylvania)[21] were turned on during photochemical aging. With the UV lamps active, the chamber temperature increased up to 8 °C above room temperature, and RH dropped to 30-40%. Each burn experiment lasted around 6-7 h. This duration includes the time taken to increase the RH before combustion (up to 1 h) and the time allocated for sampling and measurements. The procedure for cleaning the chamber between experiments was outlined in Smith et al.[21] Briefly, the chamber was flushed with an amount of zero-air equivalent to four times its volume. During this process, the UV lamps were turned on to guarantee a thorough cleaning. The effectiveness of this chamber cleaning was verified by the SMPS-determined aerosol *M* returning to background levels (i.e., ~1-2 μg m^{-3}) before beginning the next chamber experiment. However, it remains possible that semi-volatile compounds could off-gas from the chamber walls and re-partition into the gas phase. Nonetheless, the organic gas background remained below an average of 10% of primary combustion gaseous organic emissions (determined from measuring total organic carbon in a mist chamber sample).

Measurements. The aethalometer-based mean $b_{atn,AE33,\lambda}$ (Eq. 1) was calculated by examining the stability of 1-min values throughout the measurement period at each condition; we only considered *ATN* values <40, because for these values the dual-spot automatic loading-correction was reliable (based on our test measurements/calculations). Consequently, due to the prevalence of high-*M* light-absorbing organics from smoldering-dominated burns, we often used the data corresponding to spot 2 (lower flow rate of 1.2 L min^{-1}) for shorter wavelengths with high $\Delta ATN/\Delta t$. We used the SMPS (TSI 3080 DMA and 3787 WCPC), an aerosol particle mass analyzer (APM, Kanomax 3602), a cavity ring-down optical spectrometer, and a nephelometer (TSI 3563) to measure the physical and optical properties of the BB-derived aerosols. In a subsequent publication, we will detail these findings, including measurements from gas-phase chemical monitors (NO_x, O_3, CO, and CO_2). The APM was deployed to estimate the particle effective density, which consistently measured 1.2 ± 0.1 g cm^{-3} regardless of fuel type, photochemical versus dark conditions, and particle size for smoldering fires generating OA-rich spherical particles.[23] The estimated filter-collected chamber-aerosol PM mass (Table S1) is actually ~$PM_{0.72}$ because the upper limit of the SMPS used in this work was 720 nm; nevertheless, an insignificant fraction of particles measured were >700 nm.[22] We found that the equivalent black carbon (eBC) from aethalometer measurements and elemental carbon (EC) from Sunset Analyzer measurements of selected filter samples collected from the smoldering fires were both at background levels and not significantly different from zero. We then used the SMPS- and APM-based mass concentration (*M*) to derive emission factors and MAC spectra of BrC-containing OA-rich aerosols. We note that the calculated MAC (Eq. 2 in main text) might be slightly underestimated due to the presence of inorganic aerosol components in these emissions, as suggested by previous bulk chemical composition measurements,[22] yet the associated bias likely remains within the calculated uncertainty (~30%). Therefore, the reported results can be regarded as the effective MAC of non-refractory, organic-rich particulate matter. Finally, Sunset thermal-optical carbon analyzer test measurements of untreated versus methanol-extracted chamber-aerosol quartz fiber filters (4 selected primary and photochemically aged samples, including fuel-mix burn emissions) suggested the OA was primarily methanol-soluble; specifically, the methanol extract accounted for 85-90% of the total OC mass.

We note that high mass concentrations (*M*) of primary aerosols in the chamber of typically 800 μg m^{-3} were necessary for conducting the campaign, due to the experiment duration (considering particle wall losses) and aerosol sampling/measurement detection limits. This high aerosol *M* may correspond to localized and intense BB events in the atmosphere, such as large wildfires or agricultural burns.[24] During such events, high emission rates can lead to elevated concentrations of aerosols in the immediate vicinity of the source.[25] In the real atmosphere, outside of intense BB events, mass concentrations of BB-OA are typically lower, with background levels that vary[26,27] but are often in the range of a few μg m^{-3}. These lower concentrations are due to dilution, dispersion, and removal processes that occur as the aerosols disperse over larger areas and mix with the surrounding air.[28-30] In the presence of high aerosol mass concentrations, the partitioning of semi-volatile compounds to the aerosol phase is favored (Pankow theory[31]). Therefore, if there exist high OA mass concentrations, one would expect a higher proportion of semi-volatile organic compounds to be found in the particle phase rather than the gas phase.

Ambient Aerosol Filter Sampling in Botswana and Filter Samples from Chamber. We performed ambient aerosol sampling using a Savillex 401-21-47-30-21-2 filter holder combined with pumps. Sampling durations were approximately 24 h or 12 h, with daytime intervals from 09:00 to 17:30 and nighttime from 18:00 to 08:30. The calculated volumetric flow rate was approximately 23 L min^{-1} at BIUST using a duel-piston reciprocating pump (Rocker 800), and 17 L min^{-1} in Gaborone (at Airport Junction Shopping Centre, and A1) with a Leland Legacy portable pump. To estimate PM mass, we weighed the filters pre- and post-sampling. The mean RH and temperature during the sampling campaign were ~50% and 21 ºC, respectively. It is worth mentioning that the aerosol samples were obtained during a short research visit to BIUST. The influence of transported emissions from BB, whether from controlled burns or wildfires, in addition to emissions from local residential biomass combustion, on the sampling sites during this period remains uncertain. Future targeted field studies in Africa are crucial to investigate BB-derived BrC aerosol tracers.

Collected filter samples included laboratory blanks (taken directly from the filter package), field blanks (obtained by placing the filter in the holder for a few minutes without turning on the pump), and chamber blanks (collected with the pump on before the start of the combustion, when the SMPS mass was <0.5% of the primary combustion aerosol levels). We note that Teflon filters are expected to adsorb little or no gas-phase organic carbon in comparison to QFFs. Each chamber- and ambient-aerosol filter sample, as well as chamber-/field-blanks (Text S2), was placed in a labeled petri dish (MilliporeSigma PD1504700), packaged in aluminum foil, and stored in plastic bags at -20 °C. The ambient samples were transported immediately after the field campaign to NC A&T (upon remaining for a day at ambient temperature). Teflon filters were transported to the UNC Chapel Hill atmospheric multiphase chemistry laboratory in a cool box with ice packs for molecular-level characterization of BrC.

Text S3. Chamber & Ambient Aerosol Sample Preparation and BrC Molecular Analysis

Coupling DAD analysis with RPLC/ESI-HR-QTOFMS detection is key to distinguishing individual chromophoric species from non-absorbing constituents that typically make up the majority of methanol-extractable organic aerosol. We selected filter samples from the combustion experiments (carried out in Summer 2022), prioritizing primary emissions from each fuel in the humid chamber, and certain dry-chamber primary or photochemically aged aerosol samples based on the aethalometer results and trends (Figs. 1-2 and Table S1). Selected filter samples included chamber- and field-blanks (Text S2). Aerosol samples aged in the dark were generally not preferred for molecular-level analysis: this is due to the nature of this chamber condition serving as a control for all other conditions examined (e.g., varying RH and dark vs. photochemical conditions), where no oxidants were introduced into the dark chamber. If aerosols remained in the chamber under dark conditions for extended time periods, this would mainly cause physical aging of the particles within the accumulation mode as suggested by SMPS data, and the MAC remained unchanged, indicating a chemical composition similar to initial primary emissions injected into the dark chamber. Building on previous methods,[32,33] we developed sample preparation and measurement protocols for analyzing the chemically complex African BB-derived OA.

The sample preparation for the molecular-level chemical analysis was carried out in Fall 2022. Teflon filter punchouts, with the polypropylene support ring and ink labeling removed, were placed in pre-cleaned scintillation vials with polytetrafluoroethylene (PTFE) caps. The samples were extracted once in 20 mL of methanol (Optima grade, Fisher) and then sonicated for 45 minutes. To prevent thermal decomposition of analytes, the water inside the sonicator was drained midway through the process. Each extract was then filtered through a pre-conditioned 0.2-µm PTFE membrane syringe filter to remove insoluble matter and split into four equal fractions. We employed glass syringes, metal needles, and LC/MS-certified polyethersulfone (PES) syringe filters during sample preparation to minimize contamination. Blank filters confirmed these components used for extraction did not introduce unwanted chemical contamination to aerosol filter samples.

Three of the four fractions were concentrated at room temperature using a high-purity N_2 evaporator. Each extract was reduced to approximately 150 µL, transferred to a 250 µL glass vial insert, and further dried to 35 µL. To reduce evaporative losses of OA constituents, the drying process was deliberately left incomplete. The final volume (*V*) was adjusted to 70 µL by adding 35 µL of deionized (DI) water, yielding a 50/50 methanol/water mixture needed for RPLC/DAD-ESI-HR-QTOFMS analyses. The extracted material remained dissolved throughout the reconstitution process. The remaining ¼ of each extract underwent gas chromatography interfaced to mass spectrometry analysis for source-diagnostic species including sugars (detailed results to be presented separately in a forthcoming study).

An Agilent 1200 Series LC system with a DAD (10-mm path length) coupled to an ESI-HR-QTOFMS 6520 series instrument was used to perform the molecular-level chemical analysis of the final, concentrated filter extracts immediately after the sample preparation. We injected 5 µL of the concentrated filter extracts (and solvent blanks) and obtained DAD signals from 200-800 nm. The chromatographic separations were conducted using a Waters ACQUITY UPLC HSS T3 C18 column (2.1×100 mm, 1.7 µm particle size, Waters Corporation, Milford, MA) at 45 °C. The 60-minute gradient elution scheme (0.2 mL min^{-1} flow rate) was identical for (+)/(–)ESI, with the only difference being the mobile phase additive. The RPLC/(–)ESI-HR-QTOFMS/MS used DI water with 0.05% acetic acid (Optima grade) as mobile phase A, and acetonitrile (Optima grade) with 0.05% acetic acid (Optima grade) as mobile phase B. RPLC/(+)ESI-HR-QTOFMS/MS used DI water with 0.1% formic acid (Optima grade) as mobile phase A, and acetonitrile with 0.1% formic acid (Optima grade) as mobile phase B. The gradient began with 5% B for 3.5 minutes, linearly increasing to 60% B between 3.5-45 min. The linear gradient then increased from 60-100% B between 45-52 minutes. A hold at 100% B was maintained until 55 minutes before decreasing back to 5% B at 56 min and holding until 60 min. A rinse protocol was performed after each sample measurement to ensure low background between injections. The LC-measured retention time (RT) of species ionizing in both modes remained consistent, except for phthalic acid with RT of 10.4 and 12.3 min in (–)ESI and (+)ESI, respectively. This difference is attributed to ion pairing due to distinct mobile phase additives. The ESI source for both ion modes operated at 300 °C with a gas flow of 10 L min^{-1} and a nebulizer pressure of 35 psig. The voltage cap was 3500 V with a fragmentor voltage of 130 V, skimmer voltage of 65 V, and an octopole RF peak of 750 V. Tandem MS analysis was performed with 3 precursors selected for fragmentation during the MS^1 cycle. The collision-induced dissociation (CID) voltage was calculated using the linear formula: $V = 5 \cdot [(m/z)/100] + 15$. Mass spectral data was averaged for 1 s during collection of MS^1 scans (~13,000 transient spectra) and 0.5 s during collection of MS^2 scans (~6,500 transient spectra). Data was collected for multiple scans over the mass-to-charge (*m/z*) ratio range of 60-1,000 for MS^1 and 40-700 for MS^2. Real-time mass correction was performed by continuous nebulization of a mixture of purine, leucine enkephalin, and hexakis(1H,1H,3H-tetrafluoropropoxy)phopsphazene. These provided reference mass ions in both (–)ESI and (+)ESI ions. The entire molecular-level data collection was carried out within a week to minimize instrument drift and response variability.

Data were processed using Agilent MassHunter Workstation Qualitative Analysis software (Version 10.0 Build 10.0.10305.0, Agilent, CA, USA). Upon MS background subtraction, possible formulas for each separated ion were calculated with an *m/z* ratio tolerance of 0.005 and limited by elements ($C_{5-25}H_{2-25}O_{1-15}N_{0-2}$) as well as the nitrogen rule. Neutral formulas were determined by examining ions with the potential addition of H^+, Na^+, K^+, or NH_4^+ in (+)ESI, and removal of H, or addition of $HCOO^-$ or CH_3COO^- in (–)ESI. BrC species were identified by aligning a delay time of 0.15 min between the passage of chromatographic peak through the DAD and MS detectors. The peak list was exported and BrC neutral formulas were identified by comparative analysis to the DAD-chromatogram peak retention times (LC-RTs) in all analyzed samples. Tentative structures were compared for plausibility between the two ion modes. BrC species were not detected by the DAD during analysis of chamber or field blank filter sample extracts (Text S2), emphasizing that all BrC species analyzed during this experiment were a direct result of collection from biomass combustion (for chamber aerosols) or ambient air (for Botswana samples). We also note that there existed several RPLC/DAD-ESI-HR-QTOFMS/MS-detected formulas within the BrC-relevant space (Fig. 5) that did not correspond to a short-wavelength DAD peak; these non-absorbing BB-derived OA constituents (not included in Fig. 5) will be a focus of future studies.

Text S4. Details on the BrC Species Quantification Approach

Standard Preparation and Analysis. The standards were prepared in methanol as stock solutions at 100 ppm for each species with 25 species in each group. These were sonicated for 4 hours to ensure complete dissolution of the solids, resulting in a total dissolved BrC concentration of ~2.5 mg mL^{-1} in each stock solution. No precipitate formation was observed after sonication, confirming effective extraction of the standards. The four distinct groups of standards did not contain isomers (that our analytical platform can measure) and were analyzed with the analytical platform (Sect. 2.4, Text S3) at 10 ppm to obtain their signatures (LC-RT, measured mass, absorbance spectrum, fragmentation pattern).

Mass absorption coefficient spectra in methanol solution, corresponding to the mass-normalized light absorption by each BrC standard in the four 10-ppm groups, were calculated from the DAD data sets according to the following equation[34] and the extracted blank-subtracted UV-vis absorbance spectra: $MAC_{\lambda,i} = \frac{A_{\lambda,i} \cdot F \cdot \ln 10}{l \cdot m_{inj} \cdot 60 \cdot 0.01}$, where $A_{\lambda,i}$ (in mAU * s) represents the area of the RPLC-DAD chromatogram recorded at wavelength λ over the RT period when a BrC species is separated, F denotes the RPLC flow rate (0.2 mL min^{-1}), and l signifies the path length of the DAD optical cell (1.0 cm). The injected mass (m_{inj}, in ng) of each standard is known based on the weighed mass added in the stock solution, the dilution factor (x10 dilution, from 100 ppm to 10 ppm), and the injected volume (5 µL). The coefficients *ln10*, 60, and 0.01 account for the conversion of solution to cross-sectional absorption, min into s, and the combined conversion of mAU into AU, cm^3 (mL) into m^3, cm into m, and ng into g, respectively. Normalized mass absorption coefficient spectra were successfully compared to available reference spectra (found online on SpectraBase[35]) reported under neutral conditions in methanol.

Recovery Analysis. For the recovery analysis, 5 blank Teflon filters were spiked with a 2.5 ppm BrC standard mixture (prepared from a stock 25 ppm mixture) and then extracted using methanol. The sample extraction and preparation process mirrored the one used for aerosol filter samples. The extraction resulted in a 0.5 ppm BrC standard mixture after reconstitution. The efficiency of recovery was determined by comparing the species-specific EIC peak areas to 5 replicates of the same 0.5 ppm BrC standard mixture measured directly, bypassing the laboratory extraction, sonication, and drying steps. The recovery uncertainty (Table S2) was based on the variance between the replicates of the spiked filters and the external mixtures. For a majority of the species, the recovery stood at approximately 66%. However, lower recovery rates were noted for certain low molecular weight substituted benzenes (benzoic acid, 4-methylcatechol, pyrogallol, and 5-hydroxymethylfurfural; Table S2), the stilbenes (with a class mean of 13%), and specific flavonoids (with a class mean of 44%) – yielding a weighted mean of 38% for stilbenes/flavonoids. The 1σ recovery variability, detailed in Table S2, was significantly influenced by the N_2-drying phase during the sample preparation. To elucidate further, the first of the five spiked filter extracts (no.1) was reduced to around 150 µL before being transferred to a 250 µL glass vial insert, and subsequently reduced further to 35 µL. These volumes match the ones used for the aerosol filter samples (see Text S3). Conversely, the second and third spiked filter extracts (no. 2 & 3) were initially dried to about 200 µL (rather than the aforementioned 150 µL), while the last two (no. 4 & 5) were brought down to roughly 50 µL. The maximum and minimum species-specific recoveries were noted for samples no. 2 & 3 and no. 4 & 5, respectively. As such, the blow-down method does affect the recovery efficiency, with larger values corresponding to larger volumes transferred to the vial insert; blowing down to dryness is therefore not recommended for quantitative analysis. The mean recovery derived from all 5 spiked filters aligned closely with that of no. 1.

Our BrC species quantification incorporated the following steps:

1. Identify authentic standard species that match RTs in the samples (i.e., chromophoric species with identical molecular formulas, based on MassHunter analysis, UV-vis spectra, and tandem MS (when available for both standard mixtures and aerosol extracts).
2. Establish a quantification mode based on detection sensitivity, upper and lower limits of linear detection, and comparison to ranges in aerosol filter samples.
3. Quantify the mass of an authentic BrC standard i, m_i (in µg), with an EIC peak area A_i, in a final extract of volume V = 70 µL (or 0.07 mL), as follows: $m_i = (A_i / \mathrm{RF}_i) \cdot V$. The response factor (RF) of authentic standards is reported in units of EIC peak area per concentration in µg mL^{-1}. Note that m_i corresponds to three-quarters of the original filter extracts (Text S3); therefore, for mass closure calculations (Fig. 4) we considered three-quarters of the total PM mass collected on each filter (Table S1). Refer to step (10) below for recovery corrections.
4. Assign chemical formulas (based on MassHunter analysis) to remaining chromophoric species in aerosol sample DAD extracted wavelength chromatograms, for BrC species without a matching authentic standard (Table S4 and Fig. S2).
5. Use structural similarity and/or adjacent RTs to assign surrogate standards for quantifying BrC species without authentic standards.
6. Quantify the mass of each chromophoric species without a matching authentic standard using the RF of a single standard if the assigned molecular formula, tentative aromatic structure (assisted by ChemSpider), DAD absorbance spectrum, and RTs are similar (implying comparable structure, polarity, and physical/chemical properties).
7. When conditions in (5)-(6) are not met, consider multiple surrogates and calculate their mean RF for quantification of that chromophoric species. Employing multiple surrogates can enhance the resulting accuracy and benefit from a combination of surrogates accounting for different compound properties.
8. Indicate the formula and RT combinations for species quantified using authentic vs. surrogate standards (Table S4).
9. For less-characterized higher-molecular weight chromophoric species (stilbenes and flavonoids) with challenging tentative aromatic structure assignment (even with ChemSpider assistance of known compounds), use the chemical family mean RF for quantification.
10. Correct m_i for species-specific recovery; similar to RFs, apply a mean recovery for species quantified with surrogates. For species ionizing in both modes, the recovery of the authentic-standard quantification mode was applied, as both mode values were similar.

We note that the RF of authentic standards within associated candidate chemical families (i.e., stilbenes and flavonoids with C>15, molecular mass>250 Da, and RT>25 min) exhibited less variability between individual compounds. Conversely, for lower-molecular weight authentic standards belonging to other chemical classes (lignin pyrolysis products, coumarins, nitroaromatic compounds), substantial variability in the RFs was observed between the chemical classes (Fig. S4), and between lignin pyrolysis products sub-classes (substituted guaiacols, substituted syringols, and other substituted benzenes). In the latter case, emphasis was placed on structural similarity and/or adjacent RTs when assigning (multiple) surrogates to BrC species without a matching authentic standard. We note that the ionization mode does not affect the linear relationship between MAC and mass closure result (Sect. 3.2.1): For BrC species that ionized in both ion modes, the scatter plot (for all 35 aerosol filter extracts) of the combined BrC species fractional contribution to PM mass in (+)ESI (x-axis) vs. (–)ESI (y-axis) showed a strong (Pearson's r: 0.96) linear relationship (slope: 0.71).

Contrary to expectations,[36] the measured positional isomers exhibited different RTs (Table S2), likely due to varying polarity.[36] These include terephthalic acid (11.3 min) vs. phthalic acid (10.4 min), umbelliferone (17.8 min) vs. 4-hydroxycoumarin (21.6 min), 4-

methylumbelliferone (21.5 min) vs. 4-hydroxy-7-methylcoumarin (26.2 min), and the dihydroxy-coumarins esculetin (13.1 min) vs. daphnetin (14.5 min). For these pairs, the RF was within a factor of 2 (Table S2; the RF of 4-hydroxy-7-methylcoumarin and daphnetin in (+)ESI was $1.84 \cdot 10^6$ and $1.27 \cdot 10^5$, respectively, but these were not used for quantifications). This underscores the importance of step (7) in conjunction with (5) and (6) as mentioned earlier.

Light-/Air-Sensitivity of BrC Standards. We confirmed the light-sensitivity, stated in their MSDS, of the following BrC standards, based on their reduced/low relative abundance to PM mass in the aerosol filter samples upon photochemical aging: 5-hydroxymethylfurfural, 6,2′,4′-trimethoxyflavone, 4-nitrophenol, coniferaldehyde, sinapaldehyde, and 3'-hydroxypterostilbene. On the other hand, compounds such as 3,4-dihydroxybenzaldehyde, syringaldehyde, coniferaldehyde, and sinapaldehyde that are expected to be light/air-sensitive could be detected in the aerosol samples. However, other air-sensitive standard compounds that could not be detected with our analytical platform include 2,3-dihydroxybenzaldehyde, 2′-hydroxypropiophenone (liquid), 4-acetoxybenzaldehyde (liquid), guaiacylacetone, 4-methoxybenzaldehyde (liquid), furaneol, and 3,4-dimethoxybenzaldehyde (or veratraldehyde). Further, the following species were also present in the mixture of 100 BrC standards, but could not be detected with our analytical platform, or were not identified in aerosol filter samples and also not used as surrogates for quantifications (Table S2): hydroquinone, 4-methoxyphenol, 2-nitrophenol, coumarin, 3,5-dimethoxybenzaldehyde, ethyl salicylate, 6-methoxy-2-naphthoic acid, 2′,4′-dimethoxyacetophenone, 2′-methoxyacetophenone, 7-ethoxy-4-methylcoumarin, ethyl coumarin-3-carboxylate, 3,5-dimethoxystilbene, 2',4,4'-trimethylchalcone, 3-cyanochromone, 2,7-dihydroxynaphthalene, psoralen, and 9-fluorenone. While ESI might not be the method of choice for identifying polycyclic aromatic hydrocarbons (PAHs), we were able to identify xanthone, which is an oxygenated polycyclic aromatic hydrocarbon (O-PAH), within the mixture of the 100 authentic BrC standards. Nonetheless, light-absorbing PAHs were not detected in our aerosol filter samples (Fig. S2), possibly due to their low abundance in emissions from smoldering-dominated burns.[37] Mixed-combustion conditions may generate O-PAHs that require other characterization techniques.

Text S5. Justification for and Limitations of Molecular-Level Analysis Methodology

In this section, we detail methodological adjustments in our molecular-level chemical analysis, acknowledge potential limitations, and provide justifications for our chosen methods. Our study implemented methodological adjustments aimed at reducing ESI suppression for aerosol filter samples, by mitigating potential contamination and sample losses, and developing an extended elution program to improve species separation and minimize co-elution. Previous studies[32,33] utilized methanol as the organic mobile phase and employed a faster chromatographic method for a more targeted approach. This work found that acetonitrile provided better peak separation and shape, which allowed for retention and identification of more compounds as outlined in this work. Elution time remained consistent throughout the analyses for all target analytes and issues possibly related to poor mixing was not observed. We further simulated complex aerosol filter samples using BrC standards, comprising an external mixture of 100 species. However, we recognize that such complexity may not fully reflect the actual extract composition. To gain a clearer insight into how aspects such as sample chemical composition influences the ionization efficiency of organic carbonaceous substances in ESI mass spectrometry, more in-depth studies are warranted. Potential approaches might involve preparing matrix-matched standards and devising appropriate correction factors. Adopting the standard addition technique would be optimal for quantification while considering the matrix effects of aerosol samples. However, a limitation is the necessity for multiple identical samples for this approach, achievable using an entire filter sheet from high-volume filters. Despite this, our key findings, such as the correlation between mass closure and aethalometer-based absorptivity, are consistent, even when surrogate standards are factored in (Sect. 3.2.1). Most of the identified BrC mass is represented by authentic standards, which maintain a relatively stable fractional contribution to the total identified BrC mass across samples. We do, however, recognize that surrogate standards do not perfectly mimic a target compound in terms of ESI response, and our method might introduce currently unquantifiable uncertainties (e.g., ionization suppression/enhancement, peak integration errors, repeatability errors in burn experiments and sample preparation, reproducibility, filter sampling artefacts). Therefore, future methods similar to ours that also rely on surrogate standard analysis should undergo rigorous evaluation and validation, with a comprehensive error analysis approach put in place.

In our study, we opted not to pursue DAD absorbance closure or juxtapose it with aethalometer-based aerosol absorption coefficients due to several considerations: (1) There exist inherent uncertainties with filter sampling and offline sample preparation, e.g., solvent extraction, which could introduce bias (for instance, extraction in methanol can lead to conversion of carbonyls into hemiacetals, and other methanol reactions; this was shown to be a problem for analysis of SOA by similar methods.[38,39] In addition, sonication has been shown to cause additional oxidation of analytes by the free radicals formed in the sonication process[40,41]); (2) The mass absorption coefficient spectra for identified BrC species in methanol solution that were quantified using surrogate standards remain undefined; (3) The total absorbance of individual BrC molecules dissolved in methanol and separated by LC (previously reported to significantly contribute to total integrated DAD absorbance[37,42]) is fundamentally different from both the UV-vis absorbance of the bulk solution[37] (not measured in this study) and particle-phase aerosol absorption. Potential discrepancies might stem from the existence of highly absorptive materials that ESI fails to detect or RPLC fails to separate efficiently (e.g., see the broad background absorbance in Fig. S2, which lacks a clearly defined baseline); (4) Intermolecular reactions, such as charge transfer complexes within the aerosol particles,[43] may be reduced in or absent from solvent-extracted molecules. The altered chemical environment can also induce solvatochromic shifts;[44] (5) To convert solvent extract absorbance into particle-phase absorption coefficients, one must account for numerous factors. These include temperature, aerosol pH, RH/phase state[45,46], particle morphology, size and mixing state,[47] differences in mass concentration and refractive indices in the particle phase versus the extracted solution. The complex calculations entailed by these requirements remain beyond the scope of this study. While sophisticated studies focusing on these aspects could yield valuable insights, our primary objective was to validate the mass-quantification methodology for various biomass fuel types and aging conditions in the chamber. We aimed to correlate the aethalometer-based aerosol absorptivity with the BrC mass closure result, considering that the PM mass in smoldering-dominated burns is mainly composed of OA. We narrowed our focus to the aethalometer's shortest wavelength (370 nm) as it accounts for the bulk of real-time absorption in our samples and is where most target chromophoric species absorb light, as shown in Fig. S3.

Text S6. Hierarchical Clustering

To gain a deeper understanding of the molecular-level composition of BrC in our samples, we turned to ClustVis, a web tool designed for visualizing clustering of multivariate data.[48] This tool allowed us to visualize the data in heatmaps and reduce its dimensionality through clustering. The input data matrix consisted of the fractional contribution to BrC mass by 182 BrC species across the 35 aerosol filter samples (6,370 matrix data points). For EIC peak area data points that fell below the linear range (Table S2), we replaced them with the mean value between zero and the lower limit of that range. Unit variance scaling was applied to rows (compounds labeled by their ID; Table S4), with both rows and columns clustered using correlation distance and average linkage. Annotations provided on the heatmap highlight distinct, well-separated molecular groups. When interpreting the clustering on the heatmap, focus should be placed

on the objects that merge into the clustering tree first, rather than the exact order of rows or columns. Swapping any two branches does not change the tree's meaning. We highlight specific clusters in rows and columns (colored boxes in Fig. S7) as a visual aid for interpreting the complex molecular-level composition dataset. This approach allows for a clearer understanding of patterns and relationships within the data,[49,50] helping to elucidate the underlying structure and organization of BrC species in aerosol filter samples.

Text S7. Additional Results on BrC Molecular-Level Composition in Non-Hardwood Derived Emissions

Primary BrC Aerosol Species from Burning Savanna Grass, Leaves, and Cow-Dung. P-coumaric acid ($C_9H_8O_3$, #68) forms 0.02-0.08% of PM mass in primary emissions from grass and leaf burning. Probable structural isomers of coumaric acid (RTs at 11.5 min, *#28*, and 13.5 min, *#41*) are more prevalent, contributing up to 0.4% in hardwood fuel emissions, and up to 1.0% for savanna grass in the dry chamber. Trans-ferulic acid ($C_{10}H_{10}O_4$, #74) contributes over 0.03% to PM mass in primary emissions from grass burning and is one of the key contributors to BrC mass in these emissions. Other contributors (those ≥0.01% of PM mass) to grass burning include vanillic and syringic acids, 3,4-dihydroxybenzaldehyde, acetosyringone, coniferaldehyde, 5-hydroxymethylfurfural (#7), hydroxybenzoic acids (*#21*, #35), methoxycinnamic acids (*#70*, #112), (di)hydroxycoumarins (*#23*, *#45*), *hydroxy-methylcoumarin* (#62), trihydroxy-*dimethoxyflavone* (#124), and *dihydroxystilbene* (#133). Primary BrC aerosol species from leaf burning comprise contributions from *hydroxy-methoxybenzaldehyde* (*#34*), *resorcinol*, tyrosol, *methylcatechol*, vanillic, p-coumaric and syringic acids, dihydroxy-benzaldehydes (#18, *#21*), 3-hydroxybenzoic acid (#35), *hydroxy-methoxybenzaldehyde* (#30), and *di/tri-hydroxystilbenes* (#129, #133). Moreover, *dihydroxy-trimethoxyflavone* (#157), 1-phenyl-1,3-butanedione, vanillin, trans-4-methoxycinnamic acid, sinapaldehyde, vanillic acid, 3,4-dihydroxybenzaldehyde, syringaldehyde, and trans-4-methoxycinnamic acid (#112) contribute to BrC in primary-aerosol emissions from cow-dung burning; other contributors include *trihydroxystilbene* (#129), *dihydroxy-trimethoxystilbenes* (#122, #134), and 3'-hydroxypterostilbene (#151).

BrC Species in Photochemically-Aged Emissions from Burning Mokala Branches. *Dihydroxy-trimethoxychalcone* ($C_{18}H_{18}O_6$, #139), *methyl gallate* ($C_8H_8O_5$, #13), *coniferyl alcohol* ($C_{10}H_{12}O_3$, #14), (homo-)vanillic and syringic acids, syringaldehyde, *tetrahydroxy-stilbene* ($C_{14}H_{12}O_4$, #138), and *dihydroxy-trimethoxystilbene* ($C_{17}H_{18}O_5$, #122) contribute to BrC mass in photochemically-aged emissions from burning mokala branches.

Table S1. Detailed information on the 35 chamber- and ambient-aerosol samples collected onto Teflon filters that were used for analyzing the molecular composition of BrC. For chamber-aerosol samples, the PM mass collected on each filter was estimated using the mean SMPS particle size distribution during sampling (the volume size distribution rarely extended >600 nm; the chamber-inlet cyclone cut-off size is 2.5 µm), the particle effective density (1.2 ± 0.1 g cm^{-3}) as well as the sampling flow rate (30 L min^{-1}) and duration (10-20 min.). For ambient samples (rows with light gray shading; IDs correspond to the Teflon ring code), the collected PM mass was determined by weighing the filters pre- and post-aerosol collection, with the mass difference representing the total suspended PM mass. For primary emissions ("*a*") in each experiment, emission factors, either mass-based (EF, in g kg^{-1}) or optical absorption-based (AEF, in m^2 kg^{-1}), along with the average modified combustion efficiency (MCE), were calculated. The table includes the mean relative humidity (RH) inside the smog chamber during each filter sampling period. Due to periods of gas analyzer malfunctions, some MCE values are missing.

Sample description	Burn #	Sample ID	Fuel mass (g)	MCE	RH (%)	PM mass (µg)	MAC_{370nm} (m^2/g)	% BrC contr. to PM	EF_{PM} (g/kg)	$EF_{combined-BrC}$	AEF_{370nm}	AEF_{470nm}	AEF_{660nm}
Acacia_RH_primary	6	6a	0.4663	n.a.	70	338	1.2	5.8	16	0.89	18	5.2	1.9
Wanza_dry_primary	8	8a	0.4098	0.84	0	192	1.9	13	16	2.2	32	10	4.0
Wanza_RH_primary	9	9a	0.4174	0.84	73	249	1.5	7.5	18	1.4	28	8.5	3.2
Wanza_RH_photo_aged	9	9c			32	104	1.3	3.9					
Mukusi_RH_primary	10	10a	0.4004	0.79	72	309	1.6	7.3	23	1.7	37	9.4	2.9
Mukusi_RH_photo_aged	10	10c			33	111	1.5	4.5					
Mopane_dry_primary	12	12a	0.3682	0.78	0	174	1.4	11	15	1.8	24	5.4	1.8
Mopane_dry_dark_aged	12	12b			2.5	99	2.2	14					
Mopane_RH_primary	13	13a	0.4151	0.82	77	300	1.3	2.4	22	0.54	29	6.6	2.2
Dung_RH_primary	15	15a	0.3520	0.88	70	159	0.71	0.41	14	0.061	10	2.9	1.1
Dung_RH_photo_aged	15	15c			31	106	0.56	0.50					
Mosetlha_RH_primary	17	17a	0.3940	n.a.	64	298	0.99	2.1	16	0.36	17	4.1	1.4
Grass_RH_primary	18	18a	0.5009	0.75	71	309	0.48	1.8	19	0.34	9.0	1.9	0.90
Grass_dry_primary	19	19a	0.4568	0.75	6	342	0.67	4.2	15	0.66	10	2.2	0.82
Mokala_RH_primary	21	21a	0.3155	0.80	67	444	0.56	3.6	29	1.0	16	3.2	0.91
Mokala_RH_photo_aged	21	21c			34	166	0.55	1.4					
Leaves_RH_primary	23	23a	0.4060	0.89	71	233	0.24	0.62	19	0.13	5.1	1.4	0.65
Leaves_RH_photo_aged	23	23c			37	304	0.37	0.73					
Leaves_dry_primary	24	24a	0.4915	0.85	5	354	0.24	0.94	23	0.23	5.9	1.4	0.74
FuelMix_RH_primary	25	25a	0.4526	0.90	70	360	0.58	3.1	24	0.74	14	2.9	0.90
FuelMix_RH_photo_aged	25	25c			44	491	0.46	1.3					
WildOlive_RH_primary	26	26a	0.3155	0.86	70	294	0.80	3.5	28	0.99	22	5.3	1.7
FuelMix_dry_primary	27	27a	0.4537	0.81	10	269	0.83	3.1	18	0.56	15	2.9	0.90
FuelMix_dry_photo_aged	27	27c			8	311	0.65	2.5					
Eucalyptus_RH_primary	28	28a	0.4471	0.84	72	348	0.88	3.5	24	0.82	21	3.9	1.4
BIUST_6/24/2022_daily		1P23149				350		0.090					
BIUST_7/1/2022_daily		1P23123				160		0.11					
BIUST_7/4/2022_daily		1P23121				310		0.22					
BIUST_7/8/2022_daily		1P23119				510		0.066					
BIUST_7/14-18/2022_night		1P23117				540		0.11					
BIUST_7/18-19/2022_day		1P23115				220		0.078					
BIUST_7/19-20/2022_night		1P23146				350		0.15					
BIUST_7/20/2022_day		1P23147				140		0.16					
Gaborone_7/15/2022_night		1P23113				1930		0.24					
Gaborone_7/16/2022_day		1P23114				1760		0.13					

Table S2. Authentic standards measured with the multi-stage platform and used to quantify 182 BrC species detected in aerosol filter samples, sorted by chemical class. Within each chemical class, the standards are listed in increasing mass order. RPLC-RTs are nearly identical for both ionization modes (± 0.1 min), with the exception of phthalic acid, possibly due to varying mobile phase compositions. Compound IDs match those in Table S4. 'Recovery' relates to standard compound detection in aerosol filter samples after sample preparation (Text S4). 'Neutral mass' refers to the average calculated from standard mixture samples used for creating calibration curves (2 ppb – 10 ppm), and Δ*m* is the mass difference between measured accurate and theoretically correct mass in ppm. Grey font denotes compounds not detected in aerosol samples but employed for surrogate analysis. Bold font indicates the quantification mode. The response has been adjusted for minimum purity and averaged for the duration of the campaign. The linear range of the extracted ion chromatogram (EIC) peak area, based on a 5 µL injection volume, was used in conjunction with mean response factors for surrogate standard-based quantifications.

**Name & Structure**	**Molecular formula**	**Vendor**	**Purity (%, ≥)**	**LC-RT (min.)**	**ID**	**Mode**	**Reco-very (%)**	**Neutral mass (Da)**	**Δ*m* (ppm)**	**CID neutral loss (Da)**	**Response factor (EIC area/ppm)**	**Concentration linear range**	**EIC peak area linear range**	R^2
Substituted guaiacols														
Vanillin 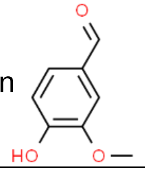	C8H8O3	Fisher Sci.	99	16.1	60	(-)<br>**(+)**	41 ± 15<br>39 ± 23	152.0466<br>152.0474	-4.6<br>0.7		$4.54\cdot10^{6}$<br>$6.79\cdot10^{4}$	10ppb-1ppm<br>0.1ppm-10ppm	$5\cdot10^{4}$ - $3\cdot10^{7}$<br>$3\cdot10^{4}$ - $1\cdot10^{6}$	0.999629<br>0.999780
Acetovanillone 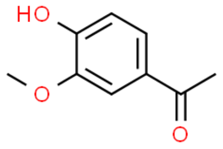	C9H10O3	Fisher Sci.	98	18.0	72	(-)<br>**(+)**	57 ± 18<br>59 ± 23	166.0628<br>166.0637	-1.2<br>4.2	124.1	$1.52\cdot10^{6}$<br>$5.59\cdot10^{5}$	10ppb-10ppm<br>10ppb-1ppm	$1\cdot10^{4}$ - $2\cdot10^{7}$<br>$6\cdot10^{3}$ - $2\cdot10^{6}$	0.999916<br>0.999850
Vanillic acid 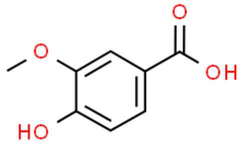	C8H8O4	Sigma	97	12.9	36	(-)	69 ± 16	168.0425	1.2		$1.66\cdot10^{6}$	10ppb-1ppm	$1\cdot10^{4}$ - $1\cdot10^{7}$	0.997361
Coniferaldehyde 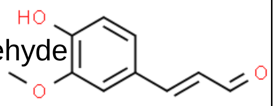	C10H10O3	TRC Canada	98	20.9	85	(-)<br>**(+)**	64 ± 16<br>70 ± 27	178.0631<br>178.0620	0.6<br>-5.6	46, 61, 72, 74, 88, 90, 100, 102, 114, 124.1	$6.16\cdot10^{6}$<br>$8.56\cdot10^{5}$	12ppb-1.2ppm<br>0.12ppm-12ppm	$6\cdot10^{4}$ - $4\cdot10^{7}$<br>$7\cdot10^{4}$ - $1\cdot10^{7}$	0.998811<br>0.999989
Homovanillic acid 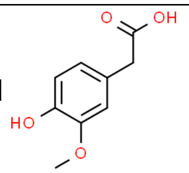	C9H10O4	Fisher Sci.	98	14.2	47	(-)	72 ± 17	182.0583	2.2		$1.23\cdot10^{6}$	10ppb-1ppm	$1\cdot10^{4}$ - $1\cdot10^{7}$	0.999633
Vanillylidenacetone 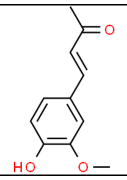	C11H12O3	TCI America	98	22.5	93	(-)<br>**(+)**	65 ± 16<br>72 ± 21	192.0788<br>192.0772	1.0<br>-7.3		$4.27\cdot10^{6}$<br>$1.34\cdot10^{6}$	10ppb-1ppm<br>0.1ppm-10ppm	$4\cdot10^{4}$ - $3\cdot10^{7}$<br>$7\cdot10^{4}$ - $2\cdot10^{7}$	0.999468<br>0.999911
(E)-Ferulic acid 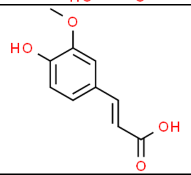	C10H10O4	Sigma	99	18.5	74	(-)<br>**(+)**	66 ± 18<br>68 ± 17	194.0571<br>194.0581	-4.1<br>1.0		$2.40\cdot10^{6}$<br>$1.41\cdot10^{5}$	10ppb-1ppm<br>0.1ppm-10ppm	$2\cdot10^{4}$ - $2\cdot10^{7}$<br>$6\cdot10^{3}$ - $7\cdot10^{5}$	0.997732<br>0.999963

Table S2 (continued)

**Name & Structure**	**Molecular formula**	**Vendor**	**Purity (%, ≥)**	**LC-RT (min.)**	**ID**	**Mode**	**Reco-very (%)**	**Neutral mass (Da)**	**Δ*m* (ppm)**	**CID neutral loss (Da)**	**Response factor (EIC area/ppm)**	**Concentration linear range**	**EIC peak area linear range**	***R^2***
							Substituted syringols							
Syringaldehyde 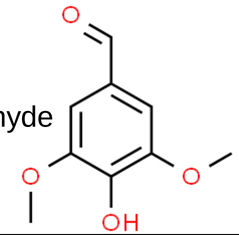	C9H10O4	Fisher Sci.	98	17.5	69	**(-)**<br>(+)	66 ± 18<br>54 ± 28	182.0579<br>182.0588	0.0<br>4.9	58, 114	$2.85 \cdot 10^{6}$<br>$9.67 \cdot 10^{4}$	10ppb-10ppm<br>0.1ppm-1ppm	$2 \cdot 10^{4}$ - $3 \cdot 10^{7}$<br>$1 \cdot 10^{4}$ - $4 \cdot 10^{5}$	0.999991<br>0.999976
Acetosyringone 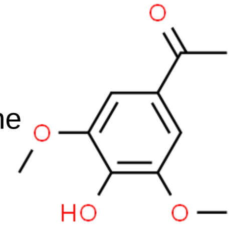	C10H12O4	Sigma	97	18.9	78	(-)<br>**(+)**	66 ± 17<br>66 ± 22	196.0737<br>196.0736	0.5<br>0.0	72.1, 154.1	$1.29 \cdot 10^{6}$<br>$8.96 \cdot 10^{5}$	10ppb-10ppm<br>10ppb-1ppm	$1 \cdot 10^{4}$ - $3 \cdot 10^{7}$<br>$7 \cdot 10^{3}$ - $3 \cdot 10^{6}$	0.999980<br>0.998565
Syringic acid 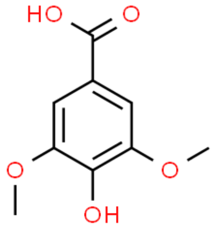	C9H10O5	Sigma	95	14.1	46	**(-)**<br>(+)	66 ± 20<br>58 ± 19	198.0536<br>198.0522	4.0<br>-3.0		$2.48 \cdot 10^{6}$<br>$2.50 \cdot 10^{4}$	12ppb-12ppm<br>1.2ppm-12ppm	$3 \cdot 10^{4}$ - $3 \cdot 10^{7}$<br>$3 \cdot 10^{4}$ - $3 \cdot 10^{5}$	0.999976<br>1.000000
Sinapaldehyde 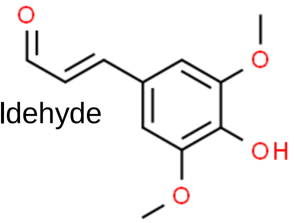	C11H12O4	TRC Canada	96	21.2	86	**(-)**<br>(+)	62 ± 15<br>65 ± 18	208.0733<br>208.0709	-1.4<br>-13.0		$3.66 \cdot 10^{6}$<br>$9.24 \cdot 10^{5}$	12ppb-12ppm<br>1ppb-1ppm	$4 \cdot 10^{4}$ - $5 \cdot 10^{7}$<br>$6 \cdot 10^{3}$ - $1 \cdot 10^{6}$	0.999863<br>0.986834
Sinapinic acid 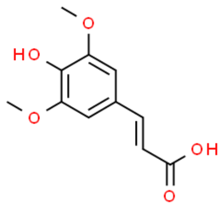	C11H12O5	TCI America	98	18.7	75	**(-)**<br>(+)	56 ± 17<br>45 ± 15	224.0676<br>224.0686	-4.0<br>0.4	76.1, 78, 134, 136, 148, 160	$2.22 \cdot 10^{6}$<br>$1.64 \cdot 10^{5}$	10ppb-1ppm<br>0.1ppm-10ppm	$2 \cdot 10^{4}$ - $2 \cdot 10^{7}$<br>$1 \cdot 10^{4}$ - $2 \cdot 10^{6}$	0.999997<br>0.999958

Table S2 (continued)

**Name & Structure**		**Molecular formula**	**Vendor**	**Purity (%, ≥)**	**LC-RT (min.)**	**ID**	**Mode**	**Reco-very (%)**	**Neutral mass (Da)**	**Δ*m* (ppm)**	**CID neutral loss (Da)**	**Response factor (EIC area/ppm)**	**Concentration linear range**	**EIC peak area linear range**	***R*²**
Other, substituted benzenes															
Benzoic acid	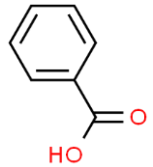	C7H6O2	Sigma	99.5	19.4	81	(-)	14 ± 6	122.0368	0.0		$4.08{\cdot}10^{6}$	3ppb-3ppm	$8{\cdot}10^{4}$ - $5{\cdot}10^{7}$	0.999963
4-Methylcatechol	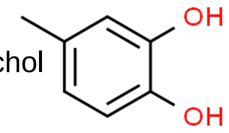	C7H8O2	Sigma	95	16.2	61	(-)	8 ± 8	124.0523	-0.8		$3.10{\cdot}10^{6}$	10ppb-1ppm	$1{\cdot}10^{4}$ - $3{\cdot}10^{7}$	0.999890
Pyrogallol	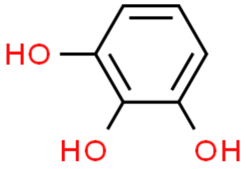	C6H6O3	Sigma	98	3.8	3	(-)	15 (*)	126.0321	3.2		$5.40{\cdot}10^{5}$	100ppb-1ppm	$2{\cdot}10^{4}$ - $5{\cdot}10^{6}$	0.999870
5-Hydroxymethylfurfural	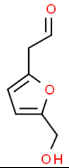	C6H6O3	Sigma	99	4.7	7	(+)	23 ± 10	126.0312	-4.0		$3.84{\cdot}10^{5}$	70ppb-0.7ppm	$1{\cdot}10^{4}$ - $1{\cdot}10^{6}$	0.998272
3,4-Dihydroxy-benzaldehyde	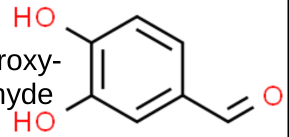	C7H6O3	Fisher Sci.	98	10.1	18	(-)<br>**(+)**	65 ± 21<br>70 ± 38	138.0317<br>138.0310	0.0<br>-5.1	 74	$3.18{\cdot}10^{6}$<br>$4.39{\cdot}10^{4}$	10ppb-10ppm<br>0.1ppm-10ppm	$2{\cdot}10^{4}$ - $4{\cdot}10^{7}$<br>$2{\cdot}10^{4}$ - $5{\cdot}10^{5}$	0.999957<br>0.999979
3-Hydroxy-benzoic acid	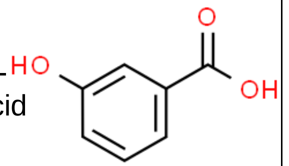	C7H6O3	Sigma	99	12.9	35	(-)	67 ± 17	138.0317	0.0	44	$5.15{\cdot}10^{6}$	20ppb-20ppm	$5{\cdot}10^{4}$ - $4{\cdot}10^{7}$	0.999981
Tyrosol	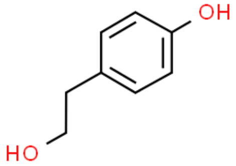	C8H10O2	Sigma	98	10.7	24	(-)	63 ± 17	138.0675	-4.3		$9.36{\cdot}10^{5}$	12ppb-12ppm	$1{\cdot}10^{4}$ - $1{\cdot}10^{7}$	0.995600
4-Methoxy-benzoic acid	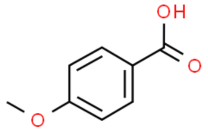	C8H8O3	Sigma	99	21.7	90	(-)	66 ± 15	152.0467	-3.9		$1.74{\cdot}10^{6}$	12ppb-1.2ppm	$2{\cdot}10^{4}$ - $7{\cdot}10^{6}$	0.997686

(*) this species was not detected at 0.5 ppm in spiked filter extracts, likely due to low recovery (considering its concentration linear range), therefore the mean recovery of benzoic acid, 4-methylcatechol and 5-hydroxymethylfurfural was applied.

Table S2 (continued)

Name & Structure	Molecular formula	Vendor	Purity (%, ≥)	LC-RT (min.)	ID	Mode	Reco-very (%)	Neutral mass (Da)	Δ*m* (ppm)	CID neutral loss (Da)	Response factor (EIC area/ppm)	Concentration linear range	EIC peak area linear range	R^2
Other, substituted benzenes *(continued)*														
1-Phenyl-1,3-butanedione 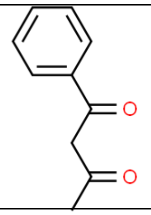	C10H10O2	TCI America	98	16.7	64	(+)	68 ± 21	162.068	-0.6	32, 60, 72, 86, 112.1	$1.26\cdot10^{5}$	0.1ppm-1ppm	$7\cdot10^{3}$ - $2\cdot10^{6}$	0.996002
p-Coumaric acid 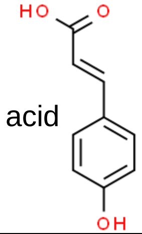	C9H8O3	Sigma	98	16.9	68	(-)<br>**(+)**	66 ± 18<br>62 ± 37	164.0442<br>164.0477	-18.9<br>2.4		$3.96\cdot10^{6}$<br>$6.40\cdot10^{4}$	13ppb-13ppm<br>0.1ppm-1ppm	$4\cdot10^{4}$ - $7\cdot10^{7}$<br>$4\cdot10^{3}$ - $2\cdot10^{5}$	0.999991<br>0.995060
Terephthalic acid 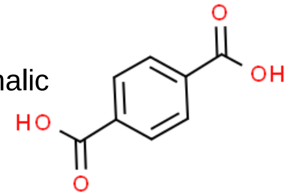	C8H6O4	Fisher Sci.	98	11.3	25	(-)	70 ± 20	166.0266	0.0		$6.35\cdot10^{6}$	13ppb-1.3ppm	$6\cdot10^{4}$ - $3\cdot10^{7}$	0.999924
Phthalic acid 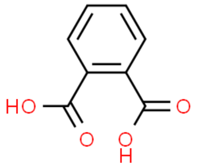	C8H6O4	Fisher Sci.	99	10.4<br>12.2	20	**(-)**<br>(+)	62 ± 18<br>75 ± 12	166.0267<br>166.0266	0.6<br>0.0	18, 46, 74, 102	$2.32\cdot10^{6}$<br>$1.75\cdot10^{5}$	0.12ppm-1.2ppm<br>0.12ppm-12ppm	$3\cdot10^{4}$ - $3\cdot10^{7}$<br>$1\cdot10^{4}$ - $2\cdot10^{6}$	0.993051<br>0.999999
(E)-4-Methoxy-cinnamic acid 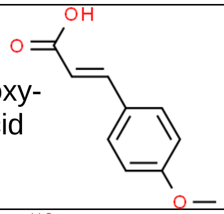	C10H10O3	Fisher Sci.	98	26.6	112	**(-)**<br>(+)	66 ± 16<br>69 ± 44	178.0632<br>178.0612	1.1<br>-10.1		$3.28\cdot10^{6}$<br>$2.41\cdot10^{5}$	12ppb-1.2ppm<br>0.12ppb-1.2ppm	$3\cdot10^{4}$ - $2\cdot10^{7}$<br>$1\cdot10^{4}$ - $1\cdot10^{6}$	0.999937<br>0.998713
Caffeic acid 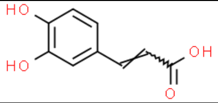	C9H8O4	TCI America	98	13.4	-	(+)	42 ± 19	180.0419	-2.2		$8.33\cdot10^{4}$	0.1ppm-10ppm	$3\cdot10^{3}$ - $2\cdot10^{5}$	0.996676
3,4-Dimethoxy-benzoic acid 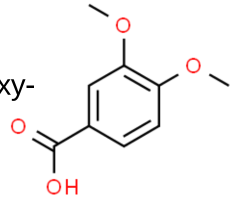	C9H10O4	TCI America	98	18.8	76	**(-)**<br>(+)	68 ± 16<br>59 ± 44	182.0580<br>182.0588	0.5<br>4.9	74	$4.44\cdot10^{6}$<br>$4.41\cdot10^{4}$	12ppb-1.2ppm<br>0.12ppm-1.2ppm	$5\cdot10^{4}$ - $2\cdot10^{7}$<br>$4\cdot10^{3}$ - $2\cdot10^{5}$	0.998689<br>0.999990
3,4-Dimethoxy-cinnamic acid 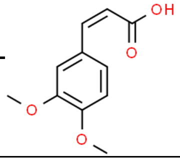	C11H12O4	TCI America	98	23.6	97	**(-)**<br>(+)	66 ± 17<br>74 ± 34	208.0740<br>208.0742	1.9<br>2.9		$7.02\cdot10^{6}$<br>$3.85\cdot10^{5}$	13ppb-1.3ppm<br>0.13ppm-1.3ppm	$5\cdot10^{4}$ - $4\cdot10^{7}$<br>$3\cdot10^{4}$ - $1\cdot10^{6}$	0.999882<br>0.999981

Table S2 (continued)

**Name & Structure**	**Molecular formula**	**Vendor**	**Purity (%, ≥)**	**LC-RT (min.)**	**ID**	**Mode**	**Reco-very (%)**	**Neutral mass (Da)**	**Δ*m* (ppm)**	**CID neutral loss (Da)**	**Response factor (EIC area/ppm)**	**Concentration linear range**	**EIC peak area linear range**	R^2
							Nitroaromatic compounds							
4-Nitrophenol 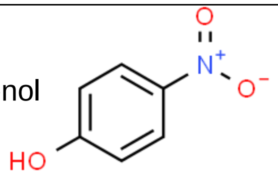	C6H5NO3	Fisher Sci.	99	21.3	87	(-)	65 ± 16	139.0258	-7.9		$2.41 \cdot 10^7$	10ppb-1ppm	$8 \cdot 10^4$ - $6 \cdot 10^7$	0.999680
4-Nitro-o-cresol 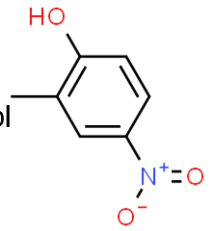	C7H7NO3	TCI America	98	28.0	117	(-)	66 ± 16	153.0427	0.7		$2.96 \cdot 10^7$	10ppb-1ppm	$1 \cdot 10^5$ - $7 \cdot 10^7$	0.999768
4-Nitrocatechol 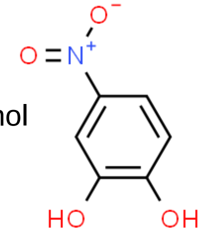	C6H5NO4	TCI America	98	16.8	66	(-)	57 ± 21	155.0214	-3.2		$3.39 \cdot 10^6$	10ppb-1ppm	$4 \cdot 10^4$ - $4 \cdot 10^7$	0.999997
2-Methyl-4-nitroresorcinol 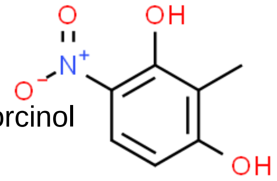	C7H7NO4	Sigma	n.a.	29.5	123	(-)	68 ± 16	169.0371	-2.4		$3.06 \cdot 10^7$	10ppb-1ppm	$2 \cdot 10^5$ - $3 \cdot 10^7$	0.999471
4-Nitroguaiacol 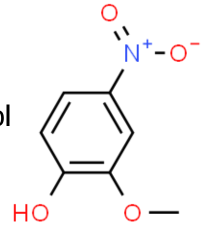	C7H7NO4	TCI America	98	22.9	95	(-)	55 ± 16	169.038	3.0	45, 73	$1.56 \cdot 10^7$	10ppb-1ppm	$8 \cdot 10^4$ - $5 \cdot 10^7$	0.999739
5-Nitrosalicylic acid 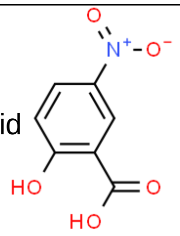	C7H5NO5	Fisher Sci.	99	19.1	80	(-)	67 ± 17	183.0167	-0.5		$1.66 \cdot 10^7$	10ppb-1ppm	$1 \cdot 10^5$ - $9 \cdot 10^7$	0.999929
4-Nitro-1-naphthol 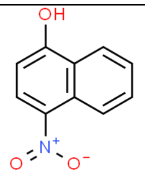	C10H7NO3	TCI America	98	34.3	145	(-)	67 ± 16	189.0428	1.1		$2.43 \cdot 10^7$	10ppb-1ppm	$2 \cdot 10^5$ - $6 \cdot 10^7$	0.999090

Table S2 (continued)

**Name & Structure**	**Molecular formula**	**Vendor**	**Purity (%, ≥)**	**LC-RT (min.)**	**ID**	**Mode**	**Reco-very (%)**	**Neutral mass (Da)**	**Δm (ppm)**	**CID neutral loss (Da)**	**Response factor (EIC area/ppm)**	**Concentration linear range**	**EIC peak area linear range**	***R^2***
							Coumarins							
Umbelliferone 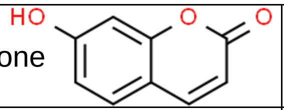	C9H6O3	TCI America	98	17.8	71	**(+)**	65 ± 24	162.0315	-1.2	86	$7.34\cdot10^5$	130ppb-1.3ppm	$5\cdot10^4$ - $3\cdot10^6$	0.998967
4-Hydroxycoumarin 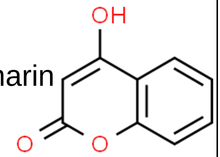	C9H6O3	TCI America	98	21.6	89	**(-)**<br>(+)	49 ± 11<br>63 ± 17	162.0314<br>162.0332	-1.9<br>9.3	 42, 98	$1.47\cdot10^7$<br>$1.58\cdot10^6$	2ppb-1ppm<br>10ppb-0.5ppm	$5\cdot10^4$ - $5\cdot10^7$<br>$1\cdot10^4$ - $1\cdot10^6$	0.999635<br>0.999789
4-Methyl-umbelliferone 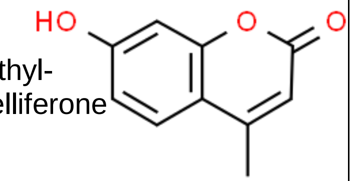	C10H8O3	Sigma	98	21.5	88	**(-)**<br>(+)	72 ± 16<br>71 ± 23	176.0472<br>176.0476	-0.6<br>1.7		$7.49\cdot10^6$<br>$1.48\cdot10^6$	3ppb-1.3ppm<br>13ppb-0.65ppm	$5\cdot10^4$ - $5\cdot10^7$<br>$1\cdot10^4$ - $1\cdot10^6$	0.995569<br>0.999938
7-Methoxy-coumarin 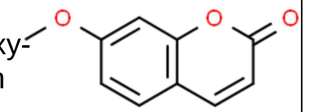	C10H8O3	Fisher Sci.	98	25.8	109	(+)	63 ± 27	176.0464	-5.1		$1.23\cdot10^6$	10ppb-0.1ppm	$1\cdot10^4$ - $3\cdot10^5$	1.000000
Esculetin 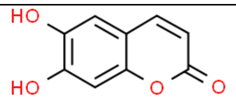	C9H6O4	TCI America	98	13.2	38	(+)	58 ± 19	178.026	-3.4		$6.00\cdot10^5$	13ppb-1.3ppm	$8\cdot10^3$ - $2\cdot10^6$	0.999754
7-Ethoxycoumarin 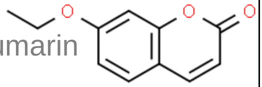	C11H10O3	Sigma	99.5	31.3	-	(+)	67 ± 30	190.0624	-3.2		$1.95\cdot10^6$	10ppb-1ppm	$1\cdot10^4$ - $6\cdot10^6$	0.999838
Scopoletin 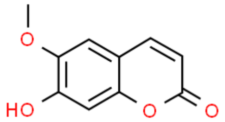	C10H8O4	TRC Canada	98	18.3	73	(-)	64 ± 16	192.0421	-1.0		$3.50\cdot10^6$	20ppb-2ppm	$6\cdot10^4$ - $3\cdot10^7$	0.999375
7-Hydroxy-3,4,8-trimethylcoumarin 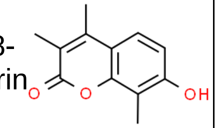	C12H12O3	Sigma	97	28.2	119	**(-)**<br>(+)	66 ± 16<br>70 ± 23	204.0792<br>204.0772	2.9<br>-6.9		$1.40\cdot10^7$<br>$2.22\cdot10^6$	10ppb-1ppm<br>10ppb-1ppm	$4\cdot10^4$ - $5\cdot10^7$<br>$2\cdot10^4$ - $2\cdot10^6$	0.997730<br>0.998871
5,7-Dimethoxy-coumarin 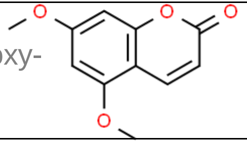	C11H10O4	Fisher Sci.	98	31.0	-	(+)	67 ± 30	206.0571	-3.9		$2.67\cdot10^6$	10ppb-1ppm	$3\cdot10^4$ - $6\cdot10^6$	0.999106
4-Ethyl-5,7-dimethoxycoumarin	C13H14O4	ChemBridge Corporation	n.a.	38.1	-	(+)	72 ± 29	234.0873	-8.1		$3.22\cdot10^6$	0.35ppm-7ppm	$2\cdot10^5$ - $3\cdot10^7$	0.999992
Nodakenetin 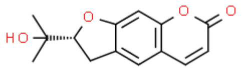	C14H14O4	TargetMol Chemicals	99.9	24.8	102	(-)	70 ± 21	246.0905	5.3		$2.00\cdot10^4$	0.25ppm-5ppm	$3\cdot10^3$ - $2\cdot10^5$	0.999858

Table S2 (continued)

**Name & Structure**	**Molecular formula**	**Vendor**	**Purity (%, ≥)**	**LC-RT (min.)**	**ID**	**Mode**	**Reco-very (%)**	**Neutral mass (Da)**	**Δ*m* (ppm)**	**CID neutral loss (Da)**	**Response factor (EIC area/ppm)**	**Concentration linear range**	**EIC peak area linear range**	***R^2***
Stilbenes														
3'-Hydroxy-pterostilbene 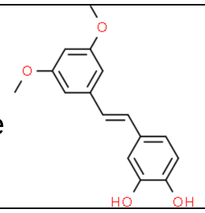	C16H16O4	TRC Canada	98	36.1	151	**(-)**<br>(+)	17 ± 9<br>24 ± 22	272.1058<br>272.1056	3.3<br>2.6	76.1, 80, 88.1, 92, 108	$7.08\cdot10^{6}$<br>$2.47\cdot10^{5}$	10ppb-1ppm<br>0.5ppm-10ppm	$1\cdot10^{4}$ - $3\cdot10^{7}$<br>$2\cdot10^{4}$ - $2\cdot10^{6}$	0.999655<br>0.999602
Combretastatin A1 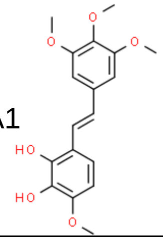	C18H20O6	Cayman	98	33.4	136	**(-)**<br>(+)	9 ± 9<br>2 ± 2	332.1273<br>332.1231	3.9<br>-8.7	170.1, 198.1	$5.43\cdot10^{6}$<br>$1.52\cdot10^{6}$	10ppb-1ppm<br>0.1ppm-1ppm	$2\cdot10^{4}$ - $3\cdot10^{7}$<br>$5\cdot10^{3}$ - $5\cdot10^{6}$	0.999729<br>0.995176
Flavonoids														
Flavone 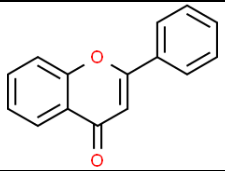	C15H10O2	TCI America	98	37.4	-	(+)	62 ± 23	222.068	-0.5	94, 120, 130, 146, 158, 172.1	$7.51\cdot10^{6}$	10ppb-1ppm	$6\cdot10^{4}$ - $3\cdot10^{7}$	0.999798
2',4,4'-Trimethyl-chalcone 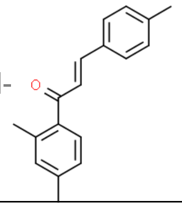	C18H18O	Sigma	n.a.	51.8	-	(+)	61 ± 27	250.1324	-13.6	106.1, 134.1, 136.1, 160.1	$3.84\cdot10^{6}$	12ppb-1.2ppm	$2\cdot10^{4}$ - $8\cdot10^{6}$	0.975013
(E)-2'-Hydroxy-4',6'-dimethoxy chalcone (Flavokawain B) 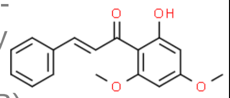	C17H16O4	TRC Canada	97	50.1	-	(+)	22 ± 11	284.1043	-2.1	104.1	$3.25\cdot10^{6}$	5ppb-5ppm	$2\cdot10^{4}$ - $7\cdot10^{6}$	0.999884
Kaempferol 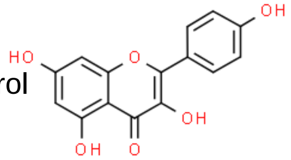	C15H10O6	TRC Canada	97	29.8	125	**(-)**<br>(+)	6 ± 5<br>9 ± 13	286.0481<br>286.0457	1.4<br>-7.0	134, 166	$5.00\cdot10^{6}$<br>$4.65\cdot10^{5}$	10ppb-1ppm<br>0.1ppm-1ppm	$2\cdot10^{4}$ - $3\cdot10^{7}$<br>$2\cdot10^{4}$ - $3\cdot10^{6}$	0.998751<br>0.990984
2,4,4'-Trimethoxy-chalcone 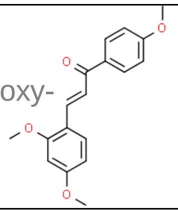	C18H18O4	Sigma	n.a.	45.2	-	(+)	35 ± 14	298.122	5.0		$6.39\cdot10^{6}$	2ppb-0.1ppm	$2\cdot10^{4}$ - $2\cdot10^{6}$	0.999971

Table S2 (continued)

**Name & Structure**	**Molecular formula**	**Vendor**	**Purity (%, ≥)**	**LC-RT (min.)**	**ID**	**Mode**	**Reco-very (%)**	**Neutral mass (Da)**	**Δ*m* (ppm)**	**CID neutral loss (Da)**	**Response factor (EIC area/ppm)**	**Concentration linear range**	**EIC peak area linear range**	***R^2***
							***Flavonoids** (continued)*							
Diosmetin 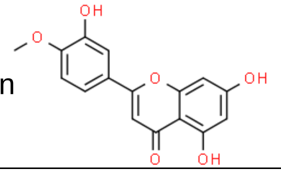	C16H12O6	TCI America	98	30.1	127	**(-)**<br>(+)	63 ± 15<br>69 ± 19	300.0648<br>300.0618	4.7<br>-5.3		$7.52 \cdot 10^6$<br>$1.10 \cdot 10^6$	10ppb-1ppm<br>10ppb-1ppm	$3 \cdot 10^4$ - $4 \cdot 10^7$<br>$5 \cdot 10^3$ - $5 \cdot 10^6$	0.999345<br>0.999580
2',4-Dihydroxy-4',6'-dimetho-xychalcone (Flavokawain C) 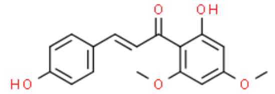	C17H16O5	TargetMol Chemicals	98	41.1	171	**(-)**<br>(+)	45 ± 9<br>32 ± 17	300.0928<br>300.1001	-23.3<br>1.0		$2.34 \cdot 10^6$<br>$1.61 \cdot 10^6$	7ppb-0.7ppm<br>7ppb-0.7ppm	$9 \cdot 10^4$ - $4 \cdot 10^7$<br>$8 \cdot 10^3$ - $5 \cdot 10^6$	0.755563<br>0.997839
6,2′,4′-Trimethoxy -flavone 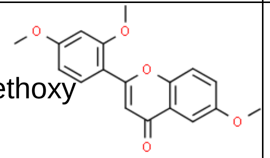	C18H16O5	Sigma	98	41.0	169	(+)	75 ± 25	312.0981	-5.4	58	$9.41 \cdot 10^6$	10ppb-0.5ppm	$7 \cdot 10^4$ - $6 \cdot 10^6$	0.999965
3-(2,4-Dimethoxyphenyl)-1-(4-ethoxyphenyl)-2-propen-1-one (Methyl-trimethoxychalcone) 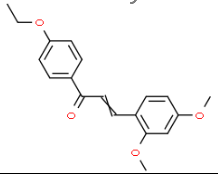	C19H20O4	ChemBridge Corporation	n.a.	48.7	-	(+)	42 ± 17	312.1369	2.2	192.1	$5.38 \cdot 10^6$	2ppb-0.1ppm	$1 \cdot 10^4$ - $1 \cdot 10^6$	0.999908
2′-Hydroxy-4,4′,6′-trimethoxy-chalcone (Flavokawain A) 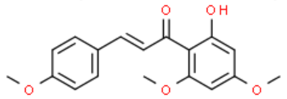	C18H18O5	TCI America	98	49.7	-	(+)	25 ± 13	314.114	-4.5		$3.86 \cdot 10^6$	10ppb-1ppm	$2 \cdot 10^4$ - $5 \cdot 10^6$	0.993646
1,3-Bis(2,4-dimethoxy-phenyl)-2-propen-1-one (Tetramethoxychalcone) 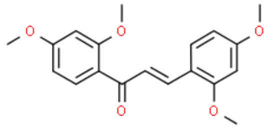	C19H20O5	ChemBridge Corporation	n.a.	44.1	-	(+)	43 ± 16	328.1292	-5.8		$8.51 \cdot 10^6$	5ppb-250ppb	$3 \cdot 10^4$ - $3 \cdot 10^6$	0.998283
4-Hydroxy-2′,3,4′,6′-tetramethoxychalcone 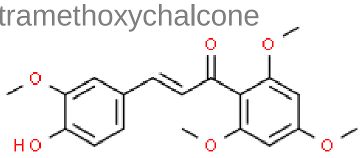	C19H20O6	Sigma	n.a.	33.6	-	(+)	76 ± 26	344.1253	-2.0		$5.35 \cdot 10^6$	12ppb-1.2ppm	$1 \cdot 10^5$ - $3 \cdot 10^7$	0.999994

Table S3. Molecular formulas and corresponding masses derived from (+)/(-)ESI tandem MS for all fragment ions identified in BrC standards (CID neutral losses; Table S2). IDs are provided for formulas with the same mass at unit-mass resolution (as reported by MassHunter).

Fragment neutral mass (Da)	Fragment neutral formula(s)
18	H2O
32	CH4O
42	C2H2O
44	CO2
45	CH3NO
46	CH2O2
58	C3H6O
60	C2H4O2
61	C2H5O2
72	C3H4O2
72.1	C4H8O
73	C2H3NO2
74	C2H2O3
76.1	C3H8O2
78	C2H6O3
80	C2H8O3
86	C4H6O2 (#64), C3H2O3 (#71)
88	C3H4O3
88.1	C4H8O2
90	C3H6O3
92	C3H8O3
94	C6H6O
98	C4H2O3
100	C4H4O3
102	C3H2O4 (#20), C4H6O3 (#85), C8H6 (#125)
104.1	C8H8
106.1	C8H10
108	C3H8O4
112.1	C6H8O2
114	C5H6O3
120	C7H4O2
124.1	C7H8O2
130	C9H6O
134	C4H6O5 (#75), C8H6O2 (#125)
134.1	C9H10O
136	C4H8O5
136.1	C9H12O
146	C9H6O2
148	C5H8O5
154.1	C8H10O3
158	C10H6O2
160	C6H8O5
160.1	C11H12O
166	C8H6O4
170.1	C6H18O5
172.1	C11H8O2
192.1	C12H16O2
198.1	C7H18O6

Table S4. The 182 separated BrC (DAD-absorbing) species identified in chamber- and ambient-aerosol filter samples using RPLC coupled to (-)/(+)ESI-HR-QTOFMS/MS. The species are arranged according to RT, with an effective separation protocol time window of 2.5-50.5 min and RPLC/ESI-HR-QTOFMS/MS delay time of 0.15 min. No detectable signal for these species was found in blank filter chromatograms. The aerosol sample-mean measured (neutral) mass, accuracy (Δm, in ppm), and RT of each species are provided. Chemical classes include: SG (substituted syringols), G (substituted guaiacols), O (other, substituted benzenes), N (nitroaromatic compounds), C (coumarins), S (stilbenes), and F (flavonoids). For species detected in both modes, the quantification mode is highlighted in bold. Mass quantification employed either authentic (A) or surrogate (S) standards, selected based on MassHunter-suggested molecular formulas, expected chemical family/structure (assisted by ChemSpider), and DAD absorbance spectrum/RT similarity to authentic standards. Grey color indicates surrogate standards with differing formulas but similar structure/RT. When multiple surrogates were used for a single BrC species, their mean response factor was utilized for quantification. Notably, the majority of the total identified BrC mass across the aerosol filter sample extracts corresponded to species with a matching authentic standard.

ID	LC-RT (min.)	Molecular Formula	Neutral Mass (Da)	Δm (ppm)	Quantitation with A or S?	Name (if A)	Tentative aromatic structure (if S)	Chemical Class	Ion mode	Surrogate standard(s) used for quantification	Formula of surrogate(s)	LC-RT of surrogate(s)
**1**	2.60	C6H4O4	140.0105	-3.3	S		carboxy-furfural	O	(+)	5-hydroxymethylfurfural	C6H6O3	4.70
**2**	3.32	C6H5NO3	139.027	0.4	S		nitrophenol	N	(-)	pyrogallol<br>4-nitrophenol	C6H6O3<br>C6H5NO3	3.76<br>21.28
**3**	3.76	C6H6O3	126.0319	1.6	A	pyrogallol		O	(-)			
**4**	4.00	C7H6O4	154.0268	1.2	S		dihydroxybenzoic acid	O	(-)	3,4-dihydroxybenzaldehyde<br>3-hydroxybenzoic acid	C7H6O3<br>C7H6O3	10.06<br>12.91
**5**	4.02	C6H6O2	110.0373	4.7	S		catechol	O	(-)	pyrogallol	C6H6O3	3.76
**6**	4.61	C7H6O4	154.0266	-0.1	S		dihydroxybenzoic acid	O	(-)	3,4-dihydroxybenzaldehyde<br>3-hydroxybenzoic acid	C7H6O3<br>C7H6O3	10.06<br>12.91
**7**	4.70	C6H6O3	126.0315	-1.7	A	5-hydroxymethylfurfural		O	(+)			
**8**	5.35	C6H5NO4	155.0221	1.6	S		nitrocatechol	N	(-)	pyrogallol<br>4-nitrocatechol	C6H6O3<br>C6H5NO4	3.76<br>16.77
**9**	6.41	C7H6O4	154.0266	-0.1	S		dihydroxybenzoic acid	O	(-)	3,4-dihydroxybenzaldehyde<br>3-hydroxybenzoic acid	C7H6O3<br>C7H6O3	10.06<br>12.91
**10**	6.73	C8H10O3	154.0629	-0.6	S		syringol<br>hydroxytyrosol	SG	(-)	pyrogallol<br>tyrosol	C6H6O3<br>C8H10O2	3.79<br>10.69
**11**	6.79	C6H6O2	110.0368	0.2	S		resorcinol	O	(-)	pyrogallol	C6H6O3	3.76
**12**	7.61	C6H5NO3	139.0272	1.8	S		nitrophenol	N	(-)	pyrogallol<br>3-hydroxybenzoic acid<br>4-nitrophenol	C6H6O3<br>C7H6O3<br>C6H5NO3	3.76<br>12.91<br>21.28
**13**	8.75	C8H8O5	184.0369	-1.5	S		methyl gallate	O	(-)	vanillic acid<br>syringic acid	C8H8O4<br>C9H10O5	12.92<br>14.07
**14**	9.11	C10H12O3	180.0773	5.3	S		coniferyl alcohol	G	(-) / **(+)**	coniferaldehyde	C10H10O3	20.86
**15**	9.36	C7H8O2	124.0526	1.4	S		methylcatechol	O	(-)	3,4-dihydroxybenzaldehyde<br>3-hydroxybenzoic acid<br>4-methylcatechol	C7H6O3<br>C7H6O3<br>C7H8O2	10.06<br>12.91<br>16.15
**16**	9.37	C8H8O4	168.0424	0.8	S		hydroxy-methoxybenzoic acid	O	(-)	3,4-dihydroxybenzaldehyde<br>vanillic acid	C7H6O3<br>C8H8O4	10.06<br>12.92
**17**	9.98	C8H6O5	182.0202	-7.3	S		hydroxyphthalic acid	O	(-)	3,4-dihydroxybenzaldehyde<br>phthalic acid	C7H6O3<br>C8H6O4	10.06<br>10.41
**18**	10.06	C7H6O3	138.0310	-4.8	A	3,4-dihydroxybenzaldehyde		O	(-) / **(+)**			
**19**	10.29	C7H7NO4	169.0376	0.5	S		methoxy-nitrophenol<br>methyl-nitrocatechol	N	(-)	4-nitrocatechol	C6H5NO4	16.77
**20**	10.41	C8H6O4	166.0264	-1.3	A	phthalic acid		O	**(-)** / (+)			
**21**	10.60	C7H6O3	138.0316	-0.7	S		dihydroxybenzaldehyde<br>hydroxybenzoic acid	O	(-)	3,4-dihydroxybenzaldehyde<br>3-hydroxybenzoic acid	C7H6O3<br>C7H6O3	10.06<br>12.91
**22**	10.61	C6H6O	94.0421	2.5	S		phenol	O	(-)	tyrosol	C8H10O2	10.69
**23**	10.66	C9H6O4	178.0262	-2.4	S		dihydroxycoumarin	C	(+)	esculetin	C9H6O4	13.24
**24**	10.69	C8H10O2	138.0671	-7.1	A	tyrosol		O	(-)			
**25**	11.26	C8H6O4	166.0257	-5.5	A	terephthalic acid		O	(-)			
**26**	11.28	C7H6O2	122.0358	-8.0	S		hydroxy-benzaldehyde	O	(-)	3,4-dihydroxybenzaldehyde<br>3-hydroxybenzoic acid	C7H6O3<br>C7H6O3	10.06<br>12.91
**27**	11.39	C9H10O4	182.0572	-3.9	S		dimethoxybenzoic acid	O	**(-)** / (+)	3,4-dimethoxybenzoic acid	C9H10O4	18.75

ID	LC-RT (min.)	Molecular Formula	Neutral Mass (Da)	Δm (ppm)	Quantitation with A or S?	Name (if A)	Tentative aromatic structure (if S)	Chemical Class	Ion mode	Surrogate standard(s) used for quantification	Formula of surrogate(s)	LC-RT of surrogate(s)
**28**	11.46	C9H8O3	164.0466	-4.6	S		coumaric acid	O	(-) / **(+)**	p-coumaric acid	C9H8O3	16.94
**29**	12.10	C9H10O3	166.0621	-5.4	S		hydroxy-methoxyacetophenone	O	(-)	vanillin<br>acetovanillone	C8H8O3<br>C9H10O3	16.13<br>18.02
**30**	12.15	C8H8O3	152.047	-2.3	S		hydroxy-methoxybenzaldehyde	O	(-)	3,4-dihydroxybenzaldehyde<br>vanillin	C7H6O3<br>C8H8O3	10.06<br>16.13
**31**	12.36	C8H8O4	168.0419	-2.1	S		hydroxy-methoxybenzoic acid	O	(-)	vanillic acid	C8H8O4	12.92
**32**	12.39	C7H6O4	154.0253	-8.5	S		dihydroxybenzoic acid	O	(-)	3,4-dihydroxybenzaldehyde<br>3-hydroxybenzoic acid	C7H6O3<br>C7H6O3	10.06<br>12.91
**33**	12.61	C7H6O3	138.0294	-16.6	S		hydroxybenzoic acid	O	(-)	3-hydroxybenzoic acid	C7H6O3	12.91
**34**	12.63	C8H8O3	152.0465	-5.6	S		hydroxy-methoxybenzaldehyde /<br>vanillin isomer	O	(-) / **(+)**	3,4-dihydroxybenzaldehyde<br>vanillin	C7H6O3<br>C8H8O3	10.06<br>16.13
**35**	12.91	C7H6O3	138.0313	-2.9	A	3-hydroxybenzoic acid		O	(-)			
**36**	12.92	C8H8O4	168.0421	-1.0	A	vanillic acid		G	(-)			
**37**	12.96	C7H8O2	124.0519	-4.3	S		methylcatechol	O	(-)	4-methylcatechol	C7H8O2	16.15
**38**	13.24	C9H6O4	178.0257	-5.1	A	esculetin		C	(+)			
**39**	13.46	C10H12O4	196.0726	-4.9	S		dimethoxy-hydroxyacetophenone	O	(-)	acetosyringone	C10H12O4	18.87
**40**	13.50	C9H10O4	182.0571	-4.4	S		dimethoxy-hydroxybenzaldehyde	O	(-)	syringaldehyde	C9H10O4	17.47
**41**	13.50	C9H8O3	164.0466	-4.2	S		coumaric acid	O	(+)	p-coumaric acid	C9H8O3	16.94
**42**	13.59	C7H7NO3	153.042	-3.9	S		methyl-nitrophenol	N	(-)	4-nitroguaiacol<br>4-nitro-o-cresol	C7H7NO4<br>C7H7NO3	22.92<br>27.99
**43**	13.65	C7H6O2	122.0365	-2.3	S		hydroxy-benzaldehyde	O	(-)	3-hydroxybenzoic acid	C7H6O3	12.91
**44**	13.97	C7H7NO3	153.0421	-3.2	S		methyl-nitrophenol	N	(-)	4-nitroguaiacol<br>4-nitro-o-cresol	C7H7NO4<br>C7H7NO3	22.92<br>27.99
**45**	13.98	C9H6O3	162.0308	-5.8	S		hydroxycoumarin	C	(-) / **(+)**	umbelliferone<br>4-hydroxycoumarin	C9H6O3<br>C9H6O3	17.77<br>21.55
**46**	14.07	C9H10O5	198.0527	-0.6	A	syringic acid		SG	**(-)** / (+)			
**47**	14.22	C9H10O4	182.0571	-4.4	A	homovanillic acid		G	(-)			
**48**	14.62	C9H6O4	178.0256	-5.7	S		dihydroxycoumarin	C	(-)	umbelliferone	C9H6O3	17.77
**49**	14.84	C9H10O4	182.0577	-1.1	S		dimethoxy-hydroxybenzaldehyde	O	(+)	syringic acid<br>syringaldehyde	C9H10O5<br>C9H10O4	14.07<br>17.47
**50**	14.91	C9H10O4	182.0562	-9.4	S		homovanillic acid isomer	O	(-)	homovanillic acid	C9H10O4	14.22
**51**	14.94	C9H8O4	180.0415	-4.2	S		dihydroxy-cinnamic acid	O	(-)	homovanillic acid<br>vanillin	C9H10O4<br>C8H8O3	14.22<br>16.13
**52**	15.10	C11H12O4	208.0729	-3.2	S		dimethoxy-hydroxy-cinnamaldehyde<br>dimethoxy-cinnamic acid	O	(-)	sinapaldehyde<br>3,4-dimethoxycinnamic acid	C11H12O4<br>C11H12O4	21.16<br>23.57
**53**	15.14	C8H8O3	152.0463	-6.6	S		hydroxy-methoxybenzaldehyde /<br>vanillin isomer	O	(-) / **(+)**	vanillin<br>syringaldehyde	C8H8O3<br>C9H10O4	16.13<br>17.47
**54**	15.22	C7H6O2	f122.0357	-8.8	S		hydroxy-benzaldehyde	O	(-)	vanillin<br>benzoic acid	C8H8O3<br>C7H6O2	16.13<br>19.41
**55**	15.58	C9H8O2	148.0516	-5.5	S		cinnamic acid	O	(+)	p-coumaric acid	C9H8O3	16.94
**56**	15.79	C8H10O3	154.0615	-9.7	S		dimethoxy-phenol	SG	(-)	syringic acid<br>syringaldehyde	C9H10O5<br>C9H10O4	14.07<br>17.47
**57**	15.83	C7H8O3	140.046	-9.6	S		dixydroxy-methoxybenzene<br>(methoxy-catechol)	SG	(-)	vanillin<br>4-methylcatechol	C8H8O3<br>C7H8O2	16.13<br>16.15
**58**	15.86	C9H6O3	162.03	-10.5	S		hydroxycoumarin	C	(-)	umbelliferone<br>4-hydroxycoumarin	C9H6O3<br>C9H6O3	17.77<br>21.55
**59**	15.91	C9H8O3	164.0459	-8.4	S		caffeic aldehyde	O	(+)	caffeic acid	C9H8O4	13.4
**60**	16.13	C8H8O3	152.0461	-8.2	A	vanillin		G	(-) / **(+)**			
**61**	16.15	C7H8O2	124.0502	-18.0	A	4-methylcatechol		O	(-)			

ID	LC-RT (min.)	Molecular Formula	Neutral Mass (Da)	Δ*m* (ppm)	Quantitation with A or S?	Name (if A)	Tentative aromatic structure (if S)	Chemical Class	Ion mode	Surrogate standard(s) used for quantification	Formula of surrogate(s)	LC-RT of surrogate(s)
**62**	16.35	C10H8O3	176.0467	-3.5	S		hydroxy-methylcoumarin	C	(+)	4-methylumbelliferone	C10H8O3	21.45
**63**	16.39	C10H8O4	192.0411	-6.1	S		methoxy-hydroxycoumarin<br>methyl-dihydroxycoumarin	C	(+)	scopoletin	C10H8O4	18.29
**64**	16.66	C10H10O2	162.0670	-6.5	A	1-phenyl-1,3-butanedione		O	(+)			
**65**	16.77	C10H10O4	194.0568	-5.9	S		hydroxycinnamic acid	O	(+)	trans-ferulic acid	C10H10O4	18.45
**66**	16.77	C6H5NO4	155.0201	-11.3	A	4-nitrocatechol		N	(-)			
**67**	16.79	C10H5O4	189.0209	11.2	S		coumarin-carboxylate	C	(-)	umbelliferone	C9H6O3	17.77
**68**	16.94	C9H8O3	164.0468	-3.4	A	p-coumaric acid		O	(-) / **(+)**			
**69**	17.47	C9H10O4	182.0561	-9.9	A	syringaldehyde		SG	**(-)** / (+)			
**70**	17.69	C10H10O3	178.0616	-8.0	S		methoxycinnamic acid	O	(+)	trans-4-methoxycinnamic acid	C10H10O3	26.57
**71**	17.77	C9H6O3	162.0309	-4.8	A	umbelliferone		C	(+)			
**72**	18.02	C9H10O3	166.0617	-8.0	A	acetovanillone		G	(-) / **(+)**			
**73**	18.29	C10H8O4	192.0418	-2.8	A	scopoletin		C	(-) / **(+)**			
**74**	18.45	C10H10O4	194.0569	-5.2	A	trans-ferulic acid		G	(-) / **(+)**			
**75**	18.65	C11H12O5	224.0676	-3.9	A	sinapinic acid		SG	**(-)** / (+)			
**76**	18.75	C9H10O4	182.058	0.5	A	3,4-dimethoxybenzoic acid		O	**(-)** / (+)			
**77**	18.85	C10H8O4	192.0410	-6.7	S		methoxy-hydroxycoumarin<br>methyl-dihydroxycoumarin	C	(+)	scopoletin	C10H8O4	18.29
**78**	18.87	C10H12O4	196.0725	-5.4	A	acetosyringone		SG	(-) / **(+)**			
**79**	19.04	C11H10O5	222.0528	-0.1	S		hydroxy-dimethoxycoumarin	C	(+)	scopoletin	C10H8O4	18.29
**80**	19.05	C7H5NO5	183.0167	-0.4	A	5-nitrosalicylic acid		N	(-)			
**81**	19.41	C7H6O2	122.0354	-11.3	A	benzoic acid		O	(-)			
**82**	20.31	C8H8O3	152.0466	-4.9	S		methoxybenzoic acid	O	(-)	3-hydroxybenzoic acid<br>4-methoxybenzoic acid	C7H6O3<br>C8H8O3	12.91<br>21.65
**83**	20.59	C12H12O3	204.0772	-6.7	S		hydroxy-trimethylcoumarin	C	(-) / **(+)**	7-hydroxy-3,4,8-trimethylcoumarin	C12H12O3	28.15
**84**	20.72	C10H8O3	176.046	-7.6	S		hydroxy-methylcoumarin	C	(-)	4-methylumbelliferone	C10H8O3	21.45
**85**	20.86	C10H10O3	178.0635	2.8	A	coniferaldehyde		G	(-) / **(+)**			
**86**	21.16	C11H12O4	208.073	-2.7	A	sinapaldehyde		SG	**(-)** / (+)			
**87**	21.28	C6H5NO3	139.0263	-4.6	A	4-nitrophenol		N	(-)			
**88**	21.45	C10H8O3	176.0472	-0.8	A	4-methylumbelliferone		C	**(-)** / (+)			
**89**	21.55	C9H6O3	162.0317	0.0	A	4-hydroxycoumarin		C	**(-)** / (+)			
**90**	21.65	C8H8O3	152.0467	-4.2	A	4-methoxybenzoic acid		O	(-)			
**91**	21.65	C7H7NO5	185.0316	-4.5	S		methoxy-nitrocatechol	N	(-)	4-nitroguaiacol<br>2-methyl-4-nitroresorcinol	C7H7NO4<br>C7H7NO4	22.92<br>29.45
**92**	21.68	C7H7NO4	169.0367	-4.8	S		methoxy-nitrophenol<br>hydroxy-methyl-nitrophenol	N	(-)	4-nitroguaiacol<br>2-methyl-4-nitroresorcinol	C7H7NO4<br>C7H7NO4	22.92<br>29.45
**93**	22.52	C11H12O3	192.0777	-4.7	A	vanillylidenacetone		G	(-) / **(+)**			
**94**	22.56	C11H12O4	208.0724	-5.6	S		dimethoxy-hydroxy-cinnamaldehyde<br>dimethoxy-cinnamic acid	O	(-) / **(+)**	sinapaldehyde<br>3,4-dimethoxycinnamic acid	C11H12O4<br>C11H12O4	21.16<br>23.57
**95**	22.92	C7H7NO4	169.0371	-2.4	A	4-nitroguaiacol		N	(-)			
**96**	23.05	C11H10O4	206.0561	-8.7	S		dimethoxycoumarin	C	(+)	5,7-dimethoxycoumarin	C11H10O4	31.0
**97**	23.57	C11H12O4	208.0732	-1.7	A	3,4-dimethoxycinnamic acid		O	**(-)** / (+)			
**98**	23.87	C11H12O5	224.0681	-1.7	S		dimethoxy-hydroxycinnamic acid	O	(-)	sinapinic acid	C11H12O5	18.65
**99**	24.08	C8H9NO5	199.0483	1.1	S		dimethoxy-nitrophenol,<br>e.g., nitrosyringol	N	(-)	4-nitroguaiacol	C7H7NO4	22.92
**100**	24.75	C7H7NO4	169.0374	-0.6	S		methoxy-nitrophenol<br>methyl-nitrocatechol	N	(-)	4-nitroguaiacol<br>2-methyl-4-nitroresorcinol	C7H7NO4<br>C7H7NO4	22.92<br>29.45
**101**	24.79	C12H12O4	220.0716	-8.9	S		methyl-dimethoxycoumarin	C	(+)	5,7-dimethoxycoumarin<br>7-ethoxycoumarin	C11H10O4<br>C11H10O3	31.0<br>31.3
**102**	24.80	C14H14O4	246.0896	1.6	A	nodakenetin		C	(-)			

ID	LC-RT (min.)	Molecular Formula	Neutral Mass (Da)	Δm (ppm)	Quantitation with A or S?	Name (if A)	Tentative aromatic structure (if S)	Chemical Class	Ion mode	Surrogate standard(s) used for quantification	Formula of surrogate(s)	LC-RT of surrogate(s)
**103**	25.19	C15H14O5	274.0844	1.0	S		flavanol	F	(-)	kaempferol<br>diosmetin	C15H10O6<br>C16H12O6	29.75<br>30.05
**104**	25.20	C18H20O7	348.1212	0.8	S		chalcone derivative	F	**(-)** / (+)	flavonoids average response		
**105**	25.61	C17H18O6	318.1114	3.3	S		pyranone, e.g., agarotetrol, or flavonoid	F	**(-)** / (+)	flavonoids average response		
**106**	25.61	C9H9NO4	195.0529	-1.3	S		methyl-nitrobenzoate derivative<br>dimethylnitrobenzoic acid<br>hydroxy-dimethoxy phenyl cyanate	N	(-)	4-nitroguaiacol<br>4-nitro-o-cresol<br>2-methyl-4-nitroresorcinol	C7H7NO4<br>C7H7NO3<br>C7H7NO4	22.92<br>27.99<br>29.45
**107**	25.80	C15H14O6	290.081	6.8	S		flavanol, e.g., catechin	F	(-)	kaempferol<br>diosmetin	C15H10O6<br>C16H12O6	29.75<br>30.05
**108**	25.80	C16H16O5	288.1004	2.2	S		dihydrochalcone<br>hydroxy-naphthoquinone	F	**(-)** / (+)	flavonoids average response		
**109**	25.84	C10H8O3	176.0464	-5.1	A	7-methoxycoumarin		C	(+)			
**110**	26.14	C8H7NO3	165.0428	1.2	S		nitroacetophenone, methyl-nitro-benzaldehyde, nitro-vinylphenol	N	(-)	4-nitroguaiacol<br>4-nitro-o-cresol	C7H7NO4<br>C7H7NO3	22.92<br>27.99
**111**	26.45	C12H10O2	186.0665	-8.6	S		naphthaleneacetic acid<br>biphenol	O	(-)	trans-4-methoxycinnamic acid	C10H10O3	26.57
**112**	26.57	C10H10O3	178.0628	-1.1	A	trans-4-methoxycinnamic acid		O	**(-)** / (+)			
**113**	26.69	C17H16O5	300.1004	2.1	S		phenanthrenoid, dihydroxy-dimethoxychalcone / flavonoid	F	**(-)** / (+)	flavonoids average response		
**114**	26.90	C15H12O4	256.073	-2.2	S		stilbenoid<br>dihydroxy-flavanone	S-F	**(-)** / (+)	stilbenes-flavonoids average response		
**115**	27.57	C11H12O4	208.0734	-0.8	S		dimethoxycinnamic acid	O	(-)	3,4-dimethoxycinnamic acid	C11H12O4	23.57
**116**	27.67	C9H9NO4	195.0539	3.8	S		methyl-nitrobenzoate derivative<br>dimethylnitrobenzoic acid<br>hydroxy-dimethoxy phenyl cyanate	N	(-)	4-nitroguaiacol<br>4-nitro-o-cresol<br>2-methyl-4-nitroresorcinol	C7H7NO4<br>C7H7NO3<br>C7H7NO4	22.92<br>27.99<br>29.45
**117**	27.99	C7H7NO3	153.043	2.7	A	4-nitro-o-cresol		N	(-)			
**118**	28.06	C16H14O3	254.0960	6.7	S		hydroxy-methoxychalcone	F	(+)	flavonoids average response		
**119**	28.15	C12H12O3	204.0786	-0.2	A	7-hydroxy-3,4,8-trimethylcoumarin		C	**(-)** / (+)			
**120**	28.43	C9H9NO4	195.0531	-0.3	S		methyl-nitrobenzoate derivative<br>dimethylnitrobenzoic acid<br>hydroxy-dimethoxy phenyl cyanate	N	(-)	4-nitroguaiacol<br>4-nitro-o-cresol<br>2-methyl-4-nitroresorcinol	C7H7NO4<br>C7H7NO3<br>C7H7NO4	22.92<br>27.99<br>29.45
**121**	29.06	C18H20O6	332.1272	3.6	S		dihydroxy-tetramethoxystilbene	S	**(-)** / (+)	combretastatin A1	C18H20O6	33.35
**122**	29.29	C17H18O5	302.1164	3.2	S		dihydroxy-trimethoxystilbene	S	**(-)** / (+)	stilbenes average response		
**123**	29.45	C7H7NO4	169.0375	-0.1	A	2-methyl-4-nitroresorcinol		N	(-)			
**124**	29.71	C17H14O7	330.0733	-2.0	S		trihydroxy-dimethoxyflavone or flavonol	F	(-) / **(+)**	kaempferol<br>diosmetin	C15H10O6<br>C16H12O6	
**125**	29.75	C15H10O6	286.0477	-0.1	A	kaempferol		F	**(-)** / (+)			
**126**	29.83	C11H10O2	174.069	5.3	S		methoxy-naphthol<br>methyl-dihydroxynaphthalene	F	(-)	kaempferol<br>diosmetin	C15H10O6<br>C16H12O6	29.75<br>30.05
**127**	30.05	C16H12O6	300.0634	0.0	A	diosmetin		F	**(-)** / (+)			
**128**	30.07	C8H9NO4	183.0533	0.8	S		dimethoxy-nitrobenzene<br>methoxy-methyl-nitrophenol	N	(-)	4-nitro-o-cresol<br>2-methyl-4-nitroresorcinol	C7H7NO3<br>C7H7NO4	27.99<br>29.45
**129**	30.21	C14H12O3	228.0778	-3.7	S		trihydroxy-stilbene,<br>e.g., resveratrol	S	(-)	stilbenes average response		
**130**	31.10	C17H16O5	300.0992	-2.0	S		phenanthrenoid or dihydroxy-dimethoxychalcone / flavonoid	F	(-) / **(+)**	flavonoids average response		
**131**	32.01	C16H14O4	270.091	6.6	S		phenanthrenoid, chalcone or flavanone	F	**(-)** / (+)	flavonoids average response		
**132**	32.46	C16H18O4	274.1209	1.5	S		dimethoxy di-p-cresol, gigantol,<br>dihydroxy-dimethoxy-dihydrostilbene	S-F	(-)	stilbenes-flavonoids average response		

ID	LC-RT (min.)	Molecular Formula	Neutral Mass (Da)	Δ*m* (ppm)	Quantitation with A or S?	Name (if A)	Tentative aromatic structure (if S)	Chemical Class	Ion mode	Surrogate standard(s) used for quantification	Formula of surrogate(s)	LC-RT of surrogate(s)
**133**	33.19	C14H12O2	212.0830	-3.4	S		dihydroxystilbene	S	(-)	stilbenes average response		
**134**	33.21	C17H18O5	302.1163	3.0	S		dihydroxy-trimethoxystilbene	S	(-)	stilbenes average response		
**135**	33.22	C15H14O5	274.0849	2.9	S		flavonoid (dihydrochalcone or flavanol), e.g., phloretin	F	(-)	flavonoids average response		
**136**	33.35	C18H20O6	332.1260	0.0	A	combretastatin A1		S	**(-)** / (+)			
**137**	33.67	C18H16O6	328.0959	3.6	S		hydroxy-trimethoxyflavone	F	(-)	flavonoids average response		
**138**	33.67	C14H12O4	244.0744	3.5	S		tetrahydroxy-stilbene	S	(-)	stilbenes average response		
**139**	33.71	C18H18O6	330.1092	-3.5	S		dihydroxy-trimethoxychalcone	F	(-)	flavonoids average response		
**140**	33.82	C19H16O6	340.0982	10.2	S		didemethyl curcumin	F	(-)	flavonoids average response		
**141**	33.83	C12H12O3	204.0784	-1.4	S		ethyl-hydroxy-methylcoumarin<br>hydroxy-methoxy-prenylcoumarin<br>hydroxy-trimethylcoumarin	C	(-)	7-hydroxy-3,4,8-trimethylcoumarin	C12H12O3	28.15
**142**	34.05	C19H20O5	328.1328	5.3	S		flavonoid or hirsutenone	F	(-)	flavonoids average response		
**143**	34.07	C16H14O4	270.0912	7.4	S		phenanthrenoid, chalcone or flavanone	F	(-)	flavonoids average response		
**144**	34.23	C17H16O4	284.1048	-0.4	S		hydroxy-dimethoxychalcone	F	(+)	flavokawain B	C17H16O4	50.1
**145**	34.27	C10H7NO3	189.043	2.1	A	4-nitro-1-naphthol		N	(-)			
**146**	34.69	C20H18O5	338.1171	5.0	S		curcuminoid or flavonoid	F	**(-)** / (+)	flavonoids average response		
**147**	35.01	C20H20O6	356.1285	7.0	S		coniferyl ferulate<br>tetrahydroxyflavanone	F	(-)	flavonoids average response		
**148**	35.51	C15H16O4	260.1061	4.8	S		ethyl-hydroxy-methylcoumarin<br>hydroxy-methoxy-prenylcoumarin	C	(-)	7-hydroxy-3,4,8-trimethylcoumarin	C12H12O3	28.15
**149**	35.56	C20H20O6	356.1261	0.3	S		coniferyl ferulate<br>tetrahydroxyflavanone	F	(+)	flavonoids average response		
**150**	35.97	C21H22O8	402.1325	2.6	S		hexamethoxyflavone	F	(+)	flavonoids average response		
**151**	36.05	C16H16O4	272.1049	0.1	A	3'-hydroxypterostilbene		S	**(-)** / (+)			
**152**	36.29	C15H10O4	254.0588	3.5	S		dihydroxyflavone, dihydroxyanthraquinone	F	(-)	flavonoids average response		
**153**	36.52	C18H16O4	296.1056	2.5	S		flavonoid, e.g., desmosflavone	F	(-)	flavonoids average response		
**154**	36.66	C17H14O6	314.0799	2.7	S		phenanthrenoid<br>dihydroxy-dimethoxyflavone	F	(-)	flavonoids average response		
**155**	37.16	C15H12O4	256.0718	-6.9	S		stilbenoid or dihydroxy-flavanone	S-F	(-) / **(+)**	stilbenes-flavonoids average response		
**156**	37.38	C21H20O7	384.1226	4.3	S		flavonoid	F	(+)	flavonoids average response		
**157**	37.65	C18H16O7	344.0911	4.3	S		dihydroxy-trimethoxyflavone	F	(-)	flavonoids average response		
**158**	37.91	C21H22O6	370.1397	-5.3	S		chalcone, dihydroxycurcumin, or flavanone	F	(+)	flavonoids average response		
**159**	38.44	C19H18O6	342.1138	10.1	S		stilbenoid	S	(-)	stilbenes average response		
**160**	38.44	C21H22O6	370.1406	-2.8	S		chalcone, dihydroxycurcumin, or flavanone	F	(+)	flavonoids average response		
**161**	38.83	C20H20O5	340.1302	-2.6	S		chalcone or flavanone	F	(+)	flavonoids average response		
**162**	39.43	C18H16O5	312.1021	7.4	S		dimethoxy-hydroxy-methylflavone<br>hydroxy-trimethoxychalcone	F	(-)	flavonoids average response		
**163**	39.61	C19H18O5	326.1144	-3.0	S		flavonoid	F	(+)	flavonoids average response		
**164**	39.82	C16H18O4	274.1217	4.3	S		dimethoxy di-p-cresol, gigantol, dihydroxy-dimethoxy-dihydrostilbene	S-F	**(-)** / (+)	stilbenes-flavonoids average response		
**165**	39.87	C14H16O3	232.1084	-6.7	S		coumarin	C	(+)	ethyl-dimethoxycoumarin	C13H14O4	38.1
**166**	39.90	C21H20O6	368.1249	-3.1	S		curcumin	F	(+)	flavonoids average response		
**167**	40.59	C18H12O6	324.0629	-1.7	S		flavonoid	F	(+)	flavonoids average response		
**168**	40.85	C20H18O5	338.1177	6.7	S		curcuminoid or flavonoid	F	(-)	flavonoids average response		
**169**	40.95	C18H16O5	312.0981	-5.4	A	6,2′,4′-trimethoxyflavone		F	(+)			
**170**	40.98	C18H18O5	314.1176	6.9	S		hydroxy-trimethoxychalcone	F	(-)	flavokawain C	C17H16O5	41.05
**171**	41.05	C17H16O5	300.0998	0.1	A	flavokawain C		F	**(-)** / (+)			
**172**	41.26	C17H16O4	284.1065	5.8	S		hydroxy-dimethoxychalcone	F	(-)	flavokawain C	C17H16O5	41.05
**173**	42.03	C16H14O4	270.0904	4.4	S		phenanthrenoid, chalcone or flavanone	F	(-)	flavonoids average response		

ID	LC-RT (min.)	Molecular Formula	Neutral Mass (Da)	Δ*m* (ppm)	Quantitation with A or S?	Name (if A)	Tentative aromatic structure (if S)	Chemical Class	Ion mode	Surrogate standard(s) used for quantification	Formula of surrogate(s)	LC-RT of surrogate(s)
**174**	42.53	C18H16O4	296.1071	7.6	S		flavonoid, e.g., desmosflavone	F	(-)	flavonoids average response		
**175**	42.81	C16H16O4	272.1057	3.1	S		dimethoxy-dihydroxystilbene	S	(-)	3'-hydroxypterostilbene	C16H16O4	36.05
**176**	43.20	C18H18O5	314.1162	2.5	S		hydroxy-trimethoxychalcone	F	(+)	flavokawain A	C18H18O5	49.7
**177**	44.09	C20H16O7	368.0884	-3.3	S		flavonoid	F	(+)	flavonoids average response		
**178**	45.96	C19H20O5	328.1309	-6.7	S		tetramethoxychalcone	F	(+)	tetramethoxychalcone	C19H20O5	44.1
**179**	46.93	C18H18O4	298.1203	-0.6	S		trimethoxychalcone	F	(+)	2,4,4'-trimethoxychalcone	C18H18O4	45.2
**180**	49.66	C19H20O4	312.1344	-5.8	S		methyl-trimethoxychalcone isomer	F	(+)	methyl-trimethoxychalcone	C19H20O4	48.7
**181**	49.78	C21H22O5	354.1455	-3.5	S		chalcone	F	(+)	flavonoids average response		
**182**	50.37	C20H20O4	324.1348	-4.3	S		chalcone	F	(+)	flavonoids average response		

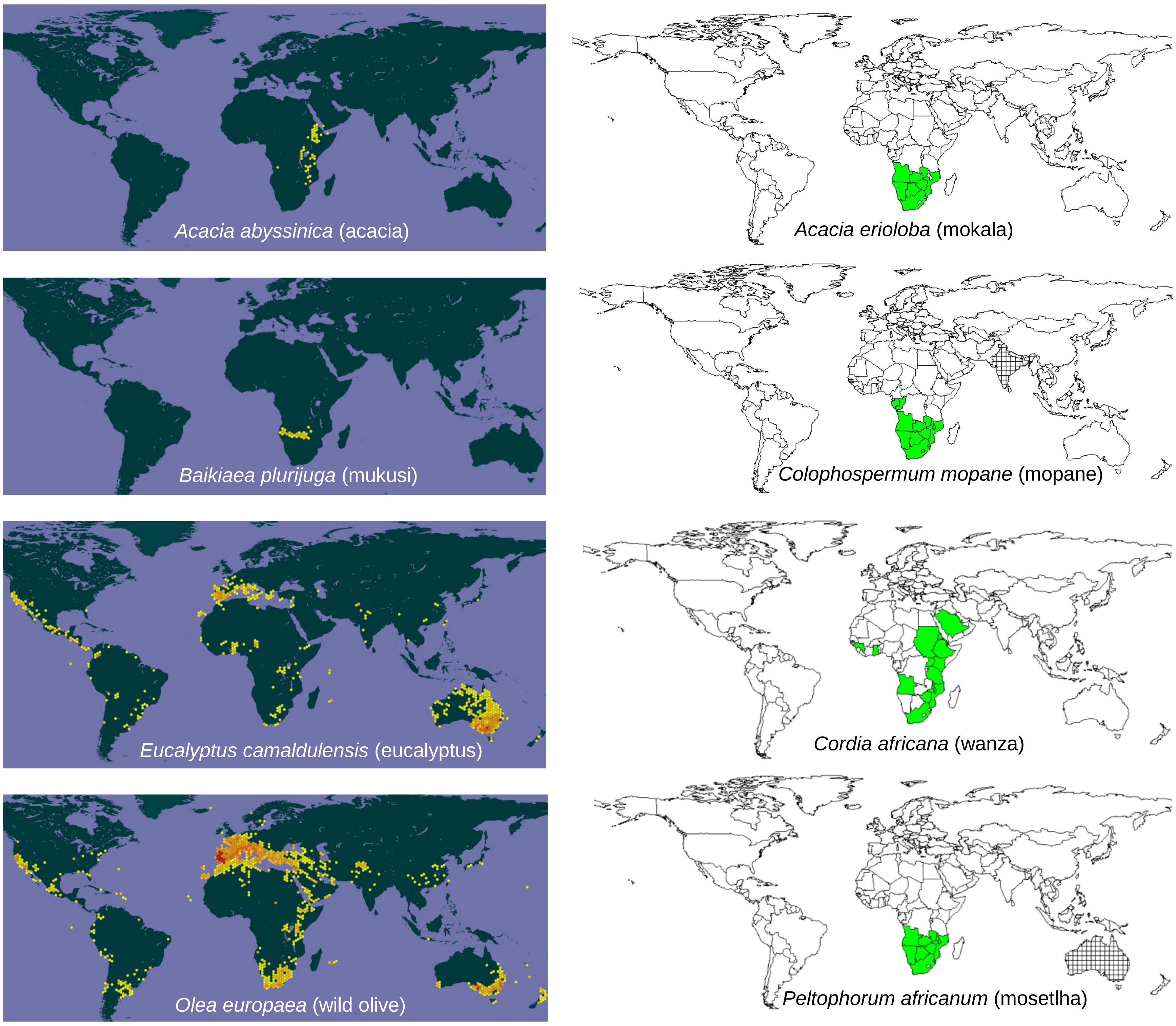

Figure S1. Geographical distributions of African hardwood biomass fuels combusted in this study: The left-side maps display georeferenced records sourced from the Global Biodiversity Information Facility,[2] while the right-side maps present species distributions; native range countries are highlighted in green, with grid overlays marking exotic ranges, as taken from the Agroforestry Database 4.0.[3] In this study, biomass fuels, inclusive of hardwoods, originated from Botswana. Exceptions include acacia, eucalyptus, wanza, wild olive, and dried cow-dung cakes, which were procured directly from Ethiopia.

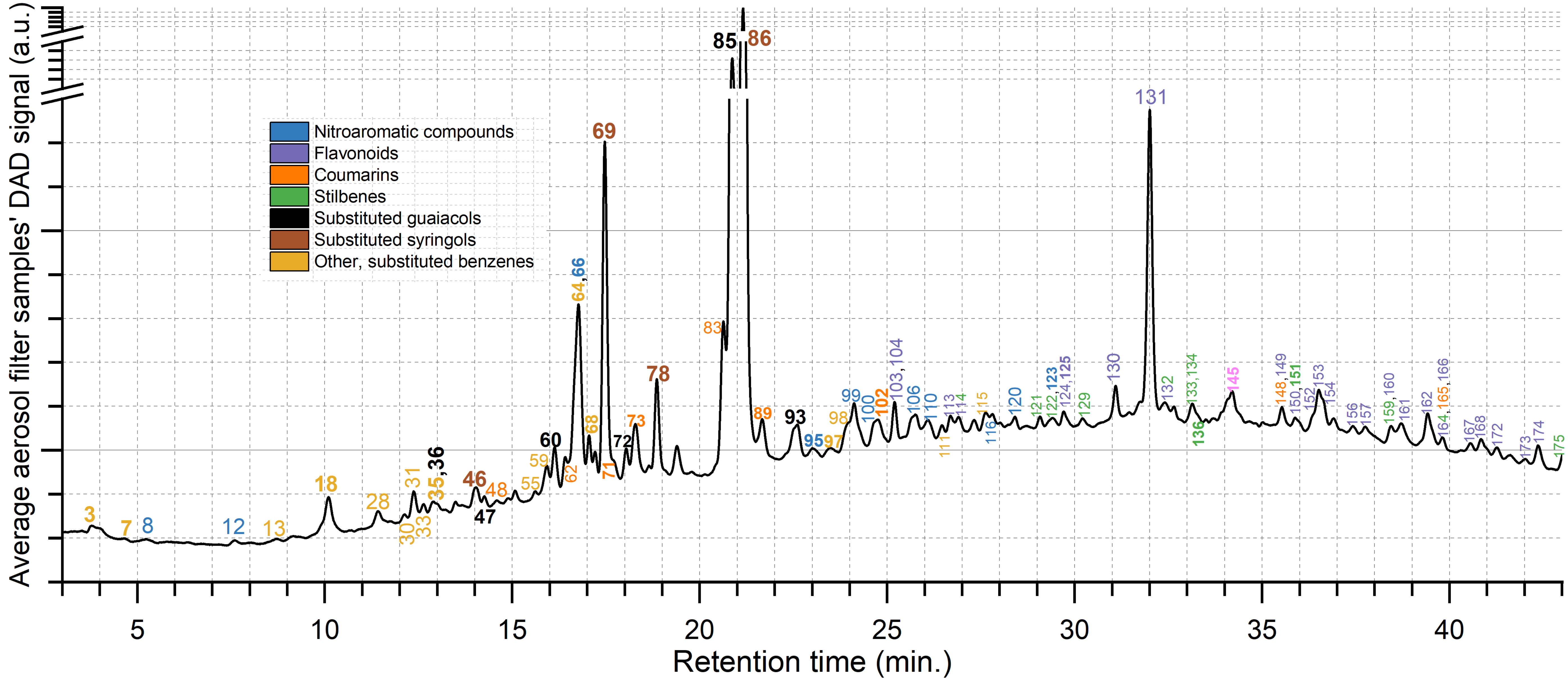

Figure S2. Averaged extracted wavelength chromatogram (integrated from 305-395 nm; reference: 690-710 nm) from 35 aerosol filter sample extracts, corrected by subtracting the mean of blank filter samples. Before averaging and correction, each sample chromatogram was normalized so that its values sum to 1. The chromatogram is based on DAD measurements and spans RTs from 3-43 min. Major peak IDs are shown and color-coded by chemical class: substituted guaiacols (black), substituted syringols (dark brown), other substituted benzenes (gold), nitro-aromatic compounds (deep blue), coumarins (dark orange), stilbenes (green), and flavonoids (violet). Peaks matching an external authentic standard (same molecular formula and retention time) are emphasized in bold. The figure implies that most RPLC-separated BrC species have been identified with ESI, hinting at a potential low abundance of (soluble) polycyclic aromatic hydrocarbons (PAHs) in these samples. Refer to Table S4 for detailed peak information.

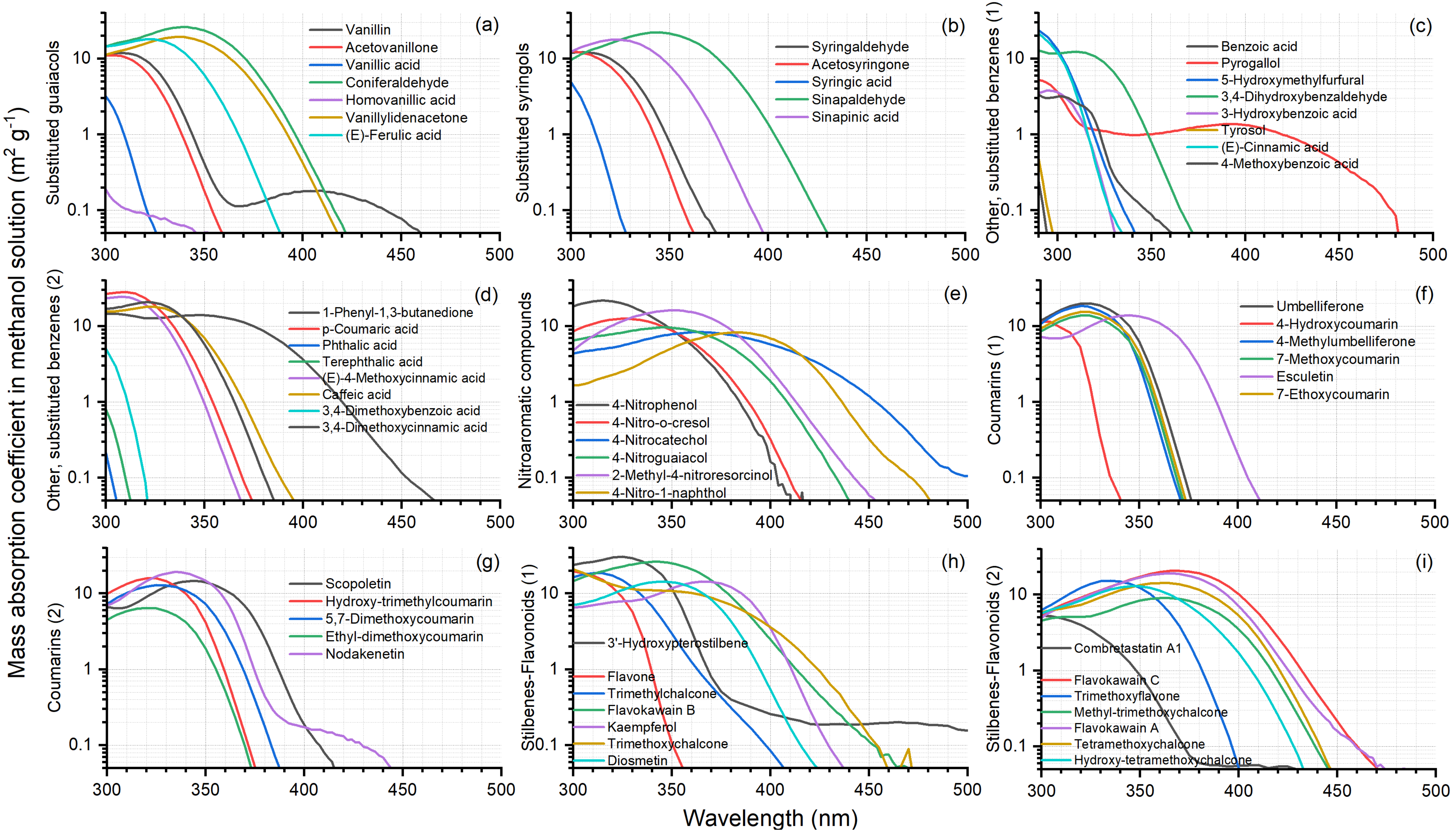

Figure S3. Mass absorption coefficient spectra (within the Rayleigh regime) spanning 290- (or 300)-500 nm, representing individual methanol-extracted, RPLC-separated BrC molecules (authentic standards) under neutral conditions, categorized by chemical class. The spectra were derived from DAD analysis (Text S4) of mixtures containing 10 ppm of each authentic standard (distinct groups w/o isomers), and include only those standards that were identified via ESI and employed for quantifying BrC species in the aerosol filter samples.

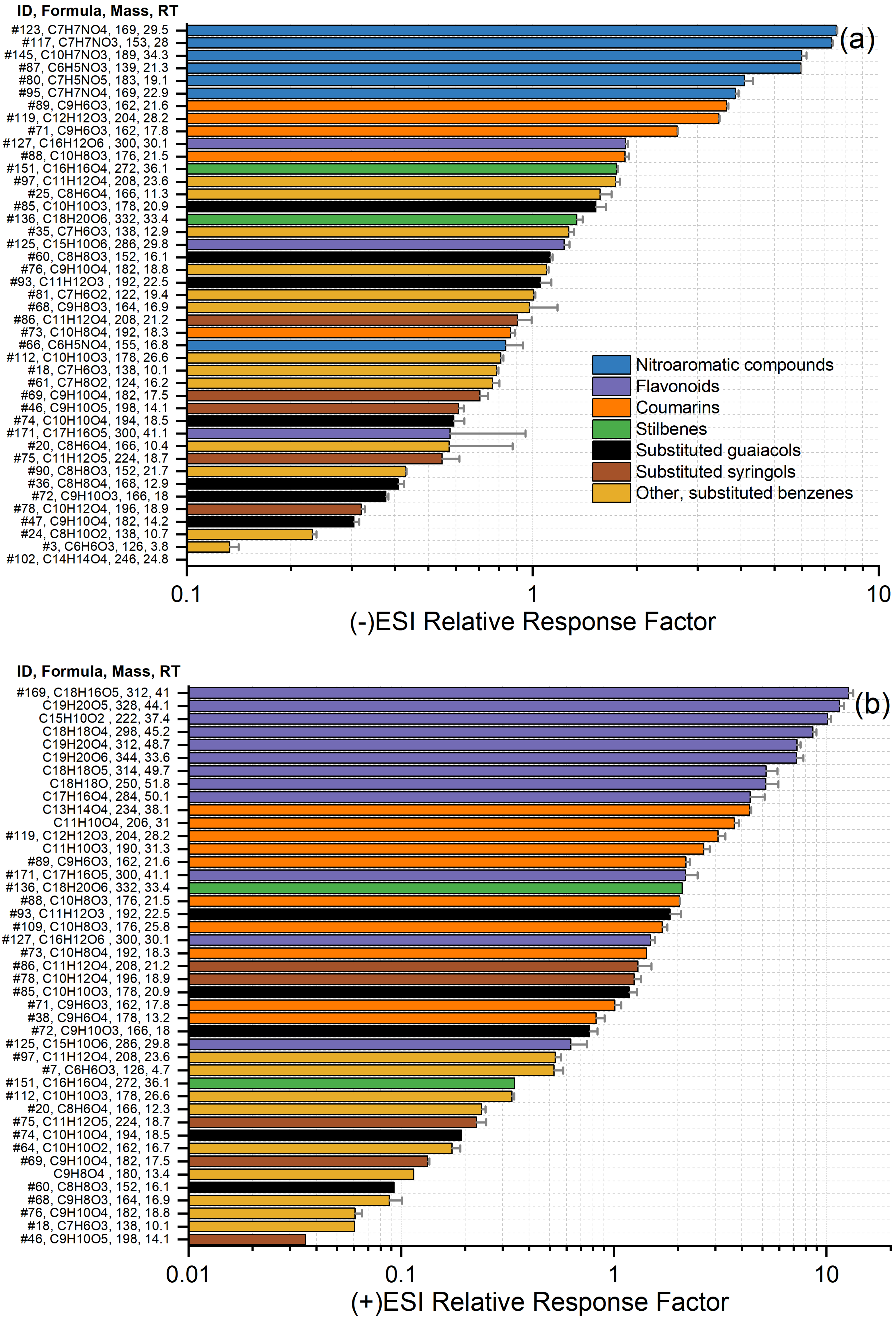

Figure S4. Relative response factors (RRFs) in (a) (-)ESI and (b) (+)ESI for BrC authentic standards, where RRF = 1 signifies the geometric mean (RRF ≈ 1 for phthalic acid in negative ESI and umbelliferone in positive ESI). The geometric mean RF for (-)ESI ($4{\cdot}10^6$) was approximately six times higher than for (+)ESI ($7{\cdot}10^5$). The standards are arranged in descending order of RRF (see Table S2) and color-coded based on their respective chemical class. The error bars represent the uncertainty in the slope of the calibration curve within the linear-range, as obtained from measurements at the beginning and end of the measurements set for each ion mode (typical uncertainty of 10% during a 2-day period per mode). IDs of BrC species found in aerosol filter samples are associated with the RPLC-measured RTs provided in Table S4. Standards without an ID (i.e., not detected in the aerosol filter extracts) were still used as surrogates to quantify BrC species when a matching authentic standard was not available.

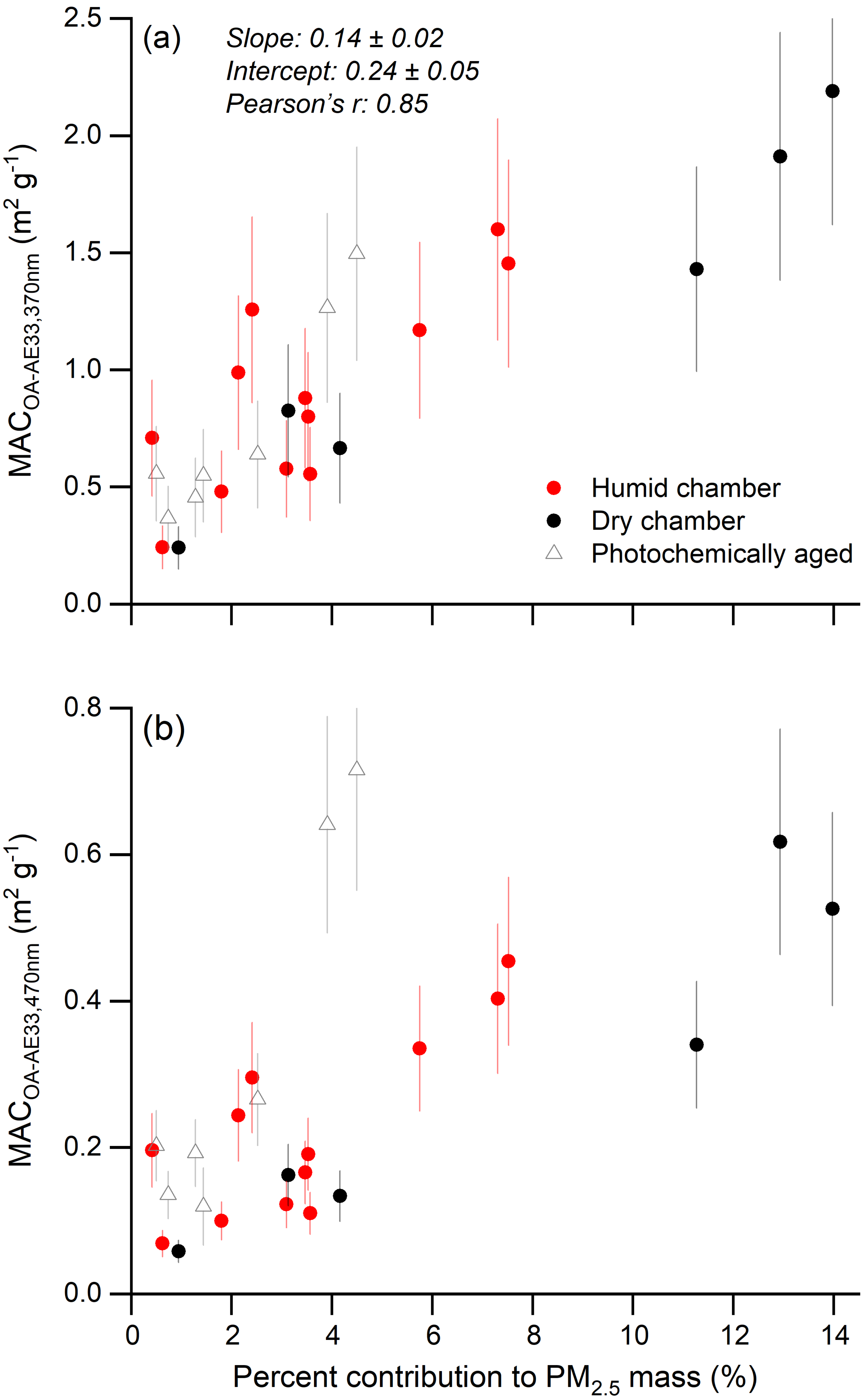

Figure S5. Scatter plot of the BrC mass closure result (Fig. 4b) and the absorptivity of OA (Fig. 1 and Table S1) at 370 nm (a), and 470 nm (b). The data represented is for chamber-aerosol filter samples from primary/dark emissions (circles) in the dry (black) or humid (red) chamber, and photochemically aged humid chamber (triangles) emissions that underwent molecular-level BrC composition analysis (*N* = 25). The error bars account for variations in repeated chamber experiments and the aethalometer calibration uncertainty (relative uncertainties combined in quadrature). The linear fit considers instrumental weighting of errors.

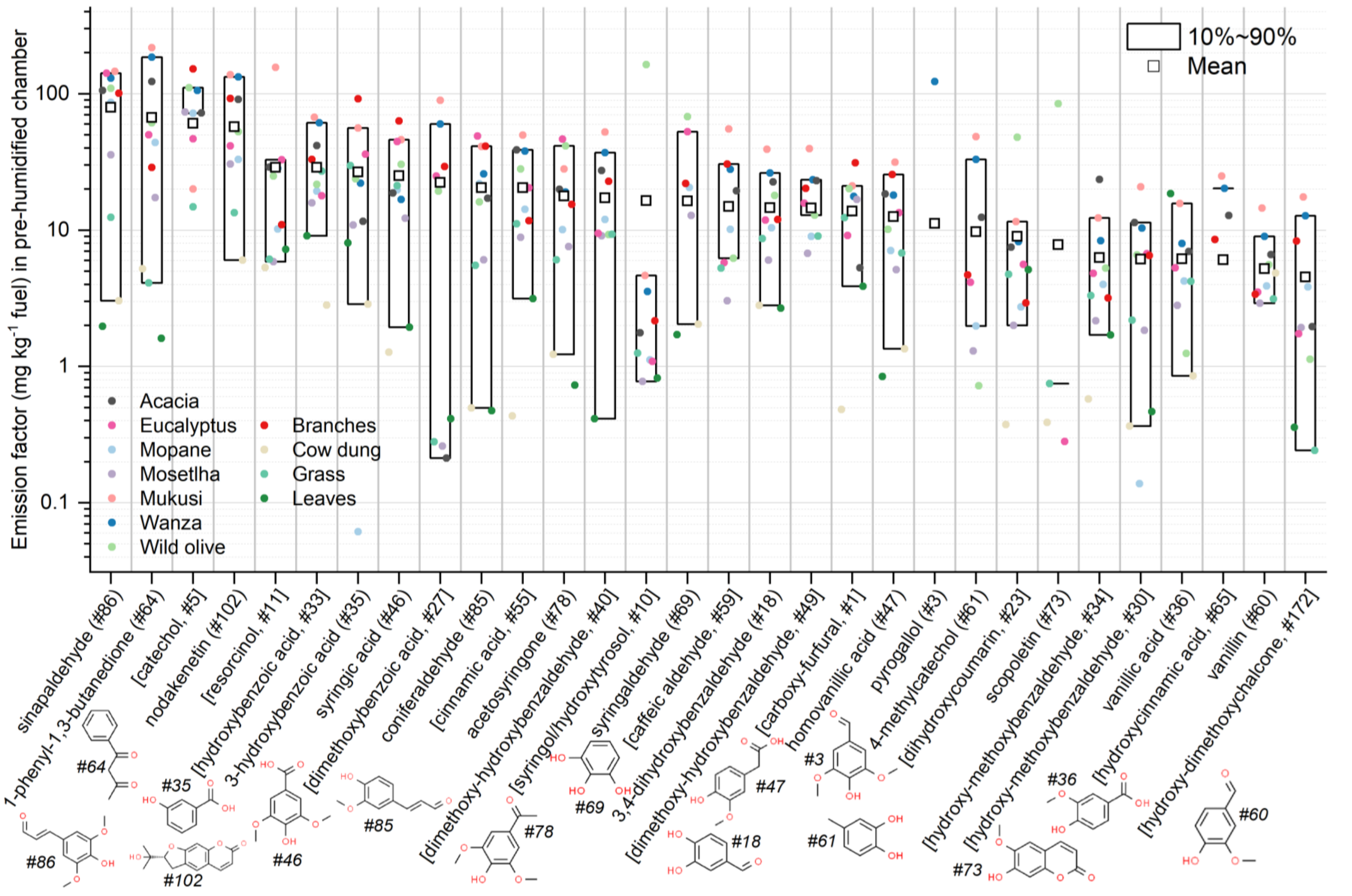

Figure S6. BrC species emission factors (EFs, in mg per kg of fuel) in the pre-humidified chamber, for the top thirty contributors accounting for 16% of all identified BrC species, arranged in descending order based on the mean EFs (squares) across the 11 fuels (colored markers); the color-coding for each fuel is consistent with that used in Fig. 2. Each species is denoted by their ID (see Table S4), with the structures of authentic standards displayed at the bottom. Species that have been quantified using surrogate standards are marked with brackets, indicating their provisional structures. The substantial fuel-to-fuel variability in EFs is emphasized by the wide range of lower-to-upper percentiles.

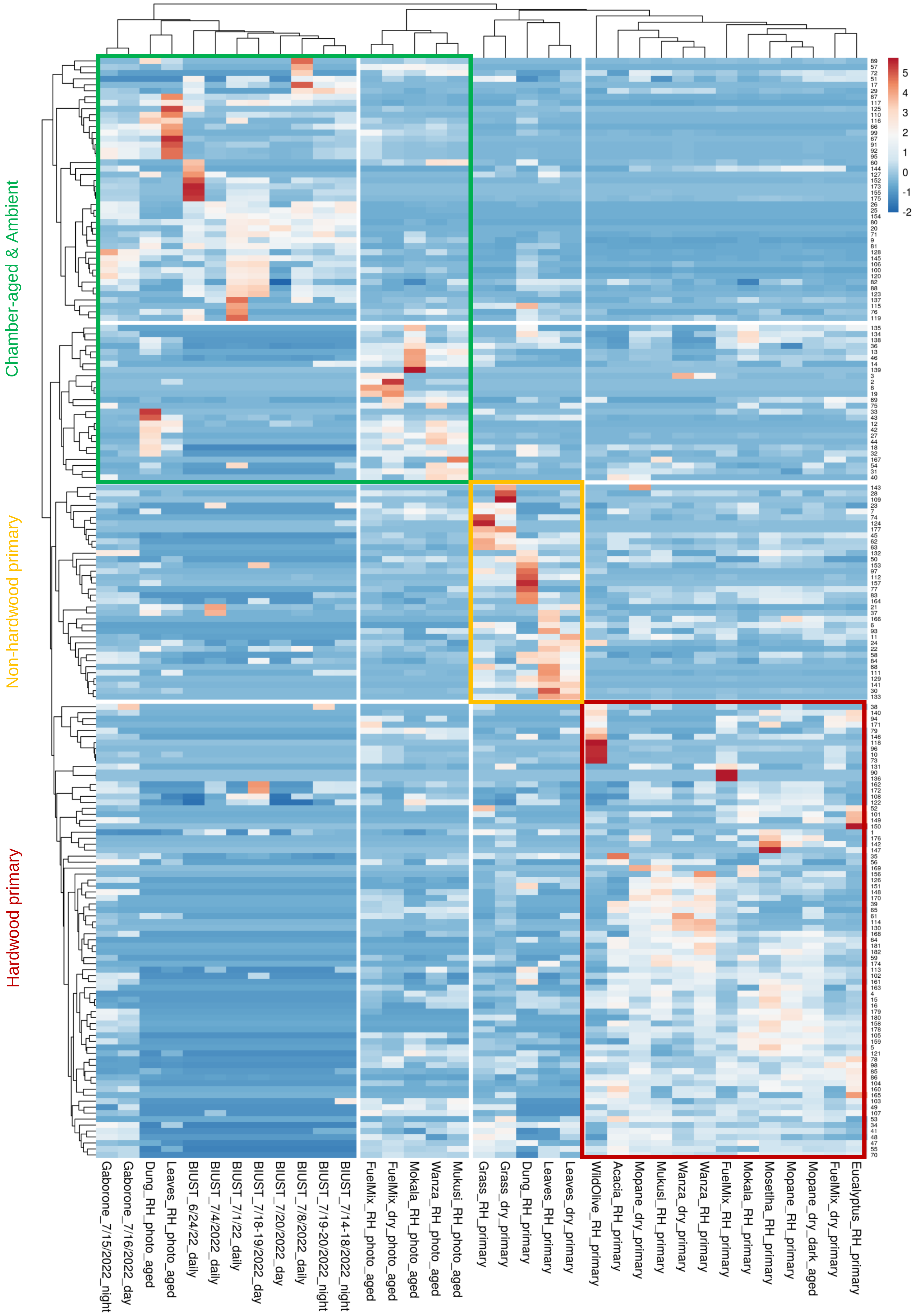

Figure S7. Hierarchical clustering of 182 individual BrC species' fractional contributions to total identified BrC mass within 25 chamber- and 10 ambient-aerosol filter extracts (columns). Rows, denoting compounds (see Table S4 for ID details), are scaled using unit variance. Clustering is based on correlation distance and average linkage for both rows and columns. A color gradient, ranging from dark blue to wine red, represents standardised matrix values. Selected clusters in the matrix are outlined for enhanced clarity and interpretation.

SI References

(1) A selection of African native trees: biology, uses, propagation and restoration techniques. ISBN 978-99944-3-086-4, 621 pages, Addis Ababa, Ethiopia.

(2) GBIF Secretariat (2022). GBIF Backbone Taxonomy. Checklist dataset https://doi.org/10.15468/39omei accessed via GBIF.org on 2023-06-03.

(3) Orwa C, A Mutua, Kindt R, Jamnadass R, S Anthony. 2009 Agroforestree Database: a tree reference and selection guide version 4.0 (http://www.worldagroforestry.org/sites/treedbs/treedatabases.asp).

(4) Kyalangalilwa B., Boatwright J.S., Daru B.H., Maurin O., van der Bank M. (2013). "Phylogenetic position and revised classification of Acacia s.l. (Fabaceae: Mimosoideae) in Africa, including new combinations in Vachellia and Senegalia." *Botanical Journal of the Linnean Society*, 172(4): 500–523.

(5) Orwa C, Mutua A, Kindt R, Jamnadass R, Simons A. 2009. Agroforestree Database: a tree reference and selection guide version 4.0, https://apps.worldagroforestry.org/treedb2/AFTPDFS/Acacia_erioloba.PDF.

(6) Orwa C, Mutua A, Kindt R, Jamnadass R, Simons A. 2009. Agroforestree Database: a tree reference and selection guide version 4.0, https://apps.worldagroforestry.org/treedb2/AFTPDFS/Baikiaea_plurijuga.PDF.

(7) Palmer, E., Pitman, N. (1972). "Trees of Southern Africa, covering all known indigenous species in the Republic of South Africa, South-West Africa, Botswana, Lesotho & Swaziland." A.A. Balkema, Cape Town.

(8) Orwa C, Mutua A, Kindt R, Jamnadass R, Simons A. 2009. Agroforestree Database: a tree reference and selection guide version 4.0, http://apps.worldagroforestry.org/treedb2/AFTPDFS/Colophospermum_mopane.PDF.

(9) Timberlake JR, 1995. Colophospermum mopane, annotated bibliography and review. The Zimbabwe Bulletin of Forestry Research, No. 11: 49 pp.

(10) Orwa C, Mutua A, Kindt R, Jamnadass R, Simons A. 2009. Agroforestree Database: a tree reference and selection guide version 4.0, https://apps.worldagroforestry.org/treedb2/AFTPDFS/Cordia_africana.PDF.

(11) Bekele-Tesemma, A. (2007). "Useful trees and shrubs of Ethiopia: identification, propagation and management for 17 agroclimatic zones." RELMA in ICRAF Project.

(12) Orwa C, Mutua A, Kindt R, Jamnadass R, Simons A. 2009. Agroforestree Database: a tree reference and selection guide version 4.0, https://apps.worldagroforestry.org/treedb2/AFTPDFS/Eucalyptus_camaldulensis.PDF.

(13) Brooker, M.I.H., Kleinig, D.A. (1990). "Field Guide to Eucalyptus." Inkata Press, Melbourne.

(14) Orwa C, Mutua A, Kindt R, Jamnadass R, Simons A. 2009. Agroforestree Database: a tree reference and selection guide version 4.0, https://apps.worldagroforestry.org/treedb2/AFTPDFS/Olea_europaea_ssp._africana.PDF.

(15) Green, P.S. (2002). "A revision of Olea L. (Oleaceae)." Kew Bulletin 57(1): 91–140.

(16) Orwa C, Mutua A, Kindt R, Jamnadass R, Simons A. 2009. Agroforestree Database: a tree reference and selection guide version 4.0, https://apps.worldagroforestry.org/treedb2/AFTPDFS/Peltophorum_africanum.PDF.

(17) Coates Palgrave, K. (2002). "Trees of Southern Africa." Struik Publishers.

(18) Sola, P.; Ochieng, C.; Yila, J.; Iiyama, M. Links between energy access and food security in sub Saharan Africa: an exploratory review. *Food Security*, **2016**, *8* (3), 635-642. DOI: 10.1007/s12571-016-0570-1.

(19) World Bank. 2011. Wood-based biomass energy development for sub-Saharan Africa: Issues and approaches. Energy Sector Management Assistance Program (ESMAP); © World Bank, Washington, DC. http://hdl.handle.net/10986/26149.

(20) Archibald, S., Roy, D. P., Wilgen, W. V., & Scholes, R. J. (2009). "What limits fire? An examination of drivers of burnt area in Southern Africa." *Global Change Biology*, 15(3): 613-630.

(21) Smith, D. M.; Fiddler, M. N.; Sexton, K. G.; Bililign, S. Construction and characterization of an indoor smog chamber for measuring the optical and physicochemical properties of aging biomass burning aerosols. *AAQR,* **2019**, *19* (3), 467-483. DOI: 10.4209/aaqr.2018.06.0243.

(22) Pokhrel, R. P.; Gordon, J.; Fiddler, M. N.; Bililign, S. Determination of emission factors of pollutants from biomass burning of African fuels in laboratory measurements. *J. Geophys. Res. Atmos.* **2021**, *126* (20). DOI: 10.1029/2021jd034731.

(23) Pokhrel, R. P.; Gordon, J.; Fiddler, M. N.; Bililign, S. Impact of combustion conditions on physical and morphological properties of biomass burning aerosol. *Aerosol Sci. Technol.* **2020**, *55* (1), 80-91. DOI: 10.1080/02786826.2020.1822512.

(24) Andreae, M. O.; Merlet, P. Emission of trace gases and aerosols from biomass burning. *Global Biogeochemical Cycles*, **2001**, *15* (4), 955-966. DOI: 10.1029/2000gb001382.

(25) Yokelson, R. J.; Susott, R.; Ward, D. E.; Reardon, J.; Griffith, D. W. T. Emissions from smoldering combustion of biomass measured by open-path Fourier transform infrared spectroscopy. *JGR Atmos.*, **1997**, *102* (D15), 18865-18877. DOI: 10.1029/97jd00852.

(26) Urbanski, S. P.; Hao, W. M.; Baker, S. Chapter 4 Chemical Composition of Wildland Fire Emissions. In *Developments in Environmental Science*, Bytnerowicz, A., Arbaugh, M. J., Riebau, A. R., Andersen, C. Eds.; Vol. 8; Elsevier, 2008; pp 79-107.

(27) Akagi, S. K.; Craven, J. S.; Taylor, J. W.; McMeeking, G. R.; Yokelson, R. J.; Burling, I. R.; Urbanski, S. P.; Wold, C. E.; Seinfeld, J. H.; Coe, H.; Alvarado, M. J.; Weise, D. R. Evolution of trace gases and particles emitted by a chaparral fire in California. *Atmos. Chem. Phys.*, **2012**, *12* (3), 1397-1421. DOI: 10.5194/acp-12-1397-2012.

(28) Donahue, N. M., Robinson, A. L., Stanier, C. O., & Pandis, S. N. (2006). Coupled partitioning, dilution, and chemical aging of semivolatile organics. *Environ. Sci. Technol.*, 40(8), 2635-2643.

(29) Reid, J. S., Koppmann, R., Eck, T. F., and Eleuterio, D. P.: A review of biomass burning emissions part II: intensive physical properties of biomass burning particles, *Atmos. Chem. Phys.*, 5, 799–825, https://doi.org/10.5194/acp-5-799-2005, 2005.

(30) Holder, A. L.; Hagler, G. S. W.; Aurell, J.; Hays, M. D.; Gullett, B. K. Particulate matter and black carbon optical properties and emission factors from prescribed fires in the southeastern United States. *JGR Atmos.*, **2016**, *121* (7), 3465-3483. DOI: 10.1002/2015jd024321.

(31) Pankow, J. F. (1994). An absorption model of the gas/aerosol partitioning involved in the formation of secondary organic aerosol. *Atmos. Environ.*, 28(2), 189-193.

(32) Zhang, X.; Lin, Y.-H.; Surratt, J. D.; Zotter, P.; Prévôt, A. S. H.; Weber, R. J. Light-absorbing soluble organic aerosol in Los Angeles and Atlanta: a contrast in secondary organic aerosol. *Geophys. Res. Lett.*, **2011**, *38* (21). DOI: 10.1029/2011gl049385.

(33) Zhang, X.; Lin, Y. H.; Surratt, J. D.; Weber, R. J. Sources, composition and absorption Ångström exponent of light-absorbing organic components in aerosol extracts from the Los Angeles Basin. *Environ. Sci. Technol.*, **2013**, *47* (8), 3685-3693. DOI: 10.1021/es305047b.

(34) Hettiyadura, A. P. S.; Garcia, V.; Li, C.; West, C. P.; Tomlin, J.; He, Q.; Rudich, Y.; Laskin, A. Chemical composition and molecular-specific optical properties of atmospheric brown carbon associated with biomass burning. *Environ. Sci. Technol.* **2021**, *55* (4), 2511-2521. DOI: 10.1021/acs.est.0c05883.

(35) John Wiley & Sons, Inc. SpectraBase; https://spectrabase.com/ (accessed 9/22/2023).

(36) van Outersterp, R. E.; Martens, J.; Berden, G.; Koppen, V.; Cuyckens, F.; Oomens, J. Mass spectrometry-based identification of ortho-, meta- and para-isomers using infrared ion spectroscopy. *Analyst.* **2020**, *145* (18), 6162-6170. DOI: 10.1039/d0an01119c.

(37) Huang, R.-J.; Yang, L.; Shen, J.; Yuan, W.; Gong, Y.; Ni, H.; Duan, J.; Yan, J.; Huang, H.; You, Q.; Li, Y. J. Chromophoric fingerprinting of brown carbon from residential biomass burning. *Environ. Sci. Technol. Lett.* **2021**, *9* (2), 102-111. DOI: 10.1021/acs.estlett.1c00837.

(38) Romonosky, D. E.; Laskin, A.; Laskin, J.; Nizkorodov, S. A. High-resolution mass spectrometry and molecular characterization of aqueous photochemistry products of common types of secondary organic aerosols. *J. Phys. Chem. A.* **2015**, *119* (11), 2594-2606. DOI: 10.1021/jp509476r.

(39) Chen, K.; Raeofy, N.; Lum, M.; Mayorga, R.; Woods, M.; Bahreini, R.; Zhang, H.; Lin, Y.-H. Solvent effects on chemical composition and optical properties of extracted secondary brown carbon constituents. *Aerosol Sci. Technol.* **2022**, *56* (10), 917-930. DOI: 10.1080/02786826.2022.2100734.

(40) Miljevic, B.; Hedayat, F.; Stevanovic, S.; Fairfull-Smith, K. E.; Bottle, S. E.; Ristovski, Z. D. To sonicate or not to sonicate PM filters: reactive oxygen species generation upon ultrasonic irradiation. *Aerosol Sci. Technol.* **2014**, *48* (12), 1276-1284. DOI: 10.1080/02786826.2014.981330.

(41) Mutzel, A.; Rodigast, M.; Iinuma, Y.; Böge, O.; Herrmann, H. An improved method for the quantification of SOA bound peroxides. *Atmos. Environ.* **2013**, *67*, 365-369. DOI: 10.1016/j.atmosenv.2012.11.012.

(42) Lin, P.; Fleming, L. T.; Nizkorodov, S. A.; Laskin, J.; Laskin, A. Comprehensive molecular characterization of atmospheric brown carbon by high resolution mass spectrometry with electrospray and atmospheric pressure photoionization. *Anal. Chem.,* **2018**, *90* (21), 12493-12502. DOI: 10.1021/acs.analchem.8b02177.

(43) Phillips, S. M.; Smith, G. D. Light absorption by charge transfer complexes in brown carbon aerosols. *Environ. Sci. Technol. Lett.* **2014**, *1* (10), 382-386. DOI: 10.1021/ez500263j.

(44) Reichardt, C. Solvents and solvent effects in organic chemistry, 3 Edn., Wiley-VCH Verlag GmbH & Co. KGaA, 329–388, 2003.

(45) Hennigan, C. J.; McKee, M.; Pratap, V.; Boegner, B.; Reno, J.; Garcia, L.; McLaren, M.; Lance, S. M. DOI: 10.5194/egusphere-2023-854.

(46) Qin, Y.; Qin, J.; Zhou, X.; Yang, Y.; Chen, R.; Tan, J.; Xiao, K.; Wang, X. Effects of pH on light absorption properties of water-soluble organic compounds in particulate matter emitted from typical emission sources. *J. Hazard. Mater.*, **2022**, *424* (Pt C), 127688. DOI: 10.1016/j.jhazmat.2021.127688.

(47) Moschos, V.; Gysel-Beer, M.; Modini, R. L.; Corbin, J. C.; Massabò, D.; Costa, C.; Danelli, S. G.; Vlachou, A.; Daellenbach, K. R.; Szidat, S.; Prati, P.; Prévôt, A. S. H.; Baltensperger, U.; El Haddad, I. Source-specific light absorption by carbonaceous components in the complex aerosol matrix from yearly filter-based measurements. *Atmos. Chem. Phys.,* **2021**, *21* (17), 12809-12833. DOI: 10.5194/acp-21-12809-2021.

(48) Metsalu, T.; Vilo, J. ClustVis: a web tool for visualizing clustering of multivariate data using Principal Component Analysis and heatmap. *Nucleic Acids Res.* **2015**, *43* (W1), W566-570. DOI: 10.1093/nar/gkv468.

(49) Ma, J.; Ungeheuer, F.; Zheng, F.; Du, W.; Wang, Y.; Cai, J.; Zhou, Y.; Yan, C.; Liu, Y.; Kulmala, M.; Daellenbach, K. R.; Vogel, A. L. Nontarget screening exhibits a seasonal cycle of $PM_{2.5}$ organic aerosol composition in Beijing. *Environ. Sci. Technol.* **2022**, *56* (11), 7017-7028. DOI: 10.1021/acs.est.1c06905.

(50) Kuang, Y.; Shang, J.; Sheng, M.; Shi, X.; Zhu, J.; Qiu, X. Molecular composition of Beijing $PM_{2.5}$ brown carbon revealed by an untargeted approach based on gas chromatography and time-of-flight mass spectrometry. *Environ. Sci. Technol.* **2023**, *57* (2), 909-919. DOI: 10.1021/acs.est.2c05918.
